# The Role of Inflammatory Cytokines as Intermediates in the Pathway from Increased Adiposity to Disease

**DOI:** 10.1002/oby.23060

**Published:** 2021-01-24

**Authors:** Marita Kalaoja, Laura J. Corbin, Vanessa Y. Tan, Ari V. Ahola‐Olli, Aki S. Havulinna, Kristiina Santalahti, Niina Pitkänen, Terho Lehtimäki, Leo‐Pekka Lyytikäinen, Emma Raitoharju, Ilkka Seppälä, Mika Kähönen, Samuli Ripatti, Aarno Palotie, Markus Perola, Jorma S. Viikari, Sirpa Jalkanen, Mikael Maksimow, Veikko Salomaa, Marko Salmi, Olli T. Raitakari, Johannes Kettunen, Nicholas J. Timpson

**Affiliations:** ^1^ Computational Medicine Center for Life Course Health Research Faculty of Medicine University of Oulu Oulu Finland; ^2^ Biocenter Oulu University of Oulu Oulu Finland; ^3^ MRC Integrative Epidemiology Unit at University of Bristol Bristol UK; ^4^ Population Health Sciences Bristol Medical School University of Bristol Bristol UK; ^5^ Stanley Center for Psychiatric Research Broad Institute of MIT and Harvard Cambridge Massachusetts USA; ^6^ Analytic and Translational Genetics Unit Department of Medicine Massachusetts General Hospital Boston Massachusetts USA; ^7^ Institute for Molecular Medicine (FIMM) University of Helsinki Helsinki Finland; ^8^ Finnish Institute for Health and Welfare Helsinki Finland; ^9^ Medicity Research Laboratory and Institute of Biomedicine University of Turku Turku Finland; ^10^ Research Centre of Applied and Preventive Cardiovascular Medicine University of Turku Turku Finland; ^11^ Department of Clinical Chemistry Fimlab Laboratories Tampere Finland; ^12^ Department of Clinical Chemistry, Finnish Cardiovascular Research Center Tampere, Faculty of Medicine and Health Technology Tampere University Tampere Finland; ^13^ Department of Clinical Physiology Tampere University Hospital Tampere Finland; ^14^ Department of Clinical Physiology Finnish Cardiovascular Research Center Tampere Faculty of Medicine and Health Technology Tampere University Tampere Finland; ^15^ Department of Public Health University of Helsinki Helsinki Finland; ^16^ Program in Medical and Population Genetics Broad Institute of MIT and Harvard Cambridge Massachusetts USA; ^17^ Psychiatric and Neurodevelopmental Genetics Unit Department of Psychiatry Massachusetts General Hospital Boston Massachusetts USA; ^18^ Department of Neurology Massachusetts General Hospital Boston Massachusetts USA; ^19^ Department of Medicine University of Turku Turku Finland; ^20^ Division of Medicine Turku University Hospital Turku Finland; ^21^ Centre for Population Health Research University of Turku, Turku University Hospital Turku Finland; ^22^ Department of Clinical Physiology and Nuclear Medicine University of Turku Turku Finland

## Abstract

**Objective:**

This study aimed to investigate the role of cytokines as intermediates in the pathway from increased adiposity to disease.

**Methods:**

BMI and circulating levels of up to 41 cytokines were measured in individuals from three Finnish cohort studies (*n* = 8,293). Mendelian randomization (MR) was used to assess the impact of BMI on circulating cytokines and the impact of BMI‐driven cytokines on risk of obesity‐related diseases.

**Results:**

Observationally, BMI was associated with 19 cytokines. For every SD increase in BMI, causal effect estimates were strongest for hepatocyte growth factor, monocyte chemotactic protein‐1 (MCP‐1), and tumor necrosis factor–related apoptosis‐inducing ligand (TRAIL) and were as ratios of geometric means 1.13 (95% CI: 1.08‐1.19), 1.08 (95% CI: 1.04‐1.14), and 1.13 (95% CI: 1.04‐1.21), respectively. TRAIL was associated with a small increase in the odds of coronary artery disease (odds ratio: 1.03; 95% CI: 1.00‐1.06). There was inconsistent evidence for a protective role of MCP‐1 against inflammatory bowel diseases.

**Conclusions:**

Observational and MR estimates of the effect of BMI on cytokine levels were generally concordant. There was little evidence for an effect of raised levels of BMI‐driven cytokines on disease. These findings illustrate the challenges of MR when applied in the context of molecular mediation.

**Study Importance**

**What is already known?**

► Chronic low‐grade inflammation is a common feature of obesity and obesity‐related conditions such as insulin resistance and type 2 diabetes.
► Persistent dysregulation of the body’s inflammatory response has been implicated in several diseases, including cardiovascular disease, diabetes, and cancer.

**What does this study add?**

► We extend knowledge of the impact of increased adiposity (measured using BMI) on inflammatory profile beyond the “usual suspects” to 41 directly quantified cytokines and growth factors, providing estimates of effect derived from both observational and causal analyses.
► Our findings contribute to an emerging picture of a complex role for cytokines in immune‐mediated diseases and provide suggestive evidence for a detrimental role of TRAIL in coronary artery disease.

**How might these results change the direction of research or the focus of clinical practice?**

► C‐reactive protein is likely to remain a key biomarker for systemic inflammation in patients with increased adiposity, but as we begin to better understand their functions, other cytokines may serve as markers for more specific adiposity‐related pathophysiology.
► Methodological developments are required in order to increase our confidence in the use of Mendelian randomization to evaluate the causal effect on disease of signaling molecules, such as cytokines, because of the likely pleiotropic nature of instruments.

## Introduction

Obesity is a major public health problem, the burden of which extends across multiple organ systems. Increased adiposity, frequently assessed using BMI, has been directly implicated in the development of diseases such as cardiovascular disease (1), diabetes (1), and some cancers (2). However, the mechanisms by which raised BMI affects disease are not fully understood. Studying intermediate phenotypes reflective of physiological function can provide valuable insight into such complicated etiologic disease pathways as these (3).

Chronic low‐grade inflammation as characterized by abnormal cytokine production and the activation of a network of inflammatory signaling pathways is a common feature of obesity and obesity‐related conditions, such as insulin resistance and type 2 diabetes (4). Although inflammation is an important part of the human immune response and plays a key role in protecting the body against pathogens, inflammation can also be harmful. Persistent dysregulation of the body’s inflammatory response has been implicated in several diseases, including cardiovascular disease (5), diabetes (6, 7), and cancer (8, 9). In obesity, inflammatory signals appear to disrupt insulin action, leading to insulin resistance (10), and it has been hypothesized that the development of a systemic inflammatory state in those with overweight contributes to obesity‐associated pathophysiological consequences (11).

As signaling molecules with a key role in regulating inflammation, circulating cytokine levels provide a useful readout of inflammatory function. Tumor necrosis factor alpha was the first proinflammatory cytokine to be associated with obesity‐linked insulin resistance (12). Since then, a number of other proinflammatory cytokines, including several interleukins (ILs), have been identified as potential mediators in the systemic inflammatory response to obesity (13). However, with most studies of increased adiposity and inflammation focusing on a small number of relatively well‐known proinflammatory molecules, the relevance of many other cytokines to this pathway remains uncertain. Furthermore, it is understood that in observational analyses, even with careful study design, confounding, reverse causation, and other biases limit the extent to which causal inference is possible (14). One approach that is useful in trying to address these problems is the use of genetic association results within a Mendelian randomization (MR) framework (14). In MR analyses, genetic variants act as an approximation to instrumental variables (IVs) to enable an evaluation of the causal effect of the exposure on the outcome (14). MR analyses, in which BMI represents the exposure and C‐reactive protein (CRP) the outcome, were among the first to use this approach to provide evidence in support of a causal role for increased adiposity in promoting low‐grade inflammation (15). However, there appear to be few, if any, examples of the same approach being applied to collections including multiple cytokine measurements.

More commonly, MR studies have been undertaken looking at the downstream effects of specific inflammatory molecules. Because of the need for genetic variants to act as proxies for the exposure, such analyses have been limited to the study of cytokines and other inflammatory biomarkers for which these exist. CRP has been the focus of much attention, but despite its strong association with inflammation and its observational association with disease, evidence for a causal effect of CRP on disease is limited. One proposed explanation for this, given in the context of breast cancer, is that CRP is only linked to disease as a “cytokine reporter,” hence there is no relationship to non–“responsive‐mode” variation in CRP levels driven by polymorphisms in the gene (16). In contrast, a number of MR studies focused on ILs as an exposure, have found evidence for a causal effect of IL‐6 and/or IL‐1 on a small number of cardiovascular and autoimmune‐type diseases, as well as schizophrenia (17, 18, 19, 20).

Therefore, although evidence on the possible role of inflammation in pathologies related to increased adiposity is accumulating, the relevance of many less well‐characterized cytokines is unclear. The recent publication of a genome‐wide association study (GWAS) for 41 cytokines (21) conducted in a Finnish population represents an advance in this field, enabling a much broader range of cytokines and their impact on disease to be assessed. Within three population‐based studies, we employ a two‐step MR analysis (22) to investigate the relationship between BMI, circulating levels of the 41 cytokines, and disease. Included in this work is a rerun of the published cytokine GWAS (21), removing BMI as a covariate from the model, which was conducted in order to derive appropriate genetic instruments for circulating cytokines.

## Methods

### Study design

**Figure 1:**
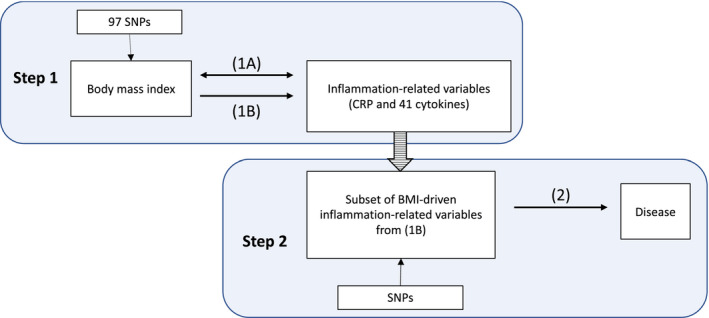
Study overview. (1A) represents the analysis of the observational association between BMI and the inflammation‐related variables, whereas (1B) represents the one‐sample Mendelian randomization (MR) analysis of the effect of BMI on the inflammation‐related variables. (2) Represents the two‐sample MR analysis of the effect of BMI‐driven inflammation‐related variables on relevant disease outcomes. [Color figure can be viewed at wileyonlinelibrary.com]

This study has three main components as outlined in Figure 1: (1A) analysis of the observational association between BMI and cytokines using individual level data from three Finnish cohort studies; (1B) analysis of the causal effect of BMI on cytokines in a one‐sample MR framework, using the same individual level data (an equivalent two‐sample style MR analysis is presented in the online Supporting Information Methods); and (2) analysis of the causal effect of BMI‐driven cytokines on relevant disease outcomes in a two‐sample MR framework. By way of comparison, results for the more widely studied CRP are presented alongside those for the inflammatory cytokines, although it should be noted that CRP was measured independently from the cytokines. Herein, the term “inflammation‐related variables” will be used to describe the set of inflammatory cytokines plus CRP.

### Study populations

Individual level data analyses (1A and 1B in Figure 1) were examined in a cross‐sectional study within the Cardiovascular Risk in Young Finns Study (YFS) and the FINRISK 1997 and 2002 cohorts. YFS (https://youngfinnsstudy.utu.fi/) is a multicenter follow‐up study with randomly chosen subjects from the Finnish cities of Helsinki, Kuopio, Oulu, Tampere, and Turku and their rural surroundings. FINRISK surveys (https://thl.fi/en/web/thlfi-en/research-and-expertwork/population-studies/the-national-finrisk-study) are population‐based cross‐sectional studies conducted every 5 years to monitor the levels of chronic disease risk factors in Finland. Each survey includes 25‐ to 74‐year‐old randomly chosen subjects from five geographical areas of Finland. For further details of the cohorts used, see the Supporting Information Methods. The present study includes unrelated individuals from these cohorts with both cytokine measurements and genome‐wide genotype data. Further details regarding cytokine quantification, genotyping, and subsequent imputation can be found in the Supporting Information Methods and are as described previously (21). In YFS (*n* = 1,980) and FINRISK 2002 (*n* = 1,705), 48 cytokines were measured using Bio‐Rad’s premixed Bio‐Plex Pro Human Cytokine 27‐plex and 21‐plex assays. In FINRISK 1997 (*n* = 4,608), a custom‐selected 20‐cytokine plex was used. Cytokines with >90% of values missing were excluded from all analyses. A full list of the measured cytokines is provided in Supporting Information Table S1. These cohort data were also used to generate a revised version of the previously published GWAS of 41 inflammatory cytokines (21), without fitting BMI as a covariate in the model (see the Supporting Information Methods). This was done in order to avoid the possibility of collider bias in the subsequent two‐sample MR analysis (step 2 in Figure 1). Data on several potential confounders were used in sensitivity analyses (see the Supporting Information Methods).

### Selection of genetic variants for MR analyses

Ninety‐seven independent single‐nucleotide polymorphisms (SNPs) that were associated with BMI at genome‐wide significance (*P *<* *5* *×* *10^−8^) were selected from a meta‐GWAS for BMI (*n *=* *339,224) (23). The genetic instruments for the BMI‐associated cytokines taken forward to analysis step 2 were selected based on the results of our rerun of the previously published GWAS of 41 inflammatory cytokines (*n *=* *8,337) (21). To maximize the number of available instruments for each cytokine, a list of cytokine‐associated SNPs (*P *<* *5* *×* *10^−8^) was first cross‐matched to the corresponding GWAS summary statistics for each disease outcome in turn. Then, taking the subset of SNPs that were present (or had a suitable proxy with linkage disequilibrium [LD], *r* ^2^ **>* *0.8) in both data sets, an independent set of instruments was generated using an LD clumping procedure with an *r* ^2^ threshold of 0.01 (see Supporting Information Methods for full details). This procedure was implemented using the TwoSampleMR R package (24) (version 0.4.26).

For CRP, we used as instruments a set of three SNPs (rs3093077 [A/C], rs1205 [T/C], and rs1130864 [A/G]) that has previously been shown to represent common variation within the *CRP* gene associated with different circulating levels in populations of European descent (25).

### Statistical analyses

In the observational analysis and first step of the MR (1A and 1B in Figure 1), we penalized results given the 41 tests undertaken (*P *<* *0.001 can be considered equivalent to *P *<* *0.05). No such penalization was applied in the second step of the MR in which we took *P *<* *0.05 as evidence of association.

#### Part 1A: The observational association between BMI and the inflammation‐related variables

Observational associations of BMI with levels of 41 cytokines and CRP (1A in Figure 1) were assessed in the three cohorts using linear regression. Cytokine distributions were first normalized with rank‐based inverse normal transformation (RNT). The transformed phenotypes were then regressed on age, sex, and 10 principal components and model residuals extracted. A second RNT was then performed on the residuals. Associations of residuals with BMI were assessed using linear regression. CRP measures underwent the same transformation and covariate adjustment process. Effect estimates for cytokines and CRP are given in normalized SD units per *z*‐scored SD higher BMI. All observational analyses were conducted separately in the three cohorts and combined in a fixed effects meta‐analysis. Supplementary analyses relating to potential confounders are described in the Supporting Information Methods.

#### Part 1B: One‐sample MR analysis of the effect of BMI on the inflammation‐related variables

A one‐sample MR analysis (1B in Figure 1) was carried out using a weighted genetic risk score (GRS) generated based on the 97 SNPs selected as instruments for BMI (see the Supporting Information Methods). Two‐stage least squares analysis was used to obtain estimates of the association of BMI with each inflammatory variable (using the same RNT residuals as were derived previously). Analyses were conducted separately in the three cohorts using the ivreg‐function from the R package AER (26) and then were combined in a meta‐analysis using the rma‐function from the R package metaphor (27). Effect estimates are given in normalized SD units per SD higher BMI. We compared the instrumental variable estimates with those from observational linear regression using the Durbin form of the Durbin‐Wu‐Hausman statistic (28). For cytokines in which one‐sample MR yielded suggestive evidence for BMI effect (*P *<* *0.05), analyses were rerun using natural log‐transformed values (adjusting for age and sex) to aid interpretation (1 was added to each value prior to transformation to account for zero values).

For completeness, the causal associations between BMI and levels of inflammatory variables assessed in the one‐sample framework described above were also evaluated using a two‐sample framework. Causal estimates were derived using the Wald ratio method and estimates were combined using the inverse‐variance weighted method. Full details of this analysis, including sensitivity analyses, are presented in the Supporting Information Methods.

#### Part 2: Two‐sample MR analysis of the effect of BMI‐driven inflammation‐related variables on relevant disease outcomes

The criteria for inclusion in this second step, two‐sample MR analysis (step 2 in Figure 1) was *P *<* *0.001 in the first step analysis, such that only those cytokines for which there was strongest (a priori) evidence of BMI effect were taken forward. In this analysis, SNP‐exposure associations were taken from our rerun of the 41‐cytokine GWAS and for CRP, from a recent meta‐GWAS (*n *>* *200,000) (29). SNP‐outcome associations for relevant disease outcomes were derived from a number of published GWASs conducted in populations of European or mixed ancestry. There was no sample overlap between exposure and outcome data sources.

Diseases were considered relevant if there was existing observational evidence of their association with BMI and/or cytokines. From this selection, diseases were included in the analysis if there existed, within MR‐Base (24), summary statistics for a GWAS of that disease with a sample size of at least 50,000 subjects (Supporting Information Table S2). Diseases included as outcomes were coronary artery disease (CAD), type 2 diabetes, inflammatory bowel disease (IBD) (plus Crohn disease [CD] and ulcerative colitis [UC] as separate traits), rheumatoid arthritis, Alzheimer disease, schizophrenia, breast cancer, and ovarian cancer. For IBD and associated traits, we extracted results from both Immunochip‐based and GWAS‐based meta‐analyses.

For each instrument (SNP), causal estimates were derived using the Wald ratio method, and when multiple instruments were available, estimates were combined using the inverse‐variance weighted method to provide a single causal estimate of the exposure‐outcome association (30). Methods typically used to test for pleiotropy, such as MR‐Egger (31), were not conducted as these lack power with small numbers of SNPs. However, heterogeneity tests were used to give an indication of the variability of the estimates across SNPs. Statistical analyses were performed using R version 3.5.3.

## Results

Baseline characteristics of the participants in YFS and in FINRISK 1997 and 2002 are shown in Table 1 and cytokine summary statistics are shown in Supporting Information Table S3. A total of 8,293 individuals with BMI and cytokines measured were included in this study from across the three cohorts (YFS, FINRISK 1997, and FINRISK 2002). The mean age (SD) of individuals in YFS, FINRISK 1997, and FINRISK 2002 was 38 (5), 48 (13), and 60 (6) years, respectively. The mean BMI (SD) of individuals in YFS, FINRISK 1997, and FINRISK 2002 was 26 (5), 27 (5), and 28 (4) kg/m^2^, respectively.

**TABLE 1:** Characteristics of participants in the three cohorts

		Young Finns Study, *N *= 1,980	FINRISK 1997, *N* = 4,608	FINRISK 2002, *N* = 1,705
***Continuous variables***	**Total, *N***	**Mean (SD)**	**Mean (SD)**	**Mean (SD)**
**Age (y)**	8,293	37.7 (5.0)	47.8 (13.3)	60.3 (6.1)
**BMI (kg/m^2^)**	8,293	26.0 (4.8)	26.6 (4.5)	27.9 (4.5)
**Alcohol (g/wk)**	8,091	80.1 (121.1)	70.9 (126.4)	78.2 (135.1)
**C‐reactive protein (mg/L)**	8,227	1.8 (3.8)	2.5 (6.1)	2.9 (6.2)
***Categorical variables***	***N***	**% (*N*)**	**% (*N*)**	**% (*N*)**
**Gender**				
**Male**	4,059	45.7 (905)	49.9 (2,300)	50.1 (854)
**Female**	4,234	54.3 (1,075)	50.1 (2,308)	49.9 (851)
**Socioeconomic status**				
**Low**	1,926	4.9 (96)	29.2 (1,334)	29.8 (496)
**Average**	3,422	70.7 (1,393)	32.7 (1,493)	32.2 (536)
**High**	2,856	24.4 (481)	38.2 (1,745)	37.9 (630)
**Smoking**				
**Current**	1,825	18.6 (365)	25.1 (1,150)	18.3 (310)
**Ex/never**	4,813	81.4 (1,600)	74.9 (3,427)	81.7 (1,386)

Results from the rerun of the previously published GWAS of 41 inflammatory cytokines are available at the University of Bristol data repository, data.bris, at: https://doi.org/10.5523/bris.3g3i5smgghp0s2uvm1doflkx9x. Removing BMI as a covariate in the model had a minor impact on the GWAS results. The correlations between betas derived from the two models (with and without BMI fitted) were between 0.77 and 1 (median 0.99). Of the 27 SNP‐cytokine associations previously reported (21), all were present in the BMI‐unadjusted results with similar betas (Supporting Information Figure S1).

### Part 1A: The observational association between BMI and the inflammation‐related variables

**Figure 2:**
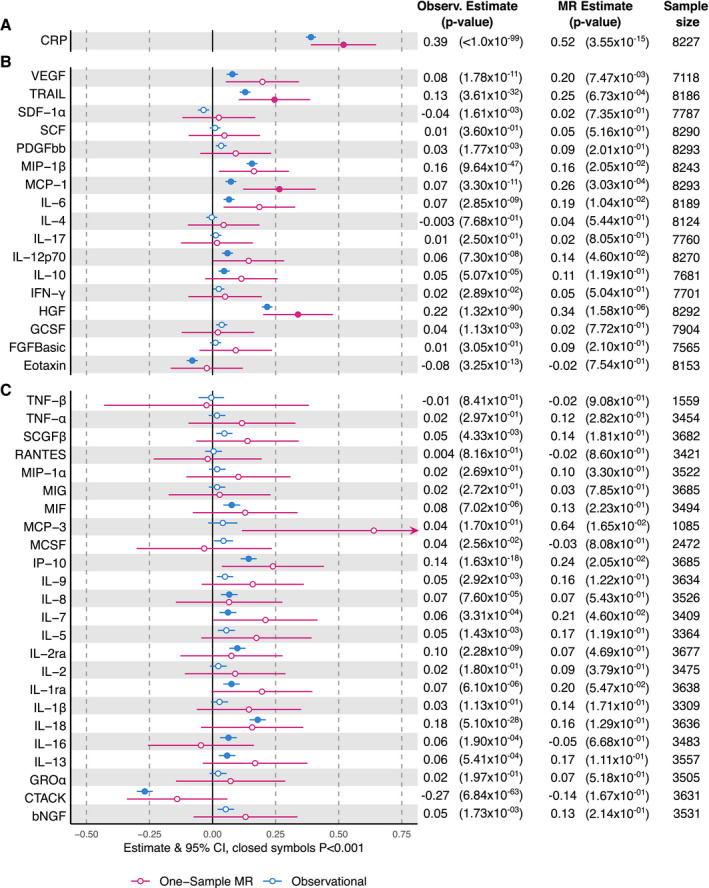
Observational (Observ.) and Mendelian randomization (MR) associations of BMI and inflammation‐related variables. Effect estimates are given in normalized SD units per 1‐SD‐higher BMI. Estimates represent meta‐analyzed results. (**A**) CRP result (YFS, FINRISK 1997, FINRISK 2002). (**B**) Results for cytokines available in all three cohorts (YFS, FINRISK 1997, FINRISK 2002). (**C**) Results for cytokines available only in two cohorts (YFS, FINRISK 2002).

The age‐ and sex‐adjusted observational associations between BMI and cytokines for YFS, FINRISK 1997, and FINRISK 2002 are shown in Figure 2. In meta‐analyses, BMI was associated with 19 cytokines (CTACK [C‐C motif chemokine 27], eotaxin, hepatocyte growth factor [HGF], IL‐10, IL‐12p70, IL‐13, IL‐16, IL‐18, IL‐1ra, IL‐2ra, IL‐6, IL‐7, IL‐8, IP‐10, monocyte chemotactic protein‐1 [MCP‐1], macrophage migration inhibitory factor [MIF], macrophage inflammatory protein 1‐beta [MIP‐1β], tumor necrosis factor–related apoptosis‐inducing ligand [TRAIL], and vascular endothelial growth factor [VEGF]) and CRP (Figure 2). Estimates were largely consistent across cohorts (Supporting Information Figure S2) and with and without additional adjustment for potential confounding factors (Supporting Information Figure S3). Confounder association results are presented in the Supporting Information (Tables S4‐S7).

### Part 1B: One‐sample MR analysis of the effect of BMI on the inflammation‐related variables

A one unit increase in the BMI GRS was associated with a 4.36 kg/m^2^ (95% CI: 3.78‐ 4.93; *P *=* *7.34* *×* *10^−50^) increase in BMI (Supporting Information Table S9), giving a median F‐statistic of 66 in YFS, 102 in FINRISK 1997, and 29 in FINRISK 2002 (Supporting Information Table S10). When compared with the relationship between observed BMI and potential confounders (Supporting Information Table S4), the association of the BMI GRS with confounders was substantially diminished (Supporting Information Table S9). The inflammation‐related variables with the strongest evidence of BMI effect were CRP, HGF, MCP‐1, and TRAIL (Figure 2, Supporting Information Figure S4). Effect sizes derived by exponentiating the natural log‐transformed betas presented in Supporting Information Table S11 and, therefore, expressed as the ratio of geometric means of circulating CRP, HGF, MCP‐1, and TRAIL per 1‐SD increase in BMI were 1.39 (95% CI: 1.27‐1.51), 1.13 (95% CI: 1.08‐1.19), 1.08 (95% CI: 1.04‐1.14), and 1.13 (95% CI: 1.04‐1.21), respectively; this approximates to an increase in circulating CRP of 0.44 mg/L, and an increase in HGF, MCP‐1, and TRAIL of 50.9 pg/mL, 2.8 pg/mL and 14.7 pg/mL, respectively. There was no clear evidence of a departure of instrumental variable‐derived estimates from those derived from observational analyses (Supporting Information Table S10). A further seven cytokines had causal effect estimates that were different from zero but failed to satisfy our inclusion criteria for the step two analysis (IL‐12p70, IL‐6, IL‐7, IP‐10, MCP‐3, MIP‐1β, and VEGF).

### Part 2: Two‐sample MR analysis of the effect of BMI‐driven inflammation‐related variables on relevant disease outcomes

Causal effects on disease were estimated for the four inflammation‐related variables, which met the inclusion criteria for the second step (CRP, HGF, MCP‐1, and TRAIL). Overall, we identified 3, 6, and 33 SNPs as instruments for HGF, MCP‐1, and TRAIL, respectively; effect estimates for these and the three CRP SNPs used are shown in Supporting Information Table S12. The actual set of instruments used in each analysis was dependent on which SNPs were present in the relevant disease GWAS (see Supporting Information Tables S13‐S16 for lists of instruments used in each analysis). We observed considerable heterogeneity in the effect estimates for rs12075 on MCP‐1 across the three cohorts (Supporting Information Table S17). We hypothesize that the lack of association in the FINRISK 2002 cohort is likely due to the use of heparin as an anticoagulant (32). However, given the relatively small contribution of this cohort to the total sample, we anticipate that the impact of this difference on the meta‐analyzed SNP effect estimate used in the MR is low.

**Figure 3:**
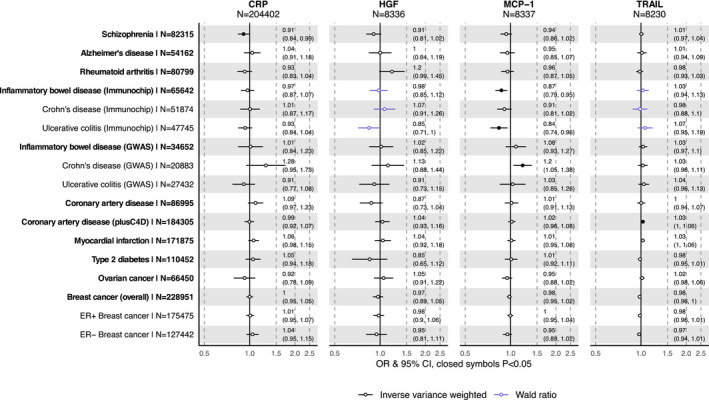
Mendelian randomization associations of BMI‐driven inflammation‐related variables and disease outcomes. Estimates correspond to the odds ratio (OR) per unit increase in natural log‐transformed CRP or per normalized SD increase in HGF, MCP‐1, and TRAIL. [Color figure can be viewed at wileyonlinelibrary.com]

**Figure 4:**
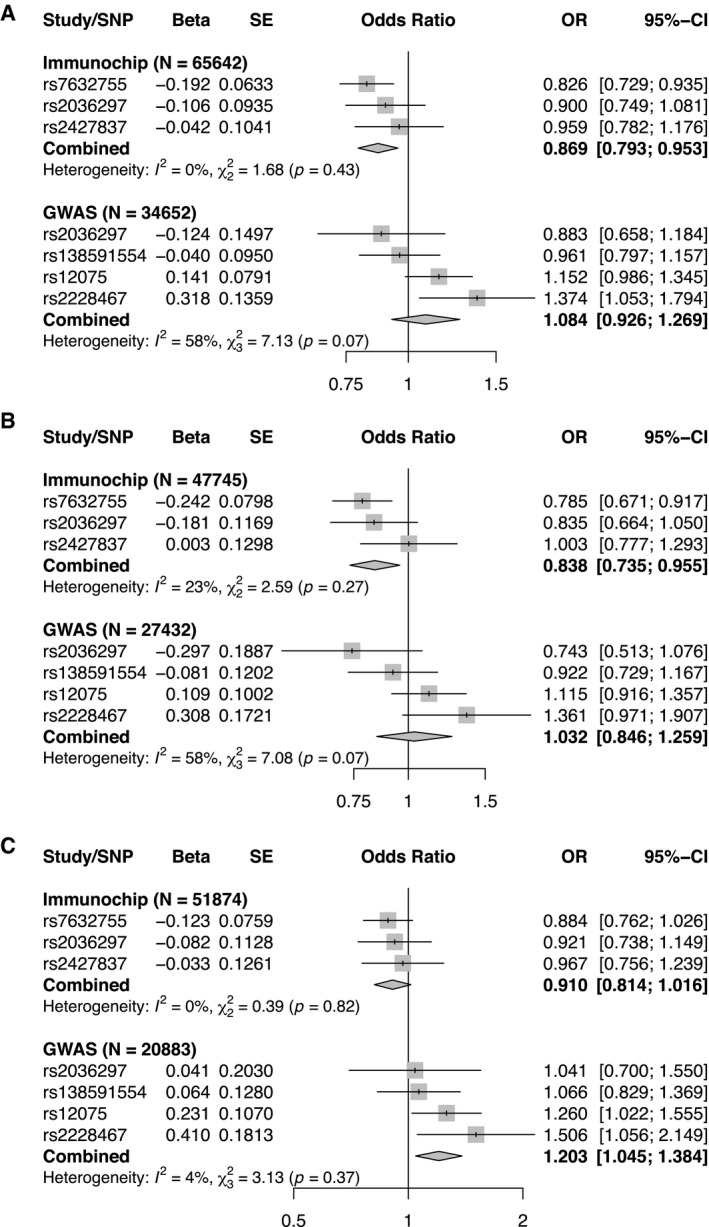
SNP‐specific Mendelian randomization associations for MCP‐1 and inflammatory bowel disease (IBD), ulcerative colitis (UC), and Crohn disease (CD). (**A**) SNP‐specific associations between circulating MCP‐1 and odds of IBD. Estimates correspond to the odds ratio for IBD using either Immunochip data (*N* cases = 31,665, *N* controls = 33,977) or GWAS data (*N* cases = 12,882, *N* controls = 21,770) per normalized SD increase in circulating MCP‐1 (*N* = 8,337) levels. (**B**) SNP‐specific associations between circulating MCP‐1 and odds of UC. Estimates correspond to the odds ratio for UC using either Immunochip data (*N* cases = 13,768, *N* controls = 33,977) or GWAS data (*N* cases = 6,968, *N* controls = 20,464) per normalized SD increase in circulating MCP‐1 (*N* = 8,337) levels. (**C**) SNP‐specific associations between circulating MCP‐1 and odds of CD. Estimates correspond to the odds ratio for CD using either Immunochip data (*N* cases = 17,897, *N* controls = 33,977) or GWAS data (*N* cases = 5,956, *N* controls = 14,927) per normalized SD increase in circulating MCP‐1 (*N* = 8,337) levels.

For CRP, we saw evidence of association with schizophrenia only, with an odds ratio (OR) of 0.91 (95% CI: 0.84‐0.99; *P *=* *0.03) per unit change in natural log‐transformed CRP levels (Figure 3, Supporting Information Table S18). For MCP‐1, we detected an association with IBD using Immunochip summary statistics such that the ORs of disease per normalized SD change in MCP‐1 was 0.87 (95% CI: 0.79‐0.95; *P *=* *0.003) (Figures 3 and 4A, Supporting Information Table S18). Corresponding effect estimates for the IBD subtypes of CD and UC were similar (0.91 [95% CI: 0.81‐1.02; *P *=* *0.09] and 0.84 [95% CI: 0.74‐0.96; *P *=* *0.008], respectively) (Figures 3 and 4B‐4C, Supporting Information Table S18). Effect estimates derived from the GWAS data sets for these same diseases did not show the same protective effect. Results were largely consistent with those from the Immunochip analysis for the single shared SNP (rs2036297), but overall estimates for IBD and UC were null, whereas the association between MCP‐1 and CD (OR 1.2; 95% CI: 1.05‐1.38; *P *=* *0.01) was in the opposite direction to that observed using the Immunochip data set (Figures 3 and 4, Supporting Information Table S18). We found evidence of a small effect of TRAIL on CAD with OR of 1.03 (95% CI: 1.00‐1.06; *P *=* *0.04) using the CARDIoGRAMplusC4D GWAS data but no apparent effect when using the smaller European‐only CARDIoGRAM GWAS data (OR 1.00; 95% CI: 0.94‐1.07; *P *=* *0.91) (Figure 3, Supporting Information Figure S7 and Table S18).

## Discussion

In this study, we compared estimates from observational analyses with those from MR (1) to allow inference to be drawn about the causal relationship between adiposity and inflammation and (2) to evaluate the subsequent impact of BMI‐driven cytokines on relevant disease outcomes. We can now expand our understanding of the impact of BMI on the inflammatory profile beyond the “usual suspects” (e.g., CRP, IL‐6) in a systematic and robust way that makes use of new data. Using a two‐step MR design, we were able to partially replicate previous associations and contribute new knowledge. Overall, we saw little evidence for a detrimental effect on health of raised levels of the BMI‐driven cytokines examined. However, we cannot rule out the possibility that these cytokines may have small effects on disease risk that are not detectable here because of limited power. Our findings contribute to the emerging literature that points toward a complex role for cytokines in immune‐mediated diseases (33) and provide suggestive evidence for a detrimental role of TRAIL in CAD.

In observational analyses, the majority of cytokines within the IL subclass showed a positive association, with BMI having the strongest signal observed for IL‐18. Outside of the IL subclass, strong effects were also observed for HGF, TRAIL, and MIP‐1β. Although the majority of cytokines were found to be positively associated with BMI, both eotaxin and CTACK (CCL27) showed decreased levels with increased BMI. In the one‐sample MR framework, evidence was strongest for a BMI effect on HGF, MCP‐1, and TRAIL. These effects are commensurate with reductions in both HGF (34) and MCP‐1 (35) seen following weight loss. Meanwhile, TRAIL has previously been proposed as having a role in obesity and weight gain (36) and has been linked to a proinflammatory response in adipocytes (37). Although the effect estimates derived from the MR analysis were in most cases similar to those derived observationally, the relative reduction in power and the conservative nature of our multiple testing correction meant that only three cytokines met the criteria set for inclusion in the second step. Retrospective power analyses (38) showed that, when we had data for three cohorts (approximately 8,000 individuals), our analyses had 80% power to detect effect sizes in excess of 0.19 SD (α = 0.05), which suggests reasonable power across the range of effects seen observationally (0.05‐0.27 SD). However, when data were available for only two cohorts (approximately 3,500 individuals), only the largest effect sizes (>0.30 SD) were likely detectable with the same level of statistical confidence. We would anticipate that with greater statistical power, several other cytokines would be added to the list of BMI‐associated molecules.

Restricting our attention to HGF, MCP‐1, and TRAIL, we went on to consider whether these cytokines could be causally related to BMI‐associated disease outcomes. Increased levels of TRAIL appeared to be associated with a small increase in the odds of CAD but only when using the larger CARDIoGRAM‐plusC4D GWAS result. Although marginal, particularly within the context of the number of tests performed, this effect is of interest given the complex relationship of TRAIL and its receptor with atherosclerosis and vascular biology. TRAIL and its receptors are expressed in both physiological and pathological arterial walls (39). There is evidence that TRAIL signaling can either protect against or promote the formation of atherosclerotic lesions (40). The complex relationship of TRAIL with CAD may be due to the existence of different receptor types that either initiate apoptosis (TRAIL‐R1/DR4 and R2/DR5) or promote apoptosis by acting as decoy proteins for TRAIL binding (TRAIL‐R3/DcR1, R4/DcR2, and osteoprotegerin) (40). Our observation that TRAIL does not appear to have a downstream effect on metabolic disease is important given conflicting evidence concerning its role in inflammatory diseases such as type 2 diabetes (37). In response to studies (mainly mouse work) showing a protective effect of TRAIL against increased adiposity/diabetes, both TRAIL and its receptor TRAIL‐R have been proposed as therapeutic targets. However, the lack of association of TRAIL with any downstream metabolic disease here suggests more research is needed to fully understand the role of this molecule.

Using summary statistics from a large Immunochip study, MCP‐1 showed consistent associations with IBD and its subtypes (UC and CD), such that increased levels of the cytokine appear to decrease odds of disease. IBDs are immunologically mediated diseases characterized by chronic relapsing inflammation of the intestine. MCP‐1 is a chemokine that plays an important role in the recruitment of monocytes and macrophages from the bloodstream to inflamed tissues. Early studies involving intestinal biopsy specimens from patients with IBD showed increased *MCP‐1* mRNA expression in inflamed tissues, which in some cases was considered to be evidence for a pathological role of MCP‐1 in the disease (41, 42). However, more recently, an immunosuppressive role for MCP‐1 has been proposed and its therapeutic potential explored (43). This apparent uncertainty in the role of MCP‐1 in IBD is also reflected in the MR results in this study. Using summary statistics derived instead from the GWAS study of IBD, UC, and CD, no effect was seen of MCP‐1 on IBD and UC, whereas for CD, increased levels of MCP‐1 were found to increase odds of disease. These contradictory results point to important limitations of MR in this context. Cytokines are a network of signaling molecules and it would be naïve to imagine that we can instrument single cytokines and describe their impact on disease in isolation.

One specific issue potentially complicating interpretation is pleiotropy. The idea that genetic variants are associated with levels of single cytokines seems unrealistic and instead what we expect is that pleiotropy (i.e., genetic variants associated with more than one cytokine in pathway effects or as independent and horizontal events) is more likely. If pleiotropy is present in the form of independent pathway effects (horizontal), our MR estimates can be biased in specific conditions (44). Many of the SNPs we use as instruments for the cytokines are in *trans* and as such, we presume their effect must go through several pathways, opening the possibility that those pathways influence the outcome independently of the target exposure (44). The inconsistency that we see in results from the GWAS and Immunochip studies for MCP‐1 and IBD/UC/CD may be indicative of this issue. The instruments identified for MCP‐1 include variants in or near genes encoding chemokine receptors that have multiple ligands. Two SNPs present as instruments only in the GWAS‐based analysis (rs12075 in *ACKR1* and rs2228467 in *ACKR2*) generated estimates in the opposite direction to most other variants leading to heterogeneity in the estimates (*P *< 0.10 for IBD and UC). As anticipated, given their function, we found evidence that rs12075 and rs2228467 are associated with eight and four other cytokines (*P *<* *0.05) in our data, respectively. One potential strategy for reducing the impact of pleiotropy could be to restrict analyses to *cis* variants only, as these effects are likely closer to the biology of the molecular trait because of their genomic proximity. However, this would reduce the power of the MR because of the small number of instruments that remain.

Although various strategies have been developed to assess and minimize the impact of pleiotropy in MR (44), this is just one of a number of possible sources of bias, another being latent population structure. It seems likely that at least some of the disparities we see in results both within and out with the realm of MR reflect properties of the instruments employed. Other more subtle issues also exist. In particular, there is potential for SNP‐cytokine associations to vary because of blood collection protocols (as seen here for rs12075 and MCP‐1) and the possibility that SNP‐cytokine associations are driven by the impact of genetic variants on cytokine conformation rather than by actual changes in concentration. Furthermore, for two‐sample MR analyses to be valid, the two samples should be from the same underlying population. We did not use sex‐specific cytokine instruments for the female‐specific cancers, which could lead to biased estimates; however, we did not find evidence of marked sex‐differences for the cytokine instruments (Supporting Information Table S19A‐S19B). It should also be noted that this work is conducted in Finnish and European populations and that further work is needed to evaluate the extent to which our findings are generalizable to other populations, including those of wider European and non‐European ancestry.

In this study of the effect of variation in cytokine levels on disease, given *N *=* *50,000 (our threshold for inclusion) and assuming a 1:2 ratio of cases to controls, we had 80% power to detect an OR of 1.21, 1.19, and 1.05 for HGF, MCP‐1, and TRAIL, respectively (45). In this context, we do not see strong evidence for associations between BMI‐driven cytokines and risk of a collection of diseases often associated with increased adiposity. Although this result could be interpreted as evidence that inflammation is not a mediator of the relationship between increased adiposity and the set of disease outcomes studied, it is important to consider the context of these findings. In MR, results can be interpreted as a reflection of the impact of lifelong (chronic) exposure to a risk factor (in this case, raised cytokine levels) on disease. Taken in this way, results are not able to tell us about the role of these same cytokines when in a state of acute or chronic inflammation. For example, although we see some evidence for a protective effect against IBD of chronically raised levels of MCP‐1, the increase in MCP‐1 levels that reflect the inflammatory response to increased adiposity are unlikely to have the same downstream consequences. Furthermore, this work concentrates on circulating levels of cytokines, though it is likely that tissue‐specific expression of these molecules is important.

This study is the first MR analysis to estimate the causal effect of BMI on a comprehensive panel of inflammatory cytokines and the subsequent impact of BMI‐driven cytokines on relevant disease outcomes. Observationally, we see a broad signature of increased BMI on circulating cytokines. The concordance we observe between observational and MR estimates of the effect of BMI on circulating cytokines suggests a biologically meaningful relationship between BMI and inflammation and minimal influence of confounders. Although circulating cytokines provide a useful readout of the inflammatory response to increased adiposity, in order to provide a definitive answer to the question of the role of cytokines in disease, results from several different approaches with unrelated sources of bias, including challenge/intervention studies, need to be integrated.

## Funding agencies

NJT, LJC, and VYT work in the Medical Research Council (MRC) Integrative Epidemiology Unit at the University of Bristol, which is supported by the MRC (MC_UU_00011) and the University of Bristol. NJT is a Wellcome Trust Investigator (202802/Z/16/Z) and works within the University of Bristol National Institute for Health Research Biomedical Research Centre. LJC is supported by NJT’s Wellcome Trust Investigator grant (202802/Z/16/Z). NJT and VYT are supported by the Cancer Research UK Integrative Cancer Epidemiology Programme (C18281/A19169). JK is supported by the Academy of Finland (297338 and 307247) and the Novo Nordisk Foundation (NNF17OC0026062). MK was funded by Biocenter Oulu. VS is supported by the Finnish Foundation for Cardiovascular Research. The Young Finns Study has been financially supported by the Academy of Finland (grants 322098, 286284, 134309 [Eye], 126925, 121584, 124282, 129378 [Salve], 117787 [Gendi], and 41071 [Skidi]); the Social Insurance Institution of Finland; Competitive State Research Financing of the Expert Responsibility area of Kuopio, Tampere and Turku University Hospitals (grant X51001); Juho Vainio Foundation; Paavo Nurmi Foundation; Finnish Foundation for Cardiovascular Research; Finnish Cultural Foundation; The Sigrid Juselius Foundation; Tampere Tuberculosis Foundation; Emil Aaltonen Foundation; Yrjö Jahnsson Foundation; Signe and Ane Gyllenberg Foundation; Diabetes Research Foundation of Finnish Diabetes Association; EU Horizon 2020 (grant 755320 for TAXINOMISIS and grant 848146 for TO‐AITION); European Research Council (grant 742927 for MULTIEPIGEN project); Tampere University Hospital Supporting Foundation; and the Finnish Society of Clinical Chemistry. SR is supported by the Academy of Finland Center of Excellence in Complex Disease Genetics (grant 312062), Academy of Finland (grant 285380), the Finnish Foundation for Cardiovascular Research, the Sigrid Juselius Foundation, and University of Helsinki HiLIFE Fellow grant. MS is funded by Academy of Finland (grant 141136).

## Disclosure

VS has consulted for Novo Nordisk and Sanofi and has received honoraria from these companies. He also has ongoing research collaboration with Bayer Ltd (all unrelated to the present study). The other authors declared no conflict of interest.

## Supporting information

Supplementary Material

Table S13‐S16
