# Supplementary material for: The Role of Inflammatory Cytokines as Intermediates in the Pathway from Increased Adiposity to Disease
Source: Obesity (Silver Spring). 2021 Jan 24;29(2):428–37. doi: 10.1002/oby.23060 (PMC8614117; doi:10.1002/oby.23060)
Supplement: Supplementary file 1 — Supplementary Material [file OBY-29-428-s001.pdf]

SUPPLEMENTAL DATA FOR:

The role of inflammatory cytokines as intermediates in the pathway from increased adiposity to disease

Table of Contents

Supplemental Methods ... 3

Cohort descriptions ... 3

The Cardiovascular Risk in Young Finns Study ... 3

FINRISK ... 3

Genotypes ... 3

Laboratory analyses ... 3

Cytokine GWAS ... 4

Potential confounders ... 4

Selection of genetic variants for cytokines ... 4

Derivation of body mass index (BMI) genetic risk score (GRS) ... 5

Causal associations between BMI and levels of inflammatory variables: two-sample MR ... 5

Power calculations ... 6

Supplemental Results ... 7

Potential confounders ... 7

Causal associations between BMI and levels of inflammatory variables: two-sample MR ... 7

Supplemental Figures ... 8

Figure S1. Comparison of 27 SNP-cytokine association estimates with and without adjusting for BMI. ... 8

Figure S2. Observational associations of body mass index (BMI) and inflammation related variables by cohort and overall ... 9

Figure S3. Observational associations of body mass index (BMI) and inflammation related variables by cohort and overall adjusted for potential confounders ... 10

Figure S4. Mendelian randomization associations of body mass index (BMI) and inflammation related variables by cohort and overall. ... 11

Figure S5. Mendelian randomization associations of body mass index (BMI) and inflammation related variables using a two-sample approach. ... 12

Figure S6. Mendelian randomization associations of body mass index (BMI) and four inflammation related variables (CRP, HGF, MCP-1, TRAIL) using a two-sample approach. ... 13

Figure S7. Forest plots of SNP-specific Mendelian randomization associations between circulating TRAIL and odds of coronary artery disease. ... 14

Supplemental Tables ... 15

Table S1. List of measured cytokines ... 15

Table S2. List of disease outcomes used in two-sample Mendelian randomization between BMI-driven cytokines and disease outcomes ... 17

Table S3. Cytokine summary statistics ... 18

Table S4. Association between body mass index (BMI) and confounders in the cohorts ... 20

Table S5. Association between cytokines and smoking in the cohorts 21
Table S6. Association between cytokines and alcohol consumption in the cohorts 23
Table S7. Association between cytokines and socioeconomic status in the cohorts 25
Table S8. List of 97 genetic variants associated with body mass index (BMI) 28
Table S9. Association of body mass index (BMI) GRS with measured BMI and potential confounders 32
Table S10. First stage F-statistics and Durbin-Wu-Hausman statistics from one-sample Mendelian randomization analysis 33
Table S11. Results from one-sample Mendelian randomization analysis using natural log-transformed cytokine concentrations for a subset of cytokines that showed evidence of association with body mass index (BMI). 35
Table S12. Summary of SNPs used as instruments in the analysis of BMI-associated inflammation related variables and disease outcomes. 36
Table S13. Complete list of CRP instruments – see excel file 38
Table S14. Complete list of HGF instruments – see excel file 38
Table S15. Complete list of MCP-1 instruments – see excel file 38
Table S16. Complete list of TRAIL instruments – see excel file 38
Table S17. Association between rs12075 and circulating MCP-1: cohort-specific results and meta-analysis results with and without FINRISK 2002 39
Table S18. Results from the two-sample MR analysis between BMI-driven inflammation related variables and disease outcomes 40
Table S19. A) Gender specific SNP-inflammatory variable associations in each cohort for SNPs used as instruments for ovarian and breast cancer 43
Table S19. B) Meta-analysed gender specific SNP-inflammatory variable associations for SNPs used as instruments for ovarian and breast cancer 46
References 47

Supplemental Methods

Cohort descriptions

The Cardiovascular Risk in Young Finns Study

The Cardiovascular Risk in Young Finns Study (YFS) is a multicentre follow-up study with randomly chosen subjects from the Finnish cities of Helsinki, Kuopio, Oulu, Tampere, and Turku and their rural surroundings. The study began in 1980 when 3,596 children and young adults participated in the first cross-sectional survey. The follow-up visits have been conducted in 1983, 1986, 1989, 2001, 2007, and 2011. The present cross-sectional study includes 1980 unrelated individuals who participated in the 2007 follow-up and who had both cytokine measurements and genotype data available. All participants gave written informed consent and the study was approved by local ethics committees[1].

FINRISK

FINRISK surveys are population-based cross-sectional studies conducted every 5 years to monitor the levels of chronic disease risk factors in Finland. Each survey includes 25- to 74-year-old randomly chosen subjects from five geographical areas of Finland. The present study analyses cytokine data from participants of the 1997 and 2002 surveys. In FINRISK 2002 the cytokine panel was run for a subset of participants that were older than 51 years. The study visit includes a clinical examination and semi-fasting blood sampling. The study was approved by the Coordinating Ethics Committee of the Helsinki and Uusimaa Hospital District and all study participants gave written informed consent.

Genotypes

Genotyping of participants in YFS was carried out using the Illumina HumanHap 670K array and genotyping of participants in FINRISK 1997 and 2002 was carried out using Illumina HumanCoreExome, Illumina Omniexpress, Affymetrix 6.0 and Illumina 610K. In all three cohorts SNPs with violation of Hardy-Weinberg equilibrium (HWE $\leq 1 \times 10^{-6}$) were removed. SNPs with low call rate (<95%) and low minor allele frequency (MAF<1%) were also excluded before imputation. Imputation was performed using 1000 genomes phase 1 version 3 reference panel and IMPUTE2-software (version 2.1.2 (YFS), 2.3.2 (FINRISK 1997 & 2002)) in all cohorts. Genetic principal components were calculated using PLNK 1.06 (YFS) and newer versions (FINRISK 1997 & 2002). Calculations were carried out using "—mds-plot" -command and linkage disequilibrium (LD) pruned SNP data.

Laboratory analyses

The cytokine quantification was performed from semi-fasting (minimum 4 hours fasting) EDTA plasma in FINRISK 1997, from semi-fasting heparin plasma in FINRISK 2002 and from fasting serum in YFS. In YFS and FINRISK 2002, a total of 48 cytokines were measured by using Bio-Rad's premixed Bio-Plex Pro

Human Cytokine 27-plex and 21-plex assays, as previously described[2–4]. In FINRISK 1997, a custom selected 20-cytokine multiplex assay was run. Cytokines were quantified for 2200, 2775 and 7906 individuals in YFS, FINRISK 2002 and FINRISK 1997. After merging cytokine, genotype and BMI data, final sample sizes used in the three cohorts were 1980, 1705 and 4608 individuals, respectively. Seven cytokines (out of the 48) were excluded from all subsequent analyses due to excessive missingness (>90%). This left a total of 41 measured cytokines in YFS and FINRISK 2002, 17 of which were also available in FINRISK 1997. For YFS, FINRISK 1997 and 2002, CRP was measured using high-sensitive turbidimetric immunoassay kit with an automated analyser.

Cytokine GWAS

As part of this work, a revised version of the previously published GWAS of 41 inflammatory cytokines[2], without fitting BMI as a covariate in the model, was generated. The re-run was conducted on the same data as used previously (the Finnish cohorts described above) and all other aspects of the analysis were as described previously[2]. Leaving BMI out of the model increased the sample size by 44 individuals (39 in YFS and 5 in FINRISK 2002) who were excluded from the original GWAS and our Step 1 analyses because of having missing data on BMI.

Potential confounders

Description: Socioeconomic status was assessed using participant's educational background. In FINRISK 1997 and 2002 educational status was defined based on total years of full-time education and divided into three categories (low, average, high) and in YFS based on highest obtained degree (comprehensive school, secondary non-academic education and academic education). Smoking status was assessed using two categories, current smokers vs. ex and never smokers in FINRISK 1997 and 2002 and daily smokers vs. occasional and non-smokers in YFS. Alcohol consumption was used as a continuous covariate (average grams per week). To obtain approximate normality alcohol consumption was natural log-transformed prior to all analyses.

Part 1A – supplementary analyses: Associations of BMI, cytokines and CRP with potential confounders were estimated using linear regression. For BMI the confounder associations were adjusted for age and sex whereas for cytokines no additional adjustments were made due to the transformation process applied before analyses. Confounders tested were smoking status, alcohol consumption and socioeconomic status. Associations were estimated separately for each potential confounder (used as the independent variable) and BMI, cytokines and CRP (as dependent variables). In addition, sensitivity analyses were conducted for the observational associations between cytokines and BMI in which potential confounders were fitted as additional covariables in the model.

Selection of genetic variants for cytokines

The genetic instruments for the BMI-associated cytokines taken forward to analysis step 2 were selected

based on the results of our re-run of the previously published GWAS of 41 inflammatory cytokines (n=8337 from 3 studies)[2]. To maximise the number of available instruments for each cytokine, a list of cytokine-associated SNPs ($p<5x10^{-8}$) was first cross-matched to the corresponding GWAS summary statistics for each disease outcome in turn. During this harmonization process, SNPs for which strand alignment was ambiguous (A/T or G/C variants with intermediate allele frequencies) were removed. In cases where the associated SNP was not present, substitute SNPs (i.e. those in linkage disequilibrium with the associated SNP) were used where $r^2>0.8$ (based on the European subset of 1000 Genomes). Then, taking the subset of SNPs that were present (or substituted) in both datasets, we performed linkage disequilibrium (LD) clumping using an $r^2<0.01$ threshold in order to generate an independent set of instruments. This procedure was implemented using the TwoSampleMR R package[5] (version 0.4.26).

Derivation of body mass index (BMI) genetic risk score (GRS)

A weighted genetic risk score (GRS) for BMI was generated for use in the one-sample MR analysis (analysis step 1B) based on the 97 SNPs selected as instruments for BMI. A list of BMI SNPs and weights (i.e. betas extracted from the published meta-GWAS[6]) that contributed to the BMI GRS instrument is given in **Table S8**. The sum of genotypes (coded 0,1,2) was multiplied by the strength of the effect of each SNP on BMI (in SD units) derived from the same BMI GWAS used to select the instruments[6]. The validity of the weighted GRS as a genetic instrument was assessed by examination of: i) first stage F-statistic which is a measure of the strength of the association between the instrument and exposure (calculated from the regression of BMI on the weighted GRS instrument in the first stage of the two stage least squares), and ii) associations of the GRS with potential confounding factors (smoking status, alcohol consumption and socioeconomic status).

Causal associations between BMI and levels of inflammatory variables: two-sample MR

For completeness, the causal associations between BMI and levels of inflammatory variables assessed in a one-sample framework in the primary analysis (as presented in the main text) were also evaluated using a two-sample framework. The genetic instruments used were the same as those selected for the one-sample MR described in the main text (**Table S8**). SNP-exposure associations were extracted from the same BMI GWAS as used for instrument selection[6] and SNP-outcome associations from our re-run of a GWAS of 41 inflammatory cytokines (n=8337) and results from a recent meta-GWAS of CRP (n>200 000); rs2245368 was not present in the CRP dataset leaving 96 SNPs as instruments [7]. For TNF-β four SNPs (rs16851483, rs17024393, rs13107325, rs11847697) were missing from the 97 BMI-related instruments. They were excluded from the cytokine meta-analysis because of low minor allele count in one of the two cohorts for which TNF-β was available and only SNPs for which results from more than one cohort was available, were included in the final summary statistics and used in the analyses.

For each instrument (SNP), causal estimates were derived using the Wald ratio method and estimates combined using the inverse-variance weighted (IVW) method to provide a single causal estimate of the

exposure-outcome association[8]. To assess the potential for an effect of pleiotropic SNPs on the results, we performed the following sensitivity analyses: 1) MR-Egger regression method[9] to test overall directional pleiotropy and provide a valid causal estimate, taking into account the presence of pleiotropy; and 2) weighted median method[9] which provides a consistent estimate of causal effect if at least 50% of the information in the analysis comes from variants that are valid instrumental variables.

Power calculations

Retrospective power analyses were conducted to aid interpretation of null results. For the first step MR, power calculations were conducted using the online calculator available at (https://shiny.cnsgenomics.com/mRnd/)[10]. The parameters were as follows: alpha = 0.05, $beta_{OLS}$ = 0.08 (this was the median effect size seen in the observational analysis where $p<0.05$), R^2_{xz} = 0.027 (the variance in BMI explained by the GRS), variance of the exposure (X) = 1 and variance of the outcome (Y) = 1. The $beta_{yx}$ parameter that gave power = 80% was then determined for the two relevant sample sizes (N=8,000 and N=3,500). For the second step MR, power calculations were calculated using the following formula[11]:

$$Power = (pnorm(sqrt(N*R^2_{xz}*(ratio/(1+ratio))*(1/(1+ratio)))*beta_{yx}-qnorm(1-alpha/2)))*100$$

The parameters were as follows: sample size (N) = 50,000, ratio= 1:2 (ratio of cases to controls), alpha = 0.05 and R^2_{xz} (variance in trait explained by genetic variants) = 0.02, 0.05 and 0.025 for HGF, MCP1 and TRAIL, respectively. The $beta_{yx}$ parameter that gave 80% power was then determined given N=50,000 (our threshold for inclusion) and assuming a 1:2 ratio of cases to controls.

Supplemental Results

Potential confounders

In an investigation of the potential confounders of the observational relationship between BMI and the inflammation related variables, smoking and higher socioeconomic status were associated with lower BMI, with between group BMI shifts of -0.069 SD (95% CI: -0.12,-0.018, p=0.008) and -0.27 SD (95% CI: -0.33, -0.22, p=9.79x10^{-22}), respectively (**Table S4**). Smoking status and 13 measured cytokines (bNGF, Eotaxin, HGF, IL-18, IL-2ra, IL-6, IL-8, IP10, MCP-1, MIG, SCF, SCGFβ and VEGF) as well as CRP, were associated in a meta-analysis of observational associations (**Table S5**). Associations were also seen between both alcohol consumption (**Table S6**) and socioeconomic status (**Table S7**) and a number of cytokines.

Causal associations between BMI and levels of inflammatory variables: two-sample MR

Causal effect estimates of BMI on the 41 cytokine measures estimated from two-sample MR are shown in **Figure S5**. In line with the one-sample results presented in the main text, there was evidence of a causal effect of BMI on CRP and three cytokines: HGF, MCP-1 and TRAIL (p<0.001) with suggestive evidence for association with a further seven cytokines (p<0.05). There was no evidence of heterogeneity across the 97 BMI-associated variants (Q-statistic p>0.05) for HGF, MCP-1 and TRAIL (**Figure S6**).

Using the 96 available SNPs as instruments, a one SD increase in BMI was associated with a 0.35 (95%CI: 0.24, 0.46; p<1.61x10^{-10}) increase in natural log-transformed CRP levels. Including the 93 available SNPs for HGF, a one SD increase in BMI was associated with a 0.32 normalized SD (95% CI: 0.17, 0.46; p<1.25x10^{-5}) increase in HGF. Using all 97 BMI-associated SNPs, a one SD increase in BMI was associated with a 0.26 (95% CI: 0.13, 0.40; p<0.0001) and 0.24 (95% CI: 0.11, 0.37; p<0.0004) normalized SD increase in MCP-1 and TRAIL, respectively. Results from the IVW analyses were consistent with the weighted median and MR Egger analyses for all four associated inflammatory variables (**Figures S5 and S6**)

Supplemental Figures

Figure S1. Comparison of 27 SNP-cytokine association estimates with and without adjusting for BMI.

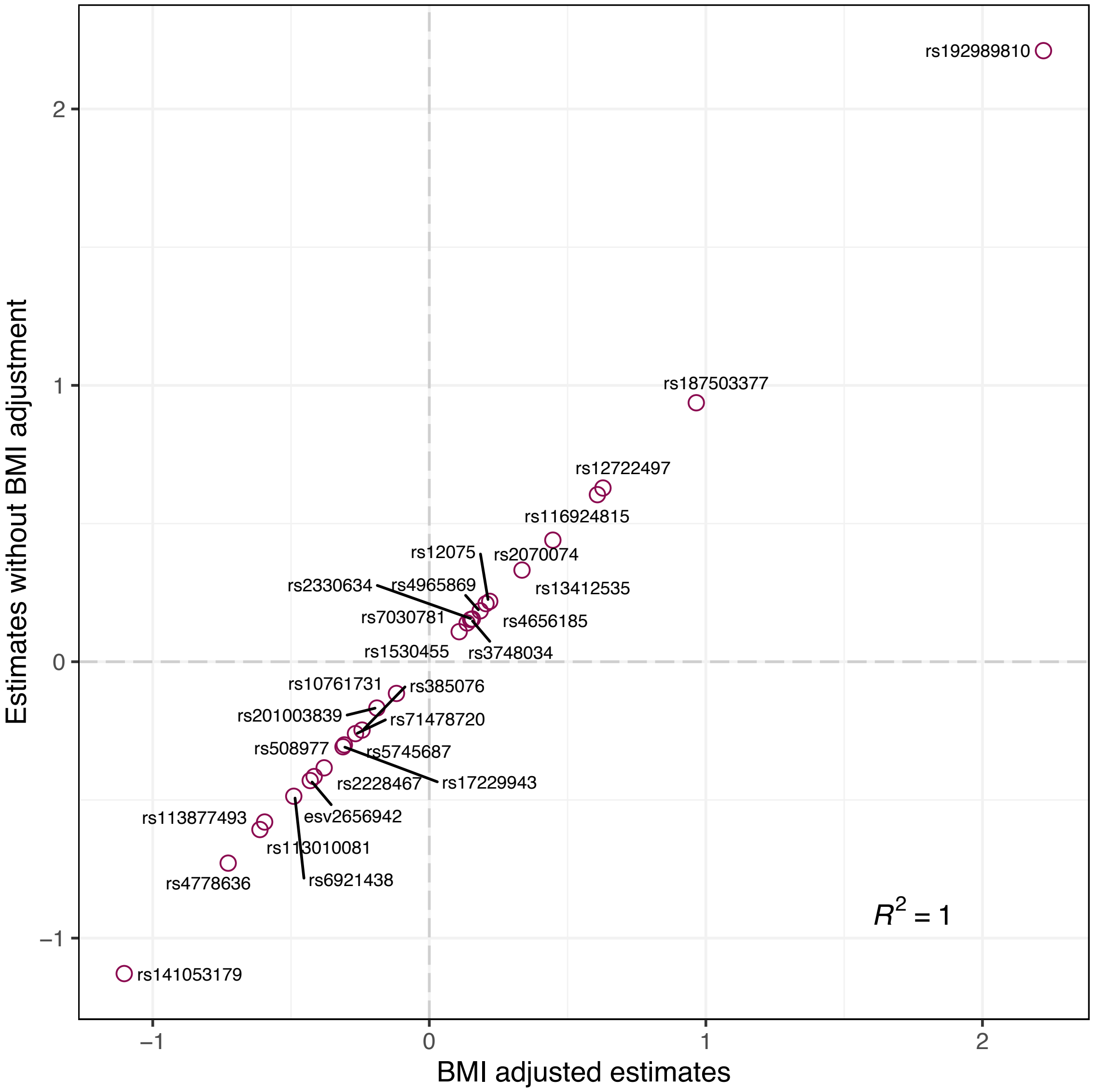

BMI adjusted SNP-cytokine associations were obtained from previously published GWAS by Ahola-Olli *et al.* (2). Corresponding SNP-cytokine estimates were extracted from the re-run of the same GWAS without adjusting for BMI.

Figure S2. Observational associations of body mass index (BMI) and inflammation related variables by cohort and overall.

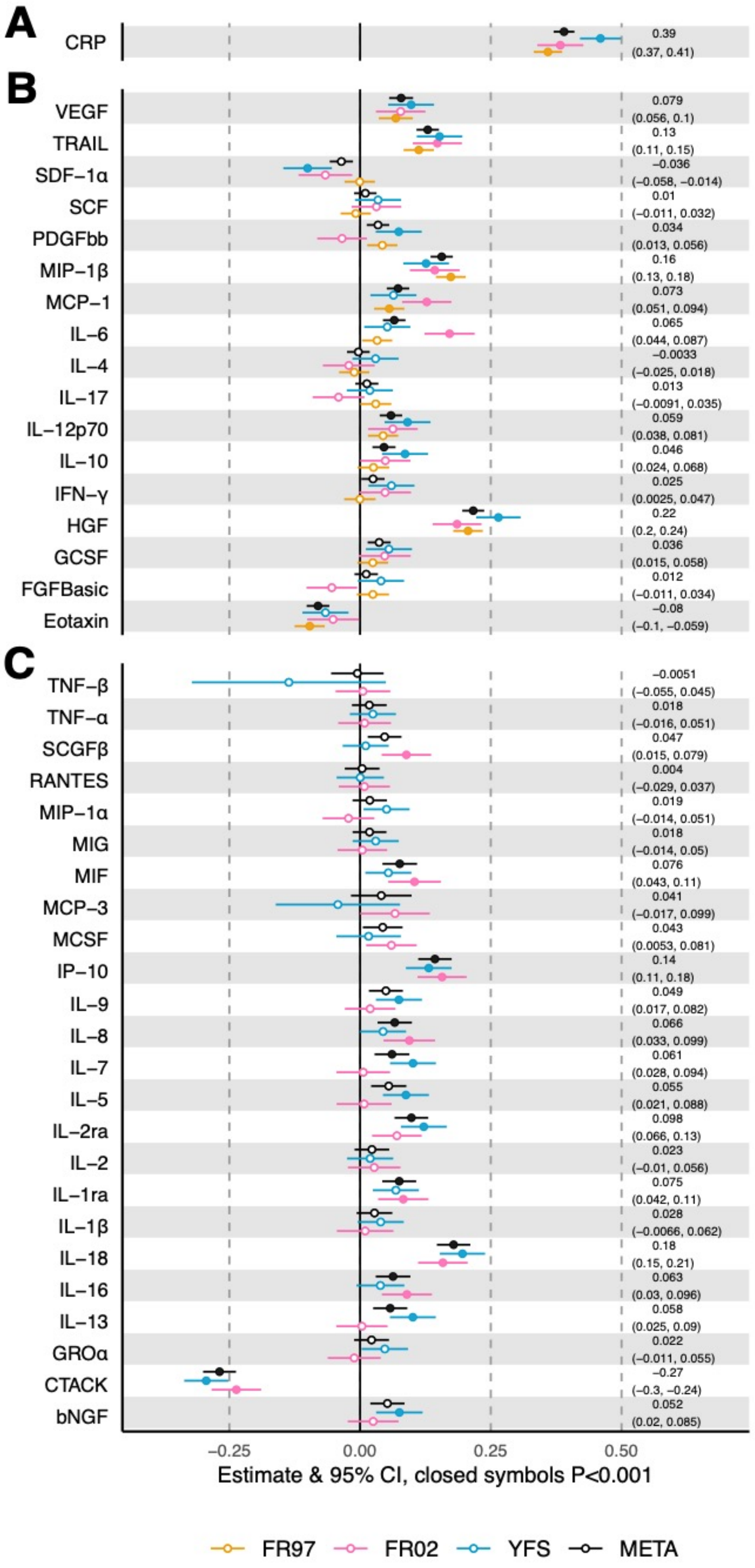

Effect estimates are given in normalized SD units per 1-SD higher BMI. (A) C-reactive protein (CRP) result (YFS, FINRISK 1997, FINRISK 2002); (B) Results for cytokines available in all three cohorts (YFS, FINRISK 1997, FINRISK 2002); (C) Results for cytokines available only in two cohorts (YFS, FINRISK 2002).

Figure S3. Observational associations of body mass index (BMI) and inflammation related variables by cohort and overall adjusted for potential confounders

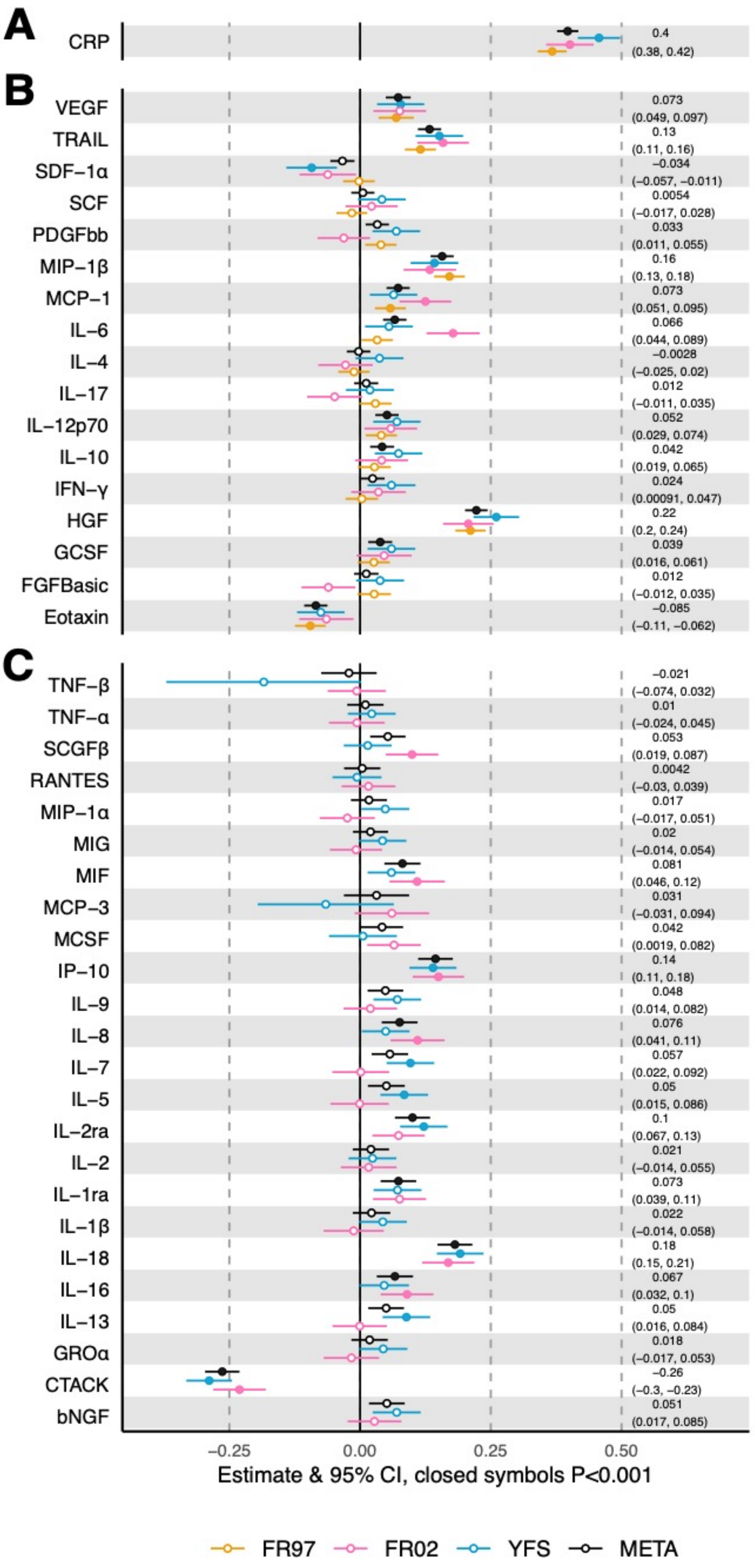

Effect estimates are given in normalized SD units per 1-SD higher BMI with adjustment for smoking status, alcohol consumption and socioeconomic status. (A) C-reactive protein (CRP) result (YFS, FINRISK 1997, FINRISK 2002); (B) Results for cytokines available in all three cohorts (YFS, FINRISK 1997, FINRISK 2002); (C) Results for cytokines available only in two cohorts (YFS, FINRISK 2002).

Figure S4. Mendelian randomization associations of body mass index (BMI) and inflammation related variables by cohort and overall.

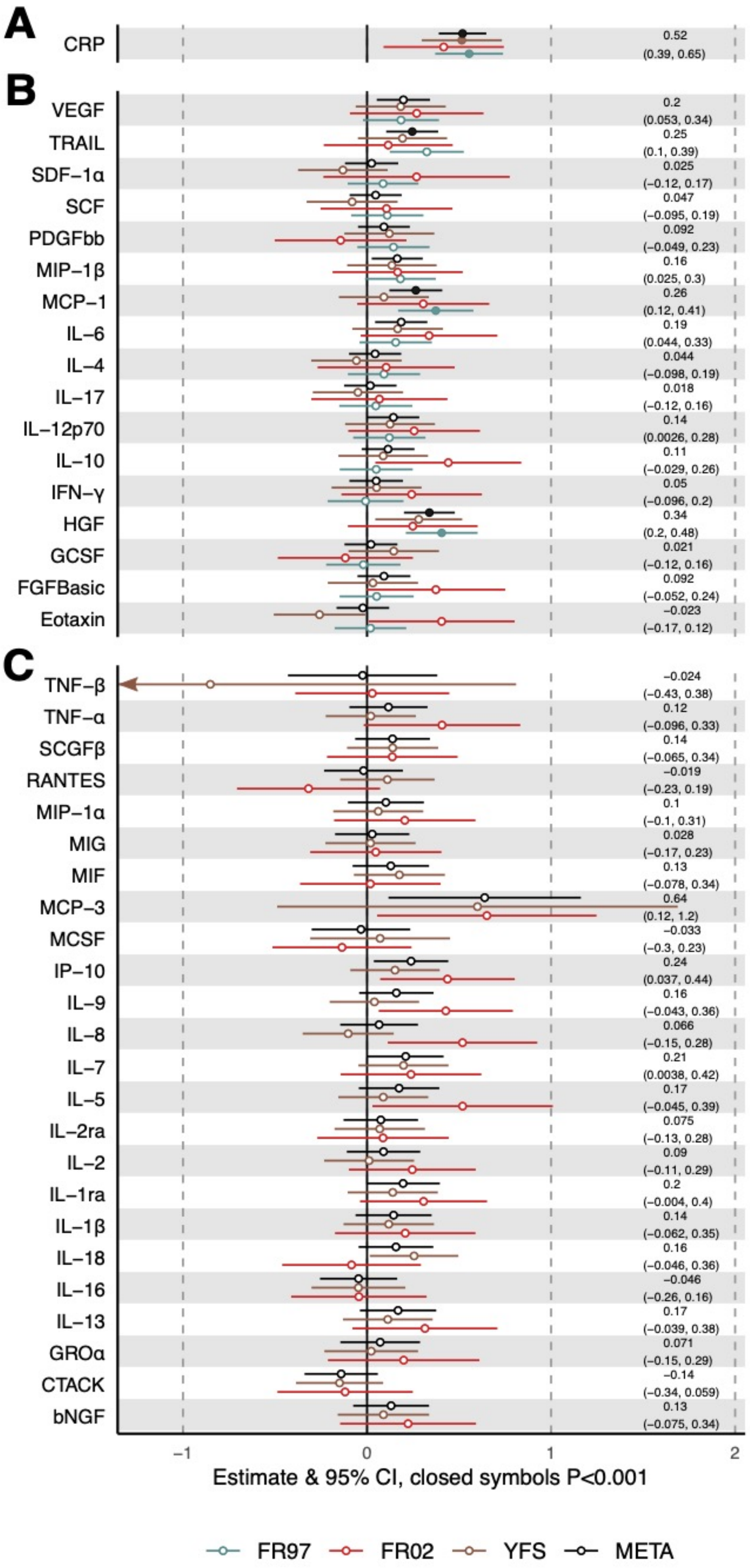

Effect estimates are given in normalized SD units per 1-SD higher BMI. (A) C-reactive protein (CRP) result (YFS, FINRISK 1997, FINRISK 2002); (B) Results for cytokines available in all three cohorts (YFS, FINRISK 1997, FINRISK 2002); (C) Results for cytokines available only in two cohorts (YFS, FINRISK 2002).

Figure S5. Mendelian randomization associations of body mass index (BMI) and inflammation related variables using a two-sample approach.

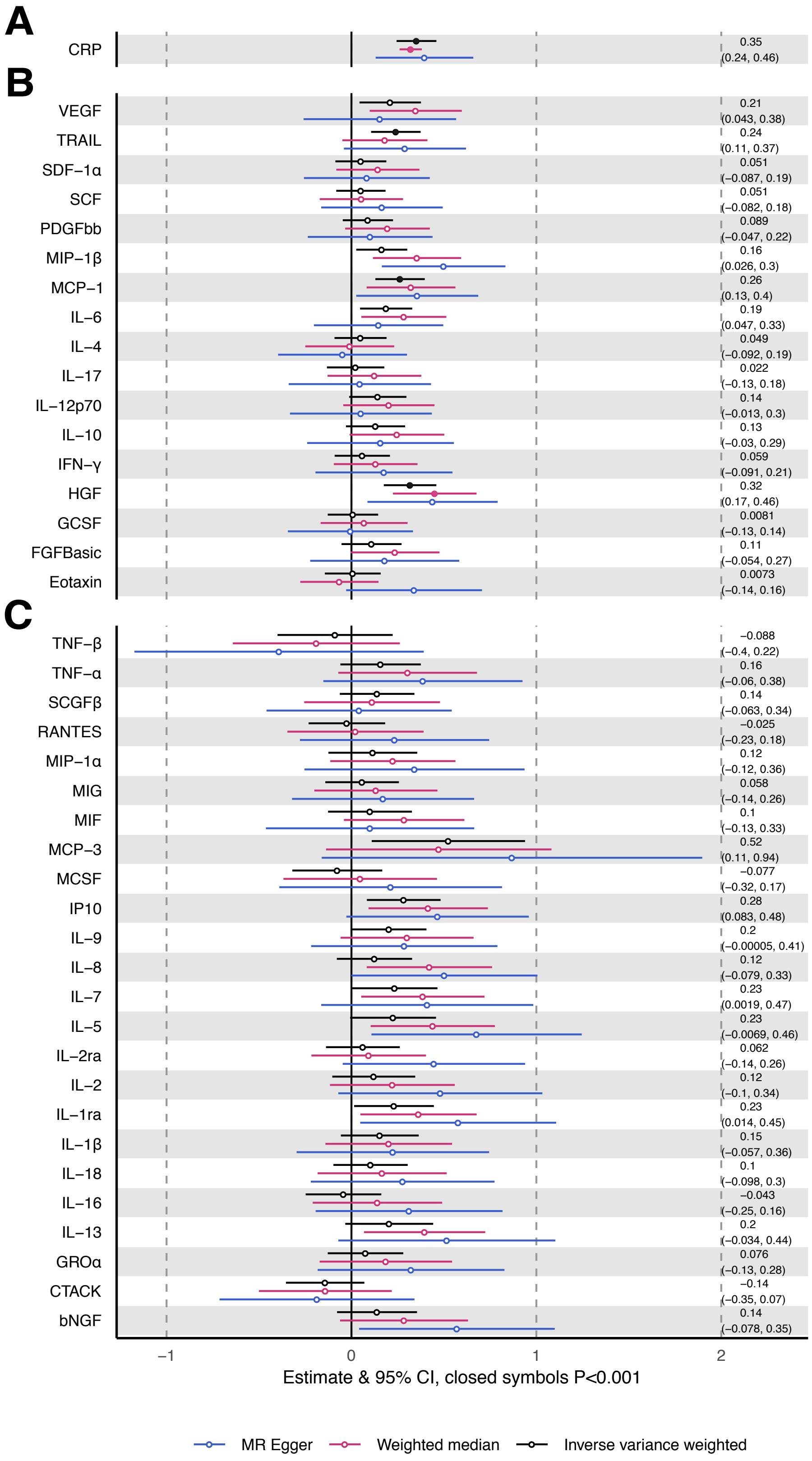

Effect estimates are given in normalized SD units per 1-SD higher BMI. Estimates derived from three alternative methods are presented. (A) C-reactive protein (CRP) result (YFS, FINRISK 1997, FINRISK 2002); (B) Results for cytokines available in all three cohorts (YFS, FINRISK 1997, FINRISK 2002); (C) Results for cytokines available only in two cohorts (YFS, FINRISK 2002).

Figure S6. Mendelian randomization associations of body mass index (BMI) and four inflammation related variables (CRP, HGF, MCP-1, TRAIL) using a two-sample approach.

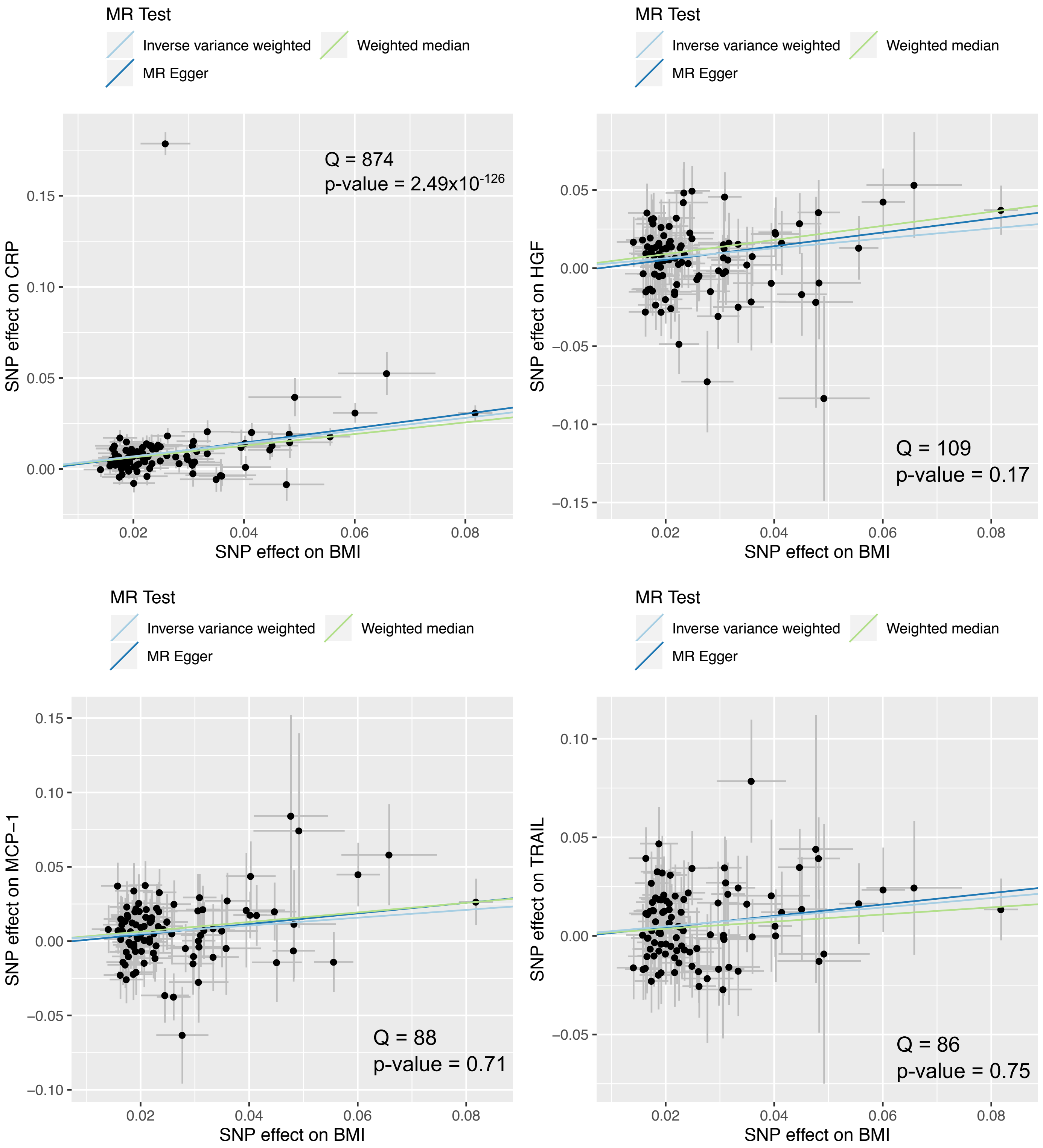

Effect estimates are given in normalized SD units per 1-SD higher BMI. Estimates derived from three alternative methods are presented. Q refers to the Cochran's Q heterogeneity statistic and p-value to the corresponding p-value.

Figure S7. Forest plots of SNP-specific Mendelian randomization associations between circulating TRAIL and odds of coronary artery disease.

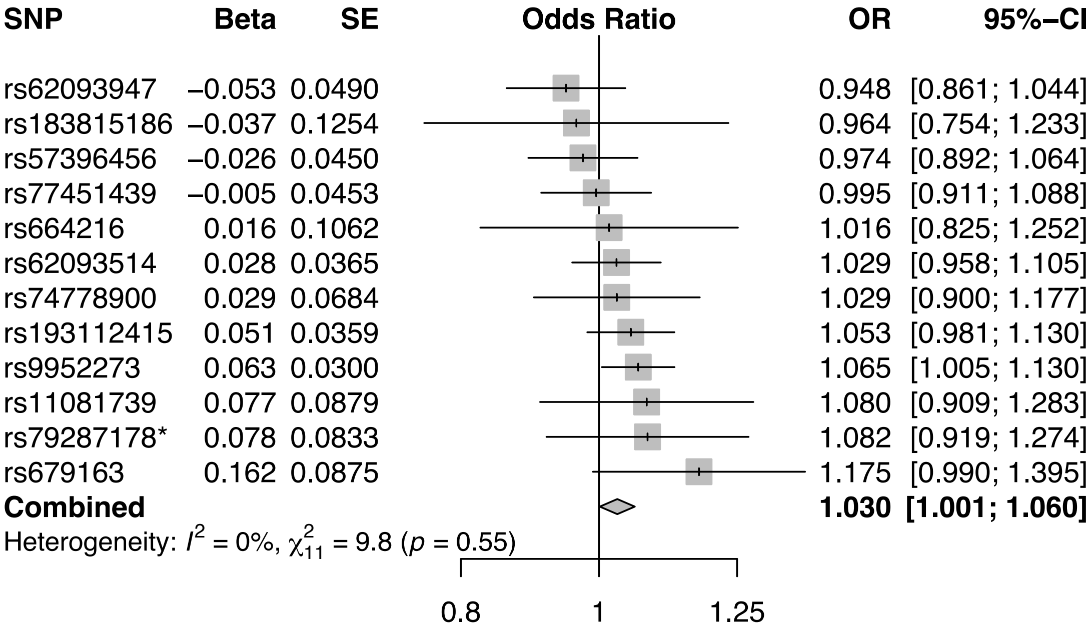

Estimates correspond to the odds ratio for coronary artery disease (CARDIoGRAMplusC4D, Ncases=60801, Ncontrols=123504) per normalized SD increase in circulating TRAIL (N=8230) levels.
*cis variant for TRAIL
OR, odds ratio; CI, confidence interval; SNP, single nucleotide polymorphism.

Supplemental Tables

Table S1. List of measured cytokines

Full name	Systematic nomenclature	Name	Assay
Beta nerve growth factor		bNGF	21-plex assay
Cutaneous T-cell attracting	CCL27	CTACK	21-plex assay
Eotaxin	CCL11	Eotaxin	27-plex assay & custom-made multiplex assay
Basic fibroblast growth factor		FGFBasic	27-plex assay & custom-made multiplex assay
Granulocyte colony-stimulating factor		GCSF	27-plex assay & custom-made multiplex assay
Growth regulated oncogene-α	CXCL1	GROα	21-plex assay
Hepatocyte growth factor		HGF	21-plex assay & custom-made multiplex assay
Interferon-gamma		IFN-γ	27-plex assay & custom-made multiplex assay
Interleukin-10		IL-10	27-plex assay & custom-made multiplex assay
Interleukin-12p70		IL-12p70	27-plex assay & custom-made multiplex assay
Interleukin-13		IL-13	27-plex assay
Interleukin-16		IL-16	21-plex assay
Interleukin-17		IL-17	27-plex assay & custom-made multiplex assay
Interleukin-18		IL-18	21-plex assay
Interleukin-1-beta		IL-1β	27-plex assay
Interleukin-1 receptor antagonist		IL-1ra	27-plex assay
Interleukin-2		IL-2	27-plex assay
Interleukin-2 receptor, alpha subunit		IL-2ra	21-plex assay
Interleukin-4		IL-4	27-plex assay & custom-made multiplex assay
Interleukin-5		IL-5	27-plex assay
Interleukin-6		IL-6	27-plex assay & custom-made multiplex assay
Interleukin-7		IL-7	27-plex assay
Interleukin-8	CXCL8	IL-8	27-plex assay
Interleukin-9		IL-9	27-plex assay
Interferon gamma-induced protein 10	CXCL10	IP10	27-plex assay
Monocyte chemotactic protein-1	CCL2	MCP-1	27-plex assay & custom-made multiplex assay
Monocyte specific chemokine 3	CCL7	MCP-3	21-plex assay
Macrophage colony-stimulating factor		MCSF	21-plex assay
Macrophage migration inhibitory factor (glycosylation-inhibiting factor)		MIF	21-plex assay
Monokine induced by interferon-gamma	CXCL9	MIG	21-plex assay
Macrophage inflammatory protein-1α	CCL3	MIP-1α	27-plex assay
Macrophage inflammatory protein-1β	CCL4	MIP-1β	27-plex assay & custom-made multiplex assay
Platelet derived growth factor BB		PDGFbb	27-plex assay & custom-made multiplex assay
Regulated on Activation, Normal T Cell Expressed and Secreted	CCL5	RANTES	27-plex assay (& custom-made multiplex assay[a])
Stem cell factor		SCF	21-plex assay & custom-made multiplex assay
Stem cell growth factor beta		SCGFβ	21-plex assay
Stromal cell-derived factor-1 alpha	CXCL12	SDF-1α	21-plex assay & custom-made multiplex assay
Tumor necrosis factor-alpha		TNF-α	27-plex assay

Tumor necrosis factor-beta		TNF-β	21-plex assay (& custom-made multiplex assay[a])
TNF-related apoptosis inducing ligand		TRAIL	21-plex assay & custom-made multiplex assay
Vascular endothelial growth factor		VEGF	27-plex assay & custom-made multiplex assay
**Cytokines excluded from the analyses[b]**			
Granulocyte-macrophage colony-stimulating factor		GMCSF	27-plex assay & custom-made multiplex assay
Interferon alpha 2		IFN-α2	21-plex assay
Leukemia inhibitory factor		LIF	21-plex assay
Interleukin-1 alpha		IL-1α	21-plex assay
Interleukin-3		IL-3	21-plex assay
Interleukin-15		IL-15	27-plex assay
Interleukin-12p40		IL-12p40	21-plex assay

[a]Custom-made multiplex assay data not included in our analyses for these cytokines
[b]Cytokines with >90% of values missing were excluded

Table S2. List of disease outcomes used in two-sample Mendelian randomization between BMI-driven cytokines and disease outcomes.

Trait	N cases	N controls	Sample size	Population	Sex	N SNPs	Units	Author	Consortium	PMID (ref)	year	ID[a]
**Cardiovascular disease**												
Coronary artery disease	60801	123504	184305	Mixed	Males and females	9455779	log odds	Nikpay	CARDIoGRAMplusC4D	26343387 [12]	2015	7
Coronary artery disease	22233	64762	86995	European	Males and females	2420361	log odds	Schunkert H	CARDIoGRAM	21378990 [13]	2011	8
Myocardial infarction	43676	128199	171875	Mixed	Males and females	9289492	log odds	Nikpay	CARDIoGRAMplusC4D	26343387 [12]	2015	798
**Diabetes**												
Type 2 diabetes	26488	83964	110452	Mixed	Males and females	2915012	log odds	Mahajan A	DIAGRAM	24509480 [14]	2014	23
**Autoimmune / inflammatory**												
Crohn's disease (immunochip)	17897	33977	51874	European	Males and females	124888	log odds	Liu	IIBDGC	26192919 [15]	2015	12
Crohn's disease (GWAS)	5956	14927	20883	European	Males and females	12276506	log odds	Liu	IIBDGC	26192919 [15]	2015	30
Inflammatory bowel disease[b] (immunochip)	31665	33977	65642	European	Males and females	157116	log odds	Liu	IIBDGC	26192919 [15]	2015	294
Inflammatory bowel disease[b] (GWAS)	12882	21770	34652	European	Males and females	12716084	log odds	Liu	IIBDGC	26192919 [15]	2015	31
Ulcerative colitis (immunochip)	13768	33977	47745	European	Males and females	156116	log odds	Liu	IIBDGC	26192919 [15]	2015	970
Ulcerative colitis (GWAS)	6968	20464	27432	European	Males and females	12255197	log odds	Liu	IIBDGC	26192919 [15]	2015	32
Rheumatoid arthritis	19234	61565	80799	Mixed	Males and females	9739304	log odds	Okada Y	NA	24390342 [16]	2014	833
**Psychiatric / neurological disease**												
Alzheimer's disease	17008	37154	54162	European	Males and females	7055882	log odds	Lambert	IGAP	24162737 [17]	2013	297
Schizophrenia	35476	46839	82315	Mixed	Males and females	9444231	log odds	Ripke S	PGC	25056061 [18]	2014	22
**Cancer**												
Breast cancer	122977	105974	228951	European	Females	10680257	log odds	Michailidou K	BCAC	29059683 [19]	2017	NA
Ovarian cancer	25509	40941	66450	European	Females	NA	log odds	Phelan	OCAC	28346442 [20]	2017	1120

[a]Refers to id number in MR-Base/TwoRampleMR R-package (version 0.4.26). [b]Inflammatory bowel disease is a combination of both Crohn’s disease and Ulcerative colitis.

Table S3. Cytokine summary statistics

Cytokine	Total N	YOUNG FINNS STUDY			FINRISK 1997			FINRISK 2002		
		N	mean (sd)	median (IQR)	N	mean (sd)	median (IQR)	N	mean (sd)	median (IQR)
**bNGF (pg/ml)**	3531	1911	1.51 (1.16)	1.38 (0.71)	NA	NA	NA	1620	3.05 (30.52)	1.92 (0.87)
**CTACK (pg/ml)**	3631	1980	846.55 (243.77)	820.56 (309.88)	NA	NA	NA	1651	1973.61 (668.44)	1871.74 (825.13)
**Eotaxin (pg/ml)**	8153	1972	140.08 (205.11)	116.25 (58.5)	4599	74.52 (33.47)	68.45 (35.91)	1582	72.88 (227.33)	43.9 (38.35)
**FGFBasic (pg/ml)**	7565	1978	72.80 (36.61)	66.81 (21.81)	3963	32.04 (42.13)	23.73 (27.34)	1624	46.49 (82.99)	32.38 (22.95)
**GCSF (pg/ml)**	7904	1979	151.04 (355.01)	137.02 (40.18)	4381	149.50 (114.65)	127.06 (114.71)	1544	57.55 (147.12)	42.15 (24.7)
**GROα (pg/ml)**	3505	1964	91.13 (48.14)	83.77 (52.05)	NA	NA	NA	1541	50.20 (29.46)	47.02 (22.91)
**HGF (pg/ml)**	8292	1980	547.65 (206.60)	509.19 (241.59)	4608	354.36 (291.89)	324.44 (139.53)	1704	515.14 (917.24)	462.85 (192.59)
**IFN-γ (pg/ml)**	7701	1980	283.97 (201.78)	263.34 (84.15)	4175	101.52 (170.18)	82.02 (81.59)	1546	177.16 (1180.92)	46.86 (29.83)
**IL-10 (pg/ml)**	7681	1977	28.46 (127.78)	18.98 (11.58)	4049	2.73 (8.15)	1.89 (2.42)	1655	20.77 (159.06)	5.26 (4.35)
**IL-12p70 (pg/ml)**	8270	1980	90.49 (273.81)	66.82 (44.36)	4592	27.53 (135.67)	19 (19.37)	1698	51.88 (294.21)	19.75 (16.55)
**IL-13 (pg/ml)**	3557	1980	21.72 (52.94)	17.52 (7.91)	NA	NA	NA	1577	15.68 (95.37)	5.47 (5.24)
**IL-16 (pg/ml)**	3483	1820	85.57 (63.79)	78.24 (55.61)	NA	NA	NA	1663	394.39 (265.99)	367.93 (222.56)
**IL-17 (pg/ml)**	7760	1980	283.33 (116.94)	266.82 (83.47)	4243	73.96 (75.10)	54.34 (66.56)	1537	67.96 (311.17)	34.56 (25.63)
**IL-18 (pg/ml)**	3636	1980	72.47 (38.11)	65.56 (36.22)	NA	NA	NA	1656	219.84 (106.00)	197.91 (106.78)
**IL-1β (pg/ml)**	3309	1979	5.12 (1.88)	4.83 (1.5)	NA	NA	NA	1330	3.42 (25.79)	0.86 (0.64)
**IL-1ra (pg/ml)**	3638	1980	643.68 (11610.59)	228.63 (86.2)	NA	NA	NA	1658	384.53 (3510.49)	55.61 (49.61)
**IL-2 (pg/ml)**	3475	1977	25.81 (98.24)	18.76 (5.67)	NA	NA	NA	1498	16.56 (122.20)	4.98 (4.65)
**IL-2ra (pg/ml)**	3677	1973	85.91 (58.06)	79.09 (44.73)	NA	NA	NA	1704	259.12 (152.44)	235.38 (121.7)
**IL-4 (pg/ml)**	8124	1980	11.46 (2.15)	11.44 (2.16)	4604	4.27 (4.54)	3.82 (2.58)	1540	2.74 (23.92)	1.03 (0.67)
**IL-5 (pg/ml)**	3364	1978	6.63 (11.89)	6.01 (1.98)	NA	NA	NA	1386	4.99 (40.42)	1.11 (1)
**IL-6 (pg/ml)**	8189	1979	15.35 (51.76)	11.77 (3.55)	4607	12.08 (35.24)	10.36 (6.53)	1603	17.84 (140.09)	5.67 (5.1)
**IL-7 (pg/ml)**	3409	1980	23.98 (42.10)	20.18 (8.18)	NA	NA	NA	1429	29.79 (177.02)	7.61 (11.01)
**IL-8 (pg/ml)**	3526	1980	32.65 (9.15)	31.63 (7.34)	NA	NA	NA	1546	13.68 (18.78)	10.66 (4.8)
**IL-9 (pg/ml)**	3634	1978	338.70 (5349.92)	55.97 (26.43)	NA	NA	NA	1656	299.22 (2309.90)	22.34 (28.41)
**IP10 (pg/ml)**	3685	1980	722.29 (545.43)	598.24 (382.77)	NA	NA	NA	1705	596.03 (516.09)	473.46 (348.64)
**MCP-1 (pg/ml)**	8293	1980	35.63 (21.69)	2.48 (3.5)	4608	27.39 (22.25)	25.19 (10.23)	1705	91.13 (56.72)	23.64 (15.2)
**MCP-3 (pg/ml)**	1085	242	26.70 (71.82)	32.7 (13.99)	NA	NA	NA	843	48.60 (346.72)	84.32 (35.33)
**MCSF (pg/ml)**	2472	840	4.31 (11.29)	9.03 (21.46)	NA	NA	NA	1632	27.75 (33.75)	15.58 (21.48)
**MIF (pg/ml)**	3494	1978	181.07 (139.64)	158.6 (113.95)	NA	NA	NA	1516	705.95 (657.07)	535.43 (466.95)
**MIG (pg/ml)**	3685	1980	575.64 (538.06)	443.06 (270.62)	NA	NA	NA	1705	2019.54 (2157.14)	1543 (1115.91)

Cytokine	Total N	YOUNG FINNS STUDY			FINRISK 1997			FINRISK 2002		
		N	mean (sd)	median (IQR)	N	mean (sd)	median (IQR)	N	mean (sd)	median (IQR)
**MIP-1α (pg/ml)**	3522	1980	13.04 (4.06)	12.25 (3.09)	NA	NA	NA	1542	6.92 (8.93)	5 (3.45)
**MIP-1β (pg/ml)**	8243	1980	91.46 (72.21)	85.61 (36.09)	4608	57.42 (37.17)	52.13 (24.56)	1655	72.11 (40.30)	64.12 (27.28)
**PDGFbb (pg/ml)**	8293	1980	8889.65 (3104.06)	8521.95 (3642.62)	4608	1247.87 (1200.1)	906.67 (1179.27)	1705	507.05 (343.44)	448.32 (310.9)
**RANTES (pg/ml)**	3421	1836	17155.92 (38635.14)	11079.83 (7293.85)	NA	NA	NA	1585	4520.49 (5048.97)	3082.99 (2859.37)
**SCF (pg/ml)**	8290	1979	97.44 (196.98)	90.75 (35.31)	4608	115.83 (229.57)	108.43 (37.72)	1703	281.99 (114.68)	272.99 (103.66)
**SCGFβ (pg/ml)**	3682	1978	11690.45 (4825.92)	10986.54 (5594.12)	NA	NA	NA	1704	51129.73 (18850.63)	48284.43 (23123.41)
**SDF-1α (pg/ml)**	7787	1789	72.77 (34.45)	69.61 (33.74)	4514	125.95 (96.95)	113.82 (86.85)	1484	89.37 (101.46)	81.19 (39.42)
**TNF-α (pg/ml)**	3454	1980	62.74 (203.55)	48.79 (16.83)	NA	NA	NA	1474	67.64 (598.82)	9.17 (9.9)
**TNF-β (pg/ml)**	1559	109	19.30 (55.49)	3.34 (9.96)	NA	NA	NA	1450	15.87 (14.51)	13.93 (10.15)
**TRAIL (pg/ml)**	8186	1973	151.38 (120.81)	133.19 (75.49)	4513	114.33 (120.75)	94.51 (66.91)	1700	196.80 (158.63)	181.22 (83.13)
**VEGF (pg/ml)**	7118	1980	82.29 (47.34)	71.31 (56.35)	3435	23.38 (30.78)	14.99 (23.65)	1703	46.67 (76.14)	35.88 (22.72)

sd, standard deviation; IQR, interquartile range

Table S4. Association between body mass index (BMI) and confounders in the cohorts

	Young Finns Study			FINRISK 1997			FINRISK 2002			Meta-analysis		
	Beta	(95% CI)	P-value	Beta	(95% CI)	P-value	Beta	(95% CI)	P-value	Beta	(95% CI)	P-value
**Smoking**	0.030	(-0.081, 0.14)	0.59	-0.059	(-0.12, 0.0056)	0.073	-0.24	(-0.36, -0.11)	0.00027	-0.069	(-0.12, -0.018)	**0.0080**
**Alcohol consumption**	0.023	(0.0004, 0.045)	0.046	-0.018	(-0.034, -0.0015)	0.032	-0.026	(-0.054, 0.0016)	0.065	-0.0077	(-0.020, 0.0042)	0.20
**Socioeconomic status**												
Average	0.11	(-0.093, 0.31)	0.30	-0.12	(-0.19, -0.047)	0.0011	-0.064	(-0.19, 0.057)	0.30	-0.086	(-0.14, -0.028)	**0.0036**
High	-0.27	(-0.48, -0.059)	0.013	-0.27	(-0.33, -0.20)	1.12E-14	-0.30	(-0.42, -0.18)	4.36E-07	-0.27	(-0.33, -0.22)	**9.79E-22**

BMI was used as a dependent variable and association with each confounder (as independent variable) was analysed separately using linear model. All models are adjusted for age and sex. Low socioeconomic status was used as a reference level in the association between BMI and socioeconomic status. Non-smoking status (ex, never and occasional smokers) was used as the reference for the association between BMI and smoking; Alcohol consumption was measured in grams per week and natural log-transformed for the analyses (one was added to zero values). Betas reflect change in BMI in SD units. CI, confidence interval.

Table S5. Association between cytokines and smoking in the cohorts

Smoking	Young Finns Study			FINRISK 1997			FINRISK 2002			Meta-analysis		
Cytokine	Beta	(95% CI)	P-value	Beta	(95% CI)	P-value	Beta	(95% CI)	P-value	Beta	(95% CI)	P-value
bNGF	-0.28	(-0.40, -0.17)	2.10E-06	NA	NA	NA	-0.026	(-0.15, 0.10)	0.69	-0.16	(-0.25, -0.079)	**0.00016**
CRP	0.14	(0.025, 0.25)	0.017	0.18	(0.11, 0.24)	2.00E-07	0.35	(0.23, 0.48)	1.50E-08	0.2	(0.15, 0.25)	**3.40E-14**
CTACK	-0.11	(-0.23, 0.00071)	0.051	NA	NA	NA	0.013	(-0.11, 0.14)	0.84	-0.056	(-0.14, 0.028)	0.19
Eotaxin	0.21	(0.10, 0.33)	0.00024	0.26	(0.20, 0.33)	1.40E-14	0.14	(0.0096, 0.26)	0.035	0.23	(0.18, 0.28)	**6.00E-18**
FGFBasic	0.08	(-0.033, 0.19)	0.17	0.069	(-0.0029, 0.14)	0.06	0.026	(-0.10, 0.15)	0.69	0.063	(0.0087, 0.12)	0.023
GCSF	0.12	(0.0044, 0.23)	0.042	0.044	(-0.025, 0.11)	0.21	0.042	(-0.087, 0.17)	0.53	0.06	(0.0066, 0.11)	0.028
GROα	-0.12	(-0.23, -0.00082)	0.048	NA	NA	NA	-0.099	(-0.23, 0.031)	0.14	-0.11	(-0.19, -0.022)	0.014
HGF	0.42	(0.31, 0.53)	2.50E-13	0.27	(0.20, 0.34)	1.60E-15	0.35	(0.23, 0.47)	1.70E-08	0.32	(0.27, 0.37)	**2.40E-33**
IFN-γ	0.033	(-0.081, 0.15)	0.57	0.051	(-0.019, 0.12)	0.15	-0.016	(-0.14, 0.11)	0.81	0.035	(-0.019, 0.090)	0.2
IL-10	0.17	(0.052, 0.28)	0.0043	0.061	(-0.010, 0.13)	0.094	0.0034	(-0.12, 0.13)	0.96	0.074	(0.020, 0.13)	0.0076
IL-12p70	0.19	(0.081, 0.31)	0.00079	0.075	(0.0078, 0.14)	0.029	-0.0041	(-0.13, 0.12)	0.95	0.086	(0.034, 0.14)	0.0012
IL-13	0.2	(0.083, 0.31)	0.00071	NA	NA	NA	0.024	(-0.10, 0.15)	0.71	0.12	(0.035, 0.20)	0.0055
IL-16	0.11	(-0.0094, 0.23)	0.071	NA	NA	NA	-0.12	(-0.24, 0.0088)	0.068	0.0024	(-0.083, 0.088)	0.96
IL-17	0.026	(-0.087, 0.14)	0.65	0.067	(-0.0025, 0.14)	0.059	-0.036	(-0.17, 0.094)	0.59	0.04	(-0.014, 0.094)	0.15
IL-18	0.24	(0.12, 0.35)	4.80E-05	NA	NA	NA	0.069	(-0.056, 0.19)	0.28	0.16	(0.077, 0.24)	**0.00018**
IL-1β	0.024	(-0.090, 0.14)	0.68	NA	NA	NA	0.046	(-0.091, 0.18)	0.51	0.033	(-0.054, 0.12)	0.46
IL-1ra	0.039	(-0.075, 0.15)	0.5	NA	NA	NA	0.04	(-0.085, 0.16)	0.53	0.039	(-0.044, 0.12)	0.36
IL-2	0.00046	(-0.11, 0.11)	0.99	NA	NA	NA	0.025	(-0.11, 0.16)	0.71	0.011	(-0.075, 0.097)	0.8
IL-2ra	0.24	(0.13, 0.36)	3.10E-05	NA	NA	NA	0.13	(0.0082, 0.25)	0.037	0.19	(0.11, 0.27)	**7.30E-06**
IL-4	-0.025	(-0.14, 0.089)	0.67	0.11	(0.046, 0.18)	0.00093	0.068	(-0.062, 0.20)	0.31	0.076	(0.023, 0.13)	0.0047
IL-5	0.031	(-0.083, 0.14)	0.59	NA	NA	NA	-0.045	(-0.18, 0.092)	0.52	-0.00012	(-0.088, 0.087)	1
IL-6	0.032	(-0.082, 0.15)	0.58	0.12	(0.057, 0.19)	0.00027	0.24	(0.11, 0.37)	0.0002	0.12	(0.072, 0.18)	**3.10E-06**
IL-7	0.041	(-0.073, 0.15)	0.48	NA	NA	NA	0.019	(-0.11, 0.15)	0.78	0.032	(-0.055, 0.12)	0.47
IL-8	0.14	(0.026, 0.25)	0.016	NA	NA	NA	0.27	(0.14, 0.40)	4.00E-05	0.2	(0.11, 0.28)	**5.60E-06**
IL-9	0.071	(-0.043, 0.18)	0.22	NA	NA	NA	0.055	(-0.069, 0.18)	0.38	0.064	(-0.020, 0.15)	0.14
IP10	-0.37	(-0.48, -0.25)	2.40E-10	NA	NA	NA	-0.43	(-0.55, -0.30)	9.60E-12	-0.39	(-0.48, -0.31)	**1.00E-20**
MCSF	-0.15	(-0.32, 0.029)	0.1	NA	NA	NA	-0.11	(-0.24, 0.015)	0.084	-0.12	(-0.23, -0.021)	0.018
MCP-1	0.19	(0.078, 0.30)	0.00097	0.18	(0.11, 0.24)	1.90E-07	0.081	(-0.043, 0.20)	0.2	0.16	(0.11, 0.21)	**8.70E-10**
MCP-3	0.15	(-0.19, 0.48)	0.39	NA	NA	NA	-0.068	(-0.24, 0.11)	0.45	-0.021	(-0.18, 0.13)	0.79
MIF	0.057	(-0.057, 0.17)	0.33	NA	NA	NA	0.13	(-0.0017, 0.26)	0.053	0.088	(0.0021, 0.17)	0.045

Smoking	Young Finns Study			FINRISK 1997			FINRISK 2002			Meta-analysis		
Cytokine	Beta	(95% CI)	P-value	Beta	(95% CI)	P-value	Beta	(95% CI)	P-value	Beta	(95% CI)	P-value
MIG	-0.29	(-0.41, -0.18)	3.80E-07	NA	NA	NA	-0.13	(-0.25, -0.0068)	0.039	-0.22	(-0.30, -0.14)	**2.60E-07**
MIP-1α	0.069	(-0.044, 0.18)	0.23	NA	NA	NA	0.0035	(-0.13, 0.13)	0.96	0.041	(-0.045, 0.13)	0.35
MIP-1β	0.029	(-0.085, 0.14)	0.62	-0.076	(-0.14, -0.0090)	0.026	-0.14	(-0.26, -0.015)	0.028	-0.065	(-0.12, -0.013)	0.015
PDGFbb	0.098	(-0.015, 0.21)	0.09	0.057	(-0.010, 0.12)	0.097	0.0073	(-0.12, 0.13)	0.91	0.057	(0.0044, 0.11)	0.034
RANTES	0.0085	(-0.11, 0.13)	0.89	NA	NA	NA	0.12	(-0.0034, 0.25)	0.056	0.062	(-0.025, 0.15)	0.16
SCF	-0.15	(-0.27, -0.040)	0.0081	-0.08	(-0.15, -0.013)	0.019	-0.17	(-0.29, -0.042)	0.0084	-0.11	(-0.16, -0.059)	**3.20E-05**
SCGFβ	-0.16	(-0.27, -0.042)	0.0072	NA	NA	NA	-0.14	(-0.26, -0.013)	0.03	-0.15	(-0.23, -0.063)	**0.00057**
SDF-1α	-0.046	(-0.17, 0.074)	0.45	0.11	(0.047, 0.18)	0.00086	-0.017	(-0.15, 0.12)	0.8	0.061	(0.0072, 0.11)	0.026
TNF-α	-0.0012	(-0.11, 0.11)	0.98	NA	NA	NA	-0.065	(-0.20, 0.066)	0.33	-0.029	(-0.11, 0.057)	0.51
TNF-β	-0.39	(-0.94, 0.16)	0.16	NA	NA	NA	0.026	(-0.11, 0.16)	0.7	0.0026	(-0.13, 0.13)	0.97
TRAIL	0.095	(-0.020, 0.21)	0.1	0.046	(-0.021, 0.11)	0.18	0.021	(-0.10, 0.14)	0.74	0.052	(-0.00073, 0.10)	0.053
VEGF	0.22	(0.11, 0.33)	0.00015	0.12	(0.043, 0.20)	0.0022	0.12	(-0.0027, 0.24)	0.055	0.15	(0.089, 0.20)	**5.00E-07**

Rank-normal transformed cytokines were used as a dependent variable and smoking status was treated as independent variable. Analyses were run using linear regression without further adjustments. Non-smoking status (ex, never and occasional smokers) was used as reference level. Betas reflect change in cytokine levels in normalized SD units. CI, confidence interval.

Table S6. Association between cytokines and alcohol consumption in the cohorts

Alcohol consumption	Young Finns Study			FINRISK 1997			FINRISK 2002			Meta-analysis		
Cytokine	Beta	(95% CI)	P-value	Beta	(95% CI)	P-value	Beta	(95% CI)	P-value	Beta	(95% CI)	P-value
bNGF	0.0015	(-0.021, 0.024)	0.9	NA	NA	NA	-0.0023	(-0.029, 0.024)	0.86	-0.00011	(-0.017, 0.017)	0.99
CRP	0.015	(-0.0070, 0.037)	0.18	0.009	(-0.0067, 0.025)	0.26	0.013	(-0.012, 0.039)	0.3	0.012	(0.00010, 0.023)	0.048
CTACK	-0.0055	(-0.027, 0.017)	0.63	NA	NA	NA	0.034	(0.0076, 0.059)	0.011	0.011	(-0.0059, 0.028)	0.2
Eotaxin	0.014	(-0.0081, 0.036)	0.21	-0.02	(-0.035, -0.0040)	0.014	-0.0043	(-0.031, 0.022)	0.75	-0.0076	(-0.019, 0.0039)	0.2
FGFBasic	0.019	(-0.0031, 0.041)	0.092	-0.0091	(-0.026, 0.0078)	0.29	0.0055	(-0.021, 0.032)	0.68	0.0022	(-0.0097, 0.014)	0.72
GCSF	0.017	(-0.0050, 0.039)	0.13	-0.02	(-0.036, -0.0036)	0.016	0.0071	(-0.020, 0.034)	0.61	-0.0043	(-0.016, 0.0074)	0.47
GROα	0.00017	(-0.022, 0.022)	0.99	NA	NA	NA	-0.0064	(-0.033, 0.020)	0.64	-0.0025	(-0.020, 0.015)	0.77
HGF	0.0027	(-0.019, 0.025)	0.81	-0.0095	(-0.025, 0.0061)	0.23	0.0017	(-0.024, 0.027)	0.89	-0.004	(-0.015, 0.0074)	0.49
IFN-γ	0.0018	(-0.020, 0.024)	0.87	-0.014	(-0.030, 0.0028)	0.1	0.0047	(-0.022, 0.032)	0.73	-0.0056	(-0.017, 0.0062)	0.35
IL-10	0.029	(0.0075, 0.051)	0.0086	-0.013	(-0.029, 0.0041)	0.14	-0.028	(-0.054, -0.0022)	0.033	-0.0036	(-0.015, 0.0082)	0.55
IL-12p70	0.04	(0.018, 0.062)	0.00032	-0.022	(-0.038, -0.0065)	0.0056	-0.007	(-0.033, 0.019)	0.59	-0.0022	(-0.014, 0.0092)	0.71
IL-13	0.035	(0.013, 0.057)	0.0018	NA	NA	NA	-0.0032	(-0.030, 0.023)	0.81	0.019	(0.0025, 0.036)	0.024
IL-16	0.0043	(-0.019, 0.027)	0.71	NA	NA	NA	-0.035	(-0.061, -0.0093)	0.0077	-0.013	(-0.030, 0.0040)	0.13
IL-17	0.012	(-0.0097, 0.034)	0.27	-0.017	(-0.033, -0.00061)	0.042	-0.0061	(-0.033, 0.021)	0.66	-0.0064	(-0.018, 0.0053)	0.28
IL-18	0.0061	(-0.016, 0.028)	0.59	NA	NA	NA	-0.0014	(-0.027, 0.025)	0.91	0.003	(-0.014, 0.020)	0.73
IL-1β	2.60E-06	(-0.022, 0.022)	1	NA	NA	NA	0.017	(-0.012, 0.046)	0.25	0.0062	(-0.011, 0.024)	0.49
IL-1ra	-0.0016	(-0.024, 0.020)	0.89	NA	NA	NA	0.01	(-0.016, 0.036)	0.43	0.0034	(-0.013, 0.020)	0.69
IL-2	0.0034	(-0.019, 0.025)	0.76	NA	NA	NA	0.0081	(-0.019, 0.035)	0.56	0.0053	(-0.012, 0.022)	0.55
IL-2ra	0.018	(-0.0039, 0.040)	0.11	NA	NA	NA	-0.016	(-0.042, 0.0094)	0.21	0.0035	(-0.013, 0.020)	0.68
IL-4	0.011	(-0.011, 0.033)	0.32	-0.017	(-0.033, -0.0013)	0.033	0.0079	(-0.019, 0.035)	0.57	-0.0047	(-0.016, 0.0068)	0.42
IL-5	0.014	(-0.0081, 0.036)	0.22	NA	NA	NA	-0.0034	(-0.032, 0.025)	0.82	0.0074	(-0.010, 0.025)	0.4
IL-6	8.80E-05	(-0.022, 0.022)	0.99	-0.015	(-0.030, 0.0011)	0.069	0.0086	(-0.018, 0.035)	0.52	-0.0062	(-0.018, 0.0053)	0.29
IL-7	0.029	(0.0067, 0.051)	0.01	NA	NA	NA	-0.0055	(-0.034, 0.023)	0.7	0.016	(-0.0016, 0.033)	0.075
IL-8	0.0064	(-0.016, 0.028)	0.57	NA	NA	NA	0.019	(-0.0074, 0.046)	0.16	0.012	(-0.0053, 0.029)	0.18
IL-9	0.024	(0.0025, 0.046)	0.029	NA	NA	NA	-0.013	(-0.039, 0.013)	0.34	0.0091	(-0.0077, 0.026)	0.29
IP10	-0.0092	(-0.031, 0.013)	0.41	NA	NA	NA	-0.0086	(-0.034, 0.017)	0.51	-0.009	(-0.026, 0.0077)	0.29
MCSF	0.012	(-0.022, 0.045)	0.5	NA	NA	NA	-0.016	(-0.042, 0.011)	0.25	-0.0052	(-0.026, 0.016)	0.62
MCP-1	0.045	(0.023, 0.066)	6.40E-05	0.018	(0.0029, 0.034)	0.02	0.022	(-0.0037, 0.048)	0.093	0.026	(0.015, 0.038)	**5.90E-06**
MCP-3	0.015	(-0.052, 0.081)	0.67	NA	NA	NA	-0.025	(-0.061, 0.011)	0.18	-0.016	(-0.048, 0.016)	0.33

Alcohol consumption	Young Finns Study			FINRISK 1997			FINRISK 2002			Meta-analysis		
Cytokine	Beta	(95% CI)	P-value	Beta	(95% CI)	P-value	Beta	(95% CI)	P-value	Beta	(95% CI)	P-value
MIF	0.0022	(-0.020, 0.024)	0.85	NA	NA	NA	-0.034	(-0.061, -0.0068)	0.014	-0.012	(-0.029, 0.0049)	0.16
MIG	0.0014	(-0.021, 0.023)	0.9	NA	NA	NA	0.0091	(-0.016, 0.035)	0.49	0.0046	(-0.012, 0.021)	0.59
MIP-1α	0.0046	(-0.017, 0.027)	0.68	NA	NA	NA	-0.0039	(-0.031, 0.023)	0.78	0.0012	(-0.016, 0.018)	0.89
MIP-1β	-0.0017	(-0.024, 0.020)	0.88	-0.02	(-0.035, -0.0040)	0.014	0.0062	(-0.020, 0.032)	0.64	-0.0098	(-0.021, 0.0017)	0.094
PDGFbb	0.027	(0.0055, 0.049)	0.014	-0.022	(-0.037, -0.0059)	0.007	-0.014	(-0.039, 0.012)	0.3	-0.0067	(-0.018, 0.0047)	0.25
RANTES	0.019	(-0.0035, 0.042)	0.098	NA	NA	NA	-0.0086	(-0.035, 0.018)	0.53	0.0074	(-0.0098, 0.025)	0.4
SCF	-0.018	(-0.040, 0.0037)	0.1	-0.031	(-0.046, -0.015)	0.00011	-0.045	(-0.071, -0.020)	0.00048	-0.03	(-0.042, -0.019)	**1.70E-07**
SCGFβ	0.0094	(-0.013, 0.031)	0.4	NA	NA	NA	-0.014	(-0.040, 0.011)	0.27	-0.00069	(-0.017, 0.016)	0.94
SDF-1α	-0.011	(-0.034, 0.012)	0.36	-0.016	(-0.032, -0.00024)	0.047	-0.0046	(-0.032, 0.023)	0.74	-0.013	(-0.024, -0.00075)	0.037
TNF-α	0.0057	(-0.016, 0.028)	0.61	NA	NA	NA	-0.0075	(-0.035, 0.020)	0.59	0.00051	(-0.017, 0.018)	0.95
TNF-β	-0.023	(-0.12, 0.069)	0.62	NA	NA	NA	-0.0072	(-0.035, 0.021)	0.61	-0.0086	(-0.035, 0.018)	0.53
TRAIL	0.028	(0.0058, 0.050)	0.013	0.0095	(-0.0063, 0.025)	0.24	0.0087	(-0.017, 0.034)	0.5	0.014	(0.0028, 0.026)	0.015
VEGF	0.048	(0.026, 0.070)	1.90E-05	-0.017	(-0.035, 0.00054)	0.057	-0.018	(-0.044, 0.0075)	0.16	0.0028	(-0.0094, 0.015)	0.65

Rank-normal transformed cytokines were used as a dependent variable and alcohol consumption was treated as independent variable. Analyses were run using linear regression without further adjustments. Alcohol consumption was measured in average grams per week and natural log-transformed for the models (one was added to zero values). Betas reflect change in cytokine levels in normalized SD units. CI, confidence interval

Table S7. Association between cytokines and socioeconomic status in the cohorts

		Young Finns Study			FINRISK 1997			FINRISK 2002			Meta-analysis		
cytokine	Socioeconomic status	Beta	(95% CI)	P-value	Beta	(95% CI)	P-value	Beta	(95% CI)	P-value	Beta	(95% CI)	P-value
bNGF	Average	0.1	(-0.11, 0.31)	0.34	NA	NA	NA	-0.049	(-0.17, 0.076)	0.44	-0.0093	(-0.12, 0.098)	0.87
	High	0.092	(-0.13, 0.31)	0.42	NA	NA	NA	-0.071	(-0.19, 0.049)	0.25	-0.035	(-0.14, 0.071)	0.52
CRP	Average	0.058	(-0.15, 0.26)	0.58	-0.067	(-0.14, 0.0065)	0.074	0.017	(-0.10, 0.14)	0.78	-0.036	(-0.096, 0.025)	0.25
	High	-0.28	(-0.50, -0.063)	0.011	-0.2	(-0.27, -0.13)	2.70E-08	-0.14	(-0.26, -0.025)	0.017	-0.19	(-0.25, -0.13)	**1.10E-10**
CTACK	Average	0.22	(0.017, 0.43)	0.034	NA	NA	NA	-0.0056	(-0.13, 0.12)	0.93	0.055	(-0.051, 0.16)	0.31
	High	0.49	(0.27, 0.71)	1.00E-05	NA	NA	NA	0.099	(-0.021, 0.22)	0.1	0.19	(0.086, 0.30)	**0.00037**
Eotaxin	Average	-0.02	(-0.23, 0.19)	0.85	-0.06	(-0.13, 0.014)	0.11	-0.059	(-0.18, 0.068)	0.36	-0.056	(-0.12, 0.0047)	0.07
	High	-0.056	(-0.28, 0.16)	0.62	-0.14	(-0.21, -0.069)	0.00012	0.0058	(-0.12, 0.13)	0.93	-0.1	(-0.16, -0.040)	**0.00098**
FGFBasic	Average	-0.15	(-0.36, 0.052)	0.14	-0.023	(-0.10, 0.056)	0.56	-0.062	(-0.19, 0.063)	0.33	-0.046	(-0.11, 0.018)	0.16
	High	-0.14	(-0.36, 0.076)	0.2	-0.062	(-0.14, 0.015)	0.12	0.0095	(-0.11, 0.13)	0.88	-0.049	(-0.11, 0.013)	0.12
GCSF	Average	0.041	(-0.17, 0.25)	0.69	-0.021	(-0.097, 0.054)	0.58	-0.057	(-0.19, 0.071)	0.38	-0.024	(-0.086, 0.038)	0.45
	High	0.056	(-0.16, 0.28)	0.62	-0.089	(-0.16, -0.016)	0.018	-0.036	(-0.16, 0.088)	0.57	-0.065	(-0.13, -0.0045)	0.035
GROα	Average	0.027	(-0.18, 0.23)	0.8	NA	NA	NA	-0.12	(-0.25, 0.0060)	0.062	-0.081	(-0.19, 0.028)	0.14
	High	0.0023	(-0.22, 0.22)	0.98	NA	NA	NA	-0.097	(-0.22, 0.026)	0.12	-0.073	(-0.18, 0.034)	0.18
HGF	Average	-0.25	(-0.46, -0.044)	0.017	-0.061	(-0.13, 0.013)	0.11	-0.052	(-0.17, 0.069)	0.4	-0.075	(-0.13, -0.015)	0.015
	High	-0.47	(-0.69, -0.25)	2.70E-05	-0.16	(-0.23, -0.089)	9.90E-06	-0.12	(-0.24, -0.0018)	0.047	-0.17	(-0.23, -0.11)	**8.10E-09**
IFN-γ	Average	-0.063	(-0.27, 0.14)	0.55	-0.008	(-0.085, 0.069)	0.84	-0.15	(-0.28, -0.027)	0.018	-0.049	(-0.11, 0.014)	0.13
	High	-0.12	(-0.34, 0.10)	0.29	-0.047	(-0.12, 0.027)	0.21	-0.13	(-0.25, -0.0040)	0.043	-0.073	(-0.13, -0.011)	0.02
IL-10	Average	-0.087	(-0.29, 0.12)	0.41	-0.031	(-0.11, 0.047)	0.44	-0.054	(-0.18, 0.069)	0.39	-0.042	(-0.11, 0.021)	0.19
	High	-0.24	(-0.45, -0.017)	0.035	-0.055	(-0.13, 0.021)	0.15	-0.082	(-0.20, 0.037)	0.18	-0.077	(-0.14, -0.015)	0.015
IL-12p70	Average	-0.059	(-0.26, 0.15)	0.58	-0.047	(-0.12, 0.027)	0.21	-0.014	(-0.14, 0.11)	0.82	-0.04	(-0.10, 0.020)	0.19
	High	-0.26	(-0.48, -0.039)	0.021	-0.13	(-0.20, -0.057)	0.00041	-0.026	(-0.14, 0.092)	0.66	-0.11	(-0.17, -0.054)	**0.00018**
IL-13	Average	-0.076	(-0.28, 0.13)	0.47	NA	NA	NA	-0.037	(-0.16, 0.090)	0.57	-0.047	(-0.16, 0.060)	0.39
	High	-0.22	(-0.44, -0.0053)	0.045	NA	NA	NA	-0.028	(-0.15, 0.095)	0.65	-0.075	(-0.18, 0.032)	0.17
IL-16	Average	-0.066	(-0.28, 0.15)	0.55	NA	NA	NA	-0.016	(-0.14, 0.11)	0.8	-0.028	(-0.14, 0.079)	0.61
	High	-0.072	(-0.30, 0.16)	0.54	NA	NA	NA	-0.1	(-0.22, 0.017)	0.093	-0.096	(-0.20, 0.0098)	0.075
IL-17	Average	-0.051	(-0.26, 0.16)	0.63	-0.067	(-0.14, 0.0091)	0.084	-0.031	(-0.16, 0.097)	0.64	-0.057	(-0.12, 0.0054)	0.073
	High	-0.025	(-0.24, 0.19)	0.82	-0.095	(-0.17, -0.021)	0.012	0.044	(-0.080, 0.17)	0.48	-0.056	(-0.12, 0.0055)	0.075
IL-18	Average	-0.18	(-0.39, 0.022)	0.081	NA	NA	NA	0.019	(-0.11, 0.14)	0.77	-0.035	(-0.14, 0.071)	0.52

cytokine	Socioeconomic status	Young Finns Study			FINRISK 1997			FINRISK 2002			Meta-analysis		
		Beta	(95% CI)	P-value	Beta	(95% CI)	P-value	Beta	(95% CI)	P-value	Beta	(95% CI)	P-value
	High	-0.32	(-0.54, -0.099)	0.0044	NA	NA	NA	-0.046	(-0.16, 0.074)	0.45	-0.11	(-0.21, -0.0034)	0.043
IL-1β	Average	-0.097	(-0.30, 0.11)	0.36	NA	NA	NA	-0.12	(-0.26, 0.017)	0.086	-0.11	(-0.23, 0.0011)	0.052
	High	-0.057	(-0.28, 0.16)	0.61	NA	NA	NA	-0.09	(-0.22, 0.044)	0.19	-0.081	(-0.19, 0.033)	0.16
IL-1ra	Average	-0.0068	(-0.21, 0.20)	0.95	NA	NA	NA	-0.052	(-0.18, 0.072)	0.41	-0.04	(-0.15, 0.066)	0.46
	High	-0.035	(-0.25, 0.18)	0.76	NA	NA	NA	-0.042	(-0.16, 0.078)	0.49	-0.04	(-0.15, 0.065)	0.45
IL-2	Average	0.14	(-0.069, 0.35)	0.19	NA	NA	NA	-0.089	(-0.22, 0.042)	0.18	-0.024	(-0.13, 0.086)	0.67
	High	0.17	(-0.048, 0.39)	0.12	NA	NA	NA	9.70E-06	(-0.13, 0.13)	1	0.043	(-0.067, 0.15)	0.44
IL-2ra	Average	0.069	(-0.14, 0.28)	0.51	NA	NA	NA	-0.069	(-0.19, 0.053)	0.26	-0.034	(-0.14, 0.071)	0.53
	High	-0.019	(-0.24, 0.20)	0.87	NA	NA	NA	-0.082	(-0.20, 0.035)	0.17	-0.068	(-0.17, 0.035)	0.2
IL-4	Average	-0.037	(-0.24, 0.17)	0.73	-0.0061	(-0.080, 0.068)	0.87	-0.081	(-0.21, 0.047)	0.21	-0.026	(-0.087, 0.035)	0.41
	High	0.0036	(-0.22, 0.22)	0.97	-0.083	(-0.15, -0.011)	0.023	-0.082	(-0.21, 0.041)	0.19	-0.076	(-0.14, -0.017)	0.012
IL-5	Average	-0.12	(-0.33, 0.083)	0.24	NA	NA	NA	-0.00023	(-0.13, 0.13)	1	-0.037	(-0.15, 0.075)	0.52
	High	-0.2	(-0.42, 0.015)	0.068	NA	NA	NA	-0.0064	(-0.14, 0.12)	0.92	-0.058	(-0.17, 0.054)	0.31
IL-6	Average	-0.02	(-0.23, 0.19)	0.85	-0.053	(-0.13, 0.021)	0.16	-0.038	(-0.16, 0.088)	0.55	-0.047	(-0.11, 0.014)	0.13
	High	-0.036	(-0.26, 0.18)	0.75	-0.13	(-0.20, -0.058)	0.00039	-0.11	(-0.23, 0.012)	0.077	-0.12	(-0.18, -0.058)	**9.80E-05**
IL-7	Average	-0.017	(-0.22, 0.19)	0.88	NA	NA	NA	-0.035	(-0.17, 0.098)	0.6	-0.03	(-0.14, 0.082)	0.6
	High	-0.11	(-0.33, 0.11)	0.32	NA	NA	NA	-0.027	(-0.16, 0.10)	0.68	-0.049	(-0.16, 0.062)	0.39
IL-8	Average	-0.096	(-0.30, 0.11)	0.36	NA	NA	NA	-0.1	(-0.23, 0.025)	0.12	-0.1	(-0.21, 0.0079)	0.069
	High	-0.1	(-0.32, 0.12)	0.37	NA	NA	NA	-0.14	(-0.26, -0.017)	0.026	-0.13	(-0.24, -0.023)	0.018
IL-9	Average	-0.074	(-0.28, 0.13)	0.48	NA	NA	NA	-0.079	(-0.20, 0.046)	0.21	-0.077	(-0.18, 0.029)	0.15
	High	-0.11	(-0.33, 0.11)	0.34	NA	NA	NA	-0.035	(-0.15, 0.085)	0.57	-0.052	(-0.16, 0.053)	0.33
IP10	Average	0.058	(-0.15, 0.26)	0.58	NA	NA	NA	-0.056	(-0.18, 0.065)	0.36	-0.027	(-0.13, 0.078)	0.62
	High	0.098	(-0.12, 0.32)	0.38	NA	NA	NA	-0.066	(-0.18, 0.051)	0.27	-0.029	(-0.13, 0.074)	0.58
MCSF	Average	-0.064	(-0.38, 0.25)	0.69	NA	NA	NA	-0.04	(-0.16, 0.085)	0.53	-0.044	(-0.16, 0.072)	0.46
	High	-0.18	(-0.51, 0.15)	0.29	NA	NA	NA	-0.015	(-0.14, 0.10)	0.81	-0.034	(-0.15, 0.079)	0.56
MCP-1	Average	-0.029	(-0.24, 0.18)	0.78	-0.048	(-0.12, 0.026)	0.21	-0.15	(-0.27, -0.029)	0.015	-0.071	(-0.13, -0.011)	0.021
	High	0.0049	(-0.21, 0.22)	0.96	-0.086	(-0.16, -0.014)	0.019	-0.13	(-0.25, -0.011)	0.032	-0.09	(-0.15, -0.031)	0.0027
MCP-3	Average	-0.42	(-1.2, 0.40)	0.31	NA	NA	NA	0.13	(-0.043, 0.30)	0.14	0.11	(-0.063, 0.28)	0.22
	High	-0.4	(-1.2, 0.45)	0.36	NA	NA	NA	-0.022	(-0.19, 0.15)	0.8	-0.037	(-0.20, 0.13)	0.67
MIF	Average	0.054	(-0.15, 0.26)	0.61	NA	NA	NA	-0.081	(-0.21, 0.050)	0.23	-0.042	(-0.15, 0.069)	0.46
	High	0.0033	(-0.22, 0.22)	0.98	NA	NA	NA	-0.15	(-0.27, -0.022)	0.021	-0.11	(-0.22, -0.0016)	0.047

cytokine	Socioeconomic status	Young Finns Study			FINRISK 1997			FINRISK 2002			Meta-analysis		
		Beta	(95% CI)	P-value	Beta	(95% CI)	P-value	Beta	(95% CI)	P-value	Beta	(95% CI)	P-value
MIG	Average	0.17	(-0.036, 0.38)	0.1	NA	NA	NA	-0.075	(-0.20, 0.048)	0.23	-0.011	(-0.12, 0.094)	0.84
	High	0.29	(0.072, 0.51)	0.0092	NA	NA	NA	-0.03	(-0.15, 0.088)	0.61	0.042	(-0.062, 0.15)	0.43
MIP-1α	Average	-0.14	(-0.35, 0.068)	0.19	NA	NA	NA	-0.1	(-0.23, 0.023)	0.11	-0.11	(-0.22, -0.0053)	0.04
	High	-0.16	(-0.38, 0.056)	0.14	NA	NA	NA	-0.0063	(-0.13, 0.12)	0.92	-0.044	(-0.15, 0.063)	0.42
MIP-1β	Average	-0.039	(-0.25, 0.17)	0.71	-0.1	(-0.18, -0.030)	0.0059	0.011	(-0.11, 0.13)	0.86	-0.071	(-0.13, -0.010)	0.022
	High	0.012	(-0.21, 0.23)	0.92	-0.17	(-0.24, -0.099)	2.70E-06	-0.084	(-0.20, 0.035)	0.17	-0.14	(-0.20, -0.078)	**5.50E-06**
PDGFbb	Average	-0.096	(-0.30, 0.11)	0.36	-0.018	(-0.092, 0.056)	0.63	-0.07	(-0.19, 0.052)	0.26	-0.037	(-0.098, 0.023)	0.23
	High	-0.19	(-0.41, 0.031)	0.093	-0.074	(-0.15, -0.0028)	0.042	-0.011	(-0.13, 0.11)	0.86	-0.067	(-0.13, -0.0078)	0.027
RANTES	Average	-0.024	(-0.24, 0.19)	0.82				0.014	(-0.11, 0.14)	0.83	0.0042	(-0.10, 0.11)	0.94
	High	0.003	(-0.23, 0.23)	0.98	NA	NA	NA	-0.093	(-0.21, 0.029)	0.13	-0.072	(-0.18, 0.036)	0.19
SCF	Average	0.093	(-0.11, 0.30)	0.38	-0.056	(-0.13, 0.018)	0.14	-0.094	(-0.22, 0.028)	0.13	-0.053	(-0.11, 0.0075)	0.086
	High	0.12	(-0.098, 0.34)	0.28	-0.068	(-0.14, 0.0034)	0.062	-0.092	(-0.21, 0.026)	0.13	-0.06	(-0.12, -0.0016)	0.044
SCGFβ	Average	0.13	(-0.077, 0.34)	0.22	NA	NA	NA	0.085	(-0.038, 0.21)	0.18	0.097	(-0.0090, 0.20)	0.073
	High	0.19	(-0.030, 0.41)	0.091	NA	NA	NA	0.077	(-0.041, 0.20)	0.2	0.1	(-0.0015, 0.21)	0.053
SDF-1α	Average	0.0095	(-0.21, 0.23)	0.93	-0.0024	(-0.077, 0.072)	0.95	0.066	(-0.064, 0.20)	0.32	0.014	(-0.048, 0.076)	0.65
	High	0.087	(-0.14, 0.32)	0.46	-0.079	(-0.15, -0.0070)	0.032	0.078	(-0.048, 0.20)	0.22	-0.032	(-0.092, 0.029)	0.31
TNF-α	Average	-0.029	(-0.24, 0.18)	0.78	NA	NA	NA	-0.042	(-0.17, 0.089)	0.53	-0.039	(-0.15, 0.072)	0.49
	High	-0.027	(-0.25, 0.19)	0.81	NA	NA	NA	0.011	(-0.12, 0.14)	0.86	0.0019	(-0.11, 0.11)	0.97
TNF-β	Average	0.35	(-0.56, 1.2)	0.45	NA	NA	NA	-0.041	(-0.17, 0.091)	0.54	-0.033	(-0.16, 0.098)	0.62
	High	-0.094	(-1.0, 0.86)	0.84	NA	NA	NA	-0.093	(-0.22, 0.033)	0.15	-0.093	(-0.22, 0.032)	0.14
TRAIL	Average	-0.068	(-0.28, 0.14)	0.52	-0.05	(-0.12, 0.025)	0.19	-0.035	(-0.16, 0.087)	0.57	-0.048	(-0.11, 0.013)	0.12
	High	-0.11	(-0.33, 0.11)	0.32	-0.056	(-0.13, 0.016)	0.13	-0.13	(-0.25, -0.015)	0.027	-0.079	(-0.14, -0.020)	0.0085
VEGF	Average	-0.059	(-0.26, 0.15)	0.58	-0.047	(-0.13, 0.037)	0.27	-0.05	(-0.17, 0.072)	0.42	-0.049	(-0.11, 0.016)	0.14
	High	-0.28	(-0.49, -0.058)	0.013	-0.044	(-0.13, 0.039)	0.3	-0.083	(-0.20, 0.035)	0.17	-0.076	(-0.14, -0.011)	0.021

Rank-normal transformed cytokines were used as a dependent variable and socioeconomic status was treated as independent variable. Analyses were run using linear regression without further adjustments. Low socioeconomic status was used as a reference level. Betas reflect change in cytokine levels in normalized SD units. CI, confidence interval

Table S8. List of 97 genetic variants associated with body mass index (BMI)

					MAF		Beta (SD units)		P-value
SNP	Chr.	Position	Effect allele	Other allele	GIANT (HapMap CEU)	(FR97, FR02, YFS)	GIANT[a]	(FR97, FR02, YFS)[b]	GIANT
rs11165643	1	96924097	T	C	0.425	0.405346	0.0218	0.015022	2.07E-12
rs11583200	1	50559820	T	C	0.375	0.479745	-0.0177	-0.030151	1.48E-08
rs12401738	1	78446761	A	G	0.425	0.328224	0.0211	-0.009623	1.15E-10
rs12566985	1	75002193	A	G	0.425	0.478166	-0.0242	-0.030242	3.28E-15
rs17024393	1	110154688	C	T	0.04167	0.054429	0.0658	0.090242	7.03E-14
rs2820292	1	201784287	C	A	0.4917	0.440029	0.0195	0.008674	1.83E-10
rs3101336	1	72751185	C	T	0.3509	0.349625	0.0334	0.031934	2.66E-26
rs543874	1	177889480	G	A	0.2667	0.167601	0.0482	0.042515	2.62E-35
rs657452	1	49589847	G	A	0.4167	0.430482	-0.0227	-0.019705	5.48E-13
rs977747	1	47684677	G	T	0.4667	0.359959	-0.0167	-0.012792	8.65E-08
rs1016287	2	59305625	C	T	0.325	0.260182	-0.0229	-0.022498	2.25E-11
rs10182181	2	25150296	G	A	0.5	0.423154	0.0307	0.0534	8.78E-24
rs11126666	2	26928811	A	G	0.3083	0.37381	0.0207	0.009203	1.33E-09
rs11688816	2	63053048	A	G	0.4583	0.4801	-0.0172	-0.0001	1.89E-08
rs13021737	2	632348	G	A	0.125	0.155382	0.0601	0.024262	1.11E-50
rs1460676	2	164567689	C	T	0.2167	0.202257	0.0197	0.016086	8.98E-07
rs1528435	2	181550962	T	C	0.4167	0.355265	0.0178	-0.005348	1.20E-08
rs17203016	2	208255518	G	A	0.2	0.201616	0.021	0.010395	8.15E-08
rs2121279	2	143043285	T	C	0.1167	0.235406	0.0245	0.009281	2.31E-08
rs2176040	2	227092802	G	A	0.3917	0.367718	-0.0141	-0.010954	6.06E-06
rs492400	2	219349752	T	C	0.325	0.430801	-0.0158	-0.049264	4.17E-07
rs7599312	2	213413231	A	G	0.2917	0.292837	-0.022	-0.021542	1.17E-10
rs13078960	3	85807590	G	T	0.1833	0.164948	0.0297	0.049284	1.74E-14
rs1516725	3	185824004	C	T	0.0917	0.096878	0.0451	0.015665	1.89E-22
rs16851483	3	141275436	T	G	0.0917	0.048451	0.0483	0.015576	3.55E-10
rs2365389	3	61236462	T	C	0.3417	0.449969	-0.02	0.004411	1.63E-10
rs3849570	3	81792112	A	C	0.3667	0.415592	0.0188	0.008152	2.60E-08

					MAF		Beta (SD units)		P-value
SNP	Chr.	Position	Effect allele	Other allele	GIANT (HapMap CEU)	(FR97, FR02, YFS)	GIANT[a]	(FR97, FR02, YFS)[b]	GIANT
rs6804842	3	25106437	G	A	0.425	0.482853	0.0185	0.039378	2.48E-09
rs10938397	4	45182527	G	A	0.4333	0.489782	0.0402	0.050394	3.21E-38
rs11727676	4	145659064	C	T	0.075	0.076062	-0.0358	-0.030917	2.55E-08
rs13107325	4	103188709	T	C	0.1167	0.013569	0.0477	0.12856	1.83E-12
rs17001654	4	77129568	G	C	0.1583	0.123041	0.0306	-0.008749	7.76E-09
rs2112347	5	75015242	G	T	0.375	0.419745	-0.0261	0.002218	6.19E-17
rs7715256	5	153537893	T	G	0.45	0.414653	-0.0163	-0.022774	1.70E-07
rs13191362	6	163033350	G	A	0.2	0.059789	-0.0277	-0.099366	7.34E-09
rs13201877	6	137675541	G	A	0.0833	0.155163	0.0233	0.030217	2.35E-07
rs2033529	6	40348653	G	A	0.2583	0.334428	0.019	0.031542	1.39E-08
rs205262	6	34563164	G	A	0.2667	0.290866	0.0221	-0.008279	1.75E-10
rs2207139	6	50845490	G	A	0.1	0.198351	0.0447	0.081746	4.13E-29
rs9374842	6	120185665	T	C	0.2583	0.271326	0.0187	0.009034	9.67E-08
rs9400239	6	108977663	C	T	0.3	0.373224	0.0188	0.015848	1.61E-08
rs1167827	7	75163169	G	A	0.4583	0.443808	0.0202	0.014743	6.33E-10
rs2245368	7	76608143	T	C	0.2417	0.213606	-0.0317	-0.05754	3.19E-08
rs6465468	7	95169514	T	G	0.325	0.230702	0.0166	0.030775	2.32E-06
rs9641123	7	93197732	C	G	0.3917	0.366419	0.0191	0.032395	5.00E-07
rs16907751	8	81375457	T	C	0.0417	0.117563	-0.035	-0.011054	1.26E-07
rs17405819	8	76806584	C	T	0.3667	0.292075	-0.0224	0.025081	2.07E-11
rs2033732	8	85079709	C	T	0.2417	0.250709	0.0192	0.006517	4.89E-08
rs10733682	9	129460914	G	A	0.425	0.453219	-0.0174	-0.025469	1.83E-08
rs10968576	9	28414339	G	A	0.2917	0.37658	0.0249	0.016505	6.61E-14
rs1928295	9	120378483	C	T	0.425	0.489147	-0.0188	-0.02265	7.91E-10
rs4740619	9	15634326	C	T	0.4667	0.484142	-0.0179	0.004125	4.56E-09
rs6477694	9	111932342	T	C	0.3583	0.394768	-0.0174	-0.006232	2.67E-08
rs11191560	10	104869038	C	T	0.0583	0.079225	0.0308	0.01625	8.45E-09
rs17094222	10	102395440	C	T	0.2083	0.236668	0.0249	0.034423	5.94E-11
rs7899106	10	87410904	G	A	0.05	0.04349	0.0395	0.005299	2.96E-08

					MAF		Beta (SD units)		P-value
SNP	Chr.	Position	Effect allele	Other allele	GIANT (HapMap CEU)	(FR97, FR02, YFS)	GIANT[a]	(FR97, FR02, YFS)[b]	GIANT
rs7903146	10	114758349	T	C	0.25	0.190931	-0.0234	0.000836	1.11E-11
rs11030104	11	27684517	G	A	0.2	0.16879	-0.0414	-0.074295	5.56E-28
rs12286929	11	115022404	G	A	0.4333	0.483892	0.0217	0.015924	1.31E-12
rs2176598	11	43864278	C	T	0.2	0.267387	-0.0198	-0.033025	2.97E-08
rs3817334	11	47650993	T	C	0.45	0.385471	0.0262	0.007837	5.15E-17
rs4256980	11	8673939	G	C	0.275	0.353129	0.0209	0.047879	2.90E-11
rs11057405	12	122781897	A	G	0.0917	0.090284	-0.0307	-0.01178	2.02E-08
rs7138803	12	50247468	A	G	0.4417	0.377306	0.0315	0.016067	8.15E-24
rs12429545	13	54102206	A	G	0.1	0.134391	0.0334	0.030633	1.09E-12
rs1441264	13	79580919	A	G	0.45	0.320952	0.0175	0.034688	6.04E-08
rs9540493	13	66205704	G	A	0.45	0.482497	-0.0172	-0.008449	1.42E-07
rs9581854 / rs12016871	13	28017782	T	C	0.2333	0.233378	0.0298	0.029408	2.29E-10
rs10132280	14	25928179	A	C	0.3333	0.355187	-0.023	-0.027668	1.14E-11
rs11847697	14	30515112	T	C	0.0417	0.014337	0.0492	-0.040625	3.99E-09
rs12885454	14	29736838	A	C	0.3667	0.359924	-0.0207	-0.060814	1.94E-10
rs7141420	14	79899454	T	C	0.3833	0.404718	0.0235	0.053336	1.23E-14
rs16951275	15	68077168	C	T	0.225	0.158881	-0.0311	-0.032985	1.91E-17
rs3736485	15	51748610	G	A	0.425	0.419138	-0.0176	-0.036412	7.41E-09
rs7164727	15	73093991	T	C	0.225	0.324022	0.018	0.012568	6.83E-08
rs12446632	16	19935389	A	G	0.1333	0.124743	-0.0403	-0.067766	1.48E-18
rs1558902	16	53803574	A	T	0.45	0.420234	0.0818	0.094794	7.51E-153
rs2080454	16	49062590	A	C	0.3917	0.472999	-0.0168	0.008648	6.55E-08
rs2650492	16	28333411	A	G	0.3083	0.270775	0.0207	0.014957	1.92E-09
rs3888190	16	28889486	A	C	0.3583	0.416724	0.0309	0.029686	3.14E-23
rs4787491	16	30015337	G	A	0.386	0.435219	0.0159	0.018292	2.24E-06
rs758747	16	3627358	T	C	0.2667	0.203949	0.0225	-0.000268	7.47E-10
rs9925964	16	31129895	G	A	0.3917	0.38722	-0.0192	-0.006065	8.11E-10
rs1000940	17	5283252	G	A	0.225	0.438004	0.0192	0.015914	1.28E-08
rs12940622	17	78615571	A	G	0.4583	0.337994	-0.0182	-0.023733	2.49E-09

					MAF		Beta (SD units)		P-value
SNP	Chr.	Position	Effect allele	Other allele	GIANT (HapMap CEU)	(FR97, FR02, YFS)	GIANT[a]	(FR97, FR02, YFS)[b]	GIANT
rs9914578	17	2005136	G	C	0.1667	0.246762	0.0201	0.032077	8.99E-08
rs1808579	18	21104888	T	C	0.475	0.497786	-0.0167	-0.033504	4.17E-08
rs6567160	18	57829135	C	T	0.2833	0.179138	0.0556	0.085586	3.93E-53
rs7239883	18	40147671	A	G	0.3167	0.42976	-0.0164	-0.008628	1.63E-07
rs7243357	18	56883319	G	T	0.1333	0.273502	-0.0217	-0.037615	3.86E-08
rs17724992	19	18454825	G	A	0.3083	0.219292	-0.0194	-0.013682	3.42E-08
rs2075650	19	45395619	G	A	0.1417	0.174591	-0.0258	-0.008346	1.25E-08
rs2287019	19	46202172	T	C	0.15	0.212682	-0.036	-0.020781	4.59E-18
rs29941	19	34309532	G	A	0.3333	0.391895	0.0182	0.014796	2.41E-08
rs3810291	19	47569003	A	G	0.375	0.366886	0.0283	0.035644	4.81E-15
rs6091540	20	51087862	T	C	0.275	0.226102	-0.0188	-0.020376	8.02E-08
rs2836754	21	40291740	C	T	0.35	0.457796	0.0164	0.029722	4.16E-07

SNP, single nucleotide polymorphisms; Chr, Chromosome; YFS, Young Finns Study, FR97, FINRISK1997 Study; FR02, FINRISK2002 Study; MAF, minor allele frequency; Beta, effect size.
[a]Betas from published meta-GWAS[6] used to weight BMI GRS; [b]Betas from meta-analysis in Finnish cohorts for comparative purposes only.

Table S9. Association of body mass index (BMI) GRS with measured BMI and potential confounders

	YFS			FINRISK1997			FINRISK 2002			Meta-analysis		
	Beta	(95% CI)	P-value	Beta	(95% CI)	P-value	Beta	(95% CI)	P-value	Beta	(95% CI)	P-value
**Body mass index (kg/m^2)**	5.22	(3.99, 6.45)	$1.46x10^{-16}$	4.25	(3.50, 5.00)	$2.99x10^{-28}$	3.69	(2.39, 5.00)	$3.22x10^{-8}$	4.36	(3.78, 4.93)	**$7.34x10^{-50}$**
**Smoking**	0.002	(-0.02, 0.02)	0.81	0.005	(-0.006, 0.02)	0.37	-0.003	(-0.02, 0.02)	0.76	0.003	(-0.006, 0.01)	0.49
**Alcohol Consumption**	0.002	(-0.002, 0.006)	0.27	-0.004	(-0.007, -0.001)	0.005	-0.001	(-0.006, 0.003)	0.54	-0.002	(-0.004, 0.0002)	0.08
**Socioeconomic Status**												
Average	-0.012	(-0.05, 0.02)	0.50	-0.0007	(-0.01, 0.01)	0.91	-0.01	(-0.03, 0.007)	0.19	-0.005	(-0.01, 0.005)	0.35
High	-0.005	(-0.04, 0.03)	0.77	-0.0065	(-0.02, 0.005)	0.27	-0.02	(-0.04, -0.001)	0.03	-0.010	(-0.02, -0.0004)	**0.04**

Measured BMI was used as a dependent variable and BMI related GRS as an independent variable when looking at association between BMI and BMI-GRS in linear model. Beta reflects change in BMI per unit change in GRS.
BMI related genetic risk score (GRS) was used as a dependent variable and association with each confounder (smoking, alcohol consumption and socioeconomic status) as independent variable was analysed separately using linear model. All models are adjusted for age and sex. Low socioeconomic status was used as a reference level in the association between BMI related GRS and socioeconomic status. Non-smoking status (ex, never and occasional smokers) was used as a reference for the association between BMI related GRS and smoking; Alcohol consumption was measured in grams per week and natural log-transformed for the analyses (one was added to zero values). Betas reflect change in GRS. CI, confidence interval; GRS, Genetic risk score

Table S10. First stage F-statistics and Durbin-Wu-Hausman statistics from one-sample Mendelian randomization analysis

	Young Finns Study				FINRISK 1997				FINRISK 2002			
	F-statistic		Durbin-Wu		F-statistic		Durbin-Wu		F-statistic		Durbin-Wu	
Cytokine	Estimate	P-value	Estimate	P-value	Estimate	P-value	Estimate	P-value	Estimate	P-value	Estimate	P-value
bNGF	63.035	3.43E-15	0.012	0.911	NA	NA	NA	NA	29.556	6.26E-08	1.169	0.280
CRP	66.178	7.20E-16	0.255	0.613	105.236	2.00E-24	4.613	0.032	31.326	2.54E-08	0.041	0.840
CTACK	66.178	7.20E-16	1.521	0.218	NA	NA	NA	NA	28.168	1.26E-07	0.403	0.526
Eotaxin	66.271	6.89E-16	2.463	0.117	104.216	3.28E-24	1.387	0.239	30.093	4.79E-08	6.298	0.012
FGFBasic	65.965	7.99E-16	0.004	0.947	95.566	2.55E-22	0.075	0.785	32.345	1.53E-08	5.991	0.014
GCSF	66.019	7.78E-16	0.536	0.464	95.471	2.53E-22	0.192	0.662	29.555	6.31E-08	0.813	0.367
GROα	61.404	7.56E-15	0.037	0.847	NA	NA	NA	NA	24.702	7.44E-07	1.061	0.303
HGF	66.178	7.20E-16	0.019	0.891	103.049	5.83E-24	4.274	0.039	30.582	3.70E-08	0.126	0.723
IFN-γ	66.178	7.20E-16	0.004	0.947	90.885	2.51E-21	0.005	0.941	27.377	1.90E-07	1.055	0.305
IL-10	66.384	6.51E-16	0	0.988	96.394	1.68E-22	0.059	0.808	28.543	1.04E-07	4.443	0.035
IL-12p70	66.178	7.20E-16	0.079	0.779	102.304	8.44E-24	0.608	0.435	31.562	2.25E-08	1.181	0.277
IL-13	66.178	7.20E-16	0.009	0.923	NA	NA	NA	NA	27.232	2.04E-07	2.675	0.102
IL-16	61.016	9.50E-15	0.451	0.502	NA	NA	NA	NA	29.252	7.28E-08	0.526	0.468
IL-17	66.178	7.20E-16	0.306	0.580	97.985	7.44E-23	0.032	0.859	28.439	1.11E-07	0.338	0.561
IL-18	66.178	7.20E-16	0.244	0.621	NA	NA	NA	NA	28.239	1.22E-07	1.717	0.190
IL-1β	66.019	7.78E-16	0.408	0.523	NA	NA	NA	NA	28.208	1.28E-07	1.095	0.296
IL-1ra	66.178	7.20E-16	0.337	0.561	NA	NA	NA	NA	34.551	5.01E-09	1.743	0.187
IL-2	66.447	6.31E-16	0.004	0.947	NA	NA	NA	NA	33.979	6.81E-09	1.658	0.198
IL-2ra	65.012	1.28E-15	0.184	0.668	NA	NA	NA	NA	30.582	3.70E-08	0.008	0.928
IL-4	66.178	7.20E-16	0.506	0.477	102.931	6.19E-24	1.1	0.294	28.135	1.30E-07	0.448	0.503
IL-5	65.965	7.99E-16	0	0.997	NA	NA	NA	NA	20.362	6.95E-06	5.381	0.020
IL-6	66.019	7.78E-16	0.864	0.353	NA	NA	NA	NA	27.931	1.43E-07	0.801	0.371
IL-7	66.178	7.20E-16	0.627	0.429	103.056	5.81E-24	1.563	0.211	27.567	1.75E-07	1.533	0.216
IL-8	66.178	7.20E-16	1.421	0.233	NA	NA	NA	NA	27.377	1.90E-07	5.072	0.024
IL-9	66.925	4.99E-16	0.079	0.778	NA	NA	NA	NA	34.563	4.98E-09	5.764	0.016
IP10	66.178	7.20E-16	0.028	0.866	NA	NA	NA	NA	30.829	3.26E-08	2.484	0.115
MCSF	22.723	2.21E-06	0.082	0.775	NA	NA	NA	NA	28.198	1.25E-07	1.093	0.296

	Young Finns Study				FINRISK 1997				FINRISK 2002			
	F-statistic		Durbin-Wu		F-statistic		Durbin-Wu		F-statistic		Durbin-Wu	
Cytokine	Estimate	P-value	Estimate	P-value	Estimate	P-value	Estimate	P-value	Estimate	P-value	Estimate	P-value
MCP-1	66.178	7.20E-16	0.051	0.821	103.049	5.83E-24	10.435	0.001	30.829	3.26E-08	0.99	0.320
MCP-3	4.245	0.040	2.013	0.157	NA	NA	NA	NA	14.41	0.00016	5.101	0.024
MIF	65.58	9.66E-16	0.974	0.324	NA	NA	NA	NA	26.851	2.49E-07	0.2	0.655
MIG	66.178	7.20E-16	0.008	0.928	NA	NA	NA	NA	30.829	3.26E-08	0.058	0.810
MIP-1α	66.178	7.20E-16	0.007	0.933	NA	NA	NA	NA	27.401	1.88E-07	1.44	0.230
MIP-1β	66.178	7.20E-16	0.005	0.942	103.049	5.83E-24	0.007	0.935	30.49	3.89E-08	0.017	0.896
PDGFbb	66.178	7.20E-16	0.147	0.701	103.049	5.83E-24	1.053	0.305	30.829	3.26E-08	0.368	0.544
RANTES	59.525	1.97E-14	0.742	0.389	NA	NA	NA	NA	28.255	1.22E-07	3.047	0.081
SCF	65.686	9.17E-16	0.875	0.350	103.049	5.83E-24	1.439	0.230	30.641	3.59E-08	0.17	0.680
SCGFβ	65.651	9.33E-16	1.082	0.298	NA	NA	NA	NA	30.888	3.17E-08	0.074	0.786
SDF-1α	67.546	3.91E-16	0.064	0.800	106.645	1.00E-24	0.824	0.364	17.092	3.76E-05	1.896	0.169
TNF-α	66.178	7.20E-16	0.001	0.972	NA	NA	NA	NA	24.457	8.47E-07	3.995	0.046
TNF-β	2.054	0.155	1.124	0.292	NA	NA	NA	NA	22.932	1.85E-06	0.012	0.914
TRAIL	65.985	7.93E-16	0.112	0.738	100.442	2.14E-23	4.591	0.032	31.111	2.83E-08	0.034	0.854
VEGF	66.178	7.20E-16	0.487	0.485	88.787	7.83E-21	1.261	0.261	30.614	3.64E-08	1.139	0.286

Table S11. Results from one-sample Mendelian randomization analysis using natural log-transformed cytokine concentrations for a subset of cytokines that showed evidence of association with body mass index (BMI).

	YFS			FINRISK1997			FINRISK 2002			Meta-analysis		
Cytokine	Beta	(95% CI)	P-value	Beta	(95% CI)	P-value	Beta	(95% CI)	P-value	Beta	(95% CI)	P-value
CRP	0.33	(0.20, 0.46)	9.90E-07	0.34	(0.23, 0.46)	1.20E-08	0.26	(0.034, 0.48)	0.024	0.33	(0.24, 0.41)	4.60E-15
HGF	0.12	(0.031, 0.21)	0.008	0.15	(0.082, 0.21)	8.50E-06	0.027	(-0.11, 0.16)	0.69	0.12	(0.074, 0.17)	7.20E-07
IL-12p70	0.058	(-0.080, 0.20)	0.41	0.17	(0.035, 0.31)	0.014	0.25	(-0.054, 0.56)	0.11	0.13	(0.035, 0.22)	0.007
IL-6	0.054	(-0.042, 0.15)	0.27	0.13	(0.039, 0.21)	0.0044	0.34	(0.022, 0.66)	0.036	0.1	(0.040, 0.17)	0.0013
IL-7	0.022	(-0.072, 0.12)	0.65				0.26	(-0.13, 0.65)	0.2	0.035	(-0.057, 0.13)	0.46
IP10	0.075	(-0.046, 0.20)	0.22				0.22	(0.021, 0.43)	0.031	0.11	(0.010, 0.22)	0.031
MCP-1	0.017	(-0.066, 0.10)	0.68	0.12	(0.055, 0.18)	0.00025	0.097	(-0.036, 0.23)	0.15	0.083	(0.036, 0.13)	0.00053
MCP-3	0.56	(-0.70, 1.8)	0.38				0.71	(0.082, 1.3)	0.027	0.68	(0.12, 1.2)	0.018
MIP-1β	0.04	(-0.038, 0.12)	0.31	0.089	(0.018, 0.16)	0.014	0.033	(-0.091, 0.16)	0.6	0.062	(0.013, 0.11)	0.012
TRAIL	0.12	(-0.00022, 0.23)	0.051	0.19	(0.064, 0.32)	0.0034	0.0023	(-0.16, 0.17)	0.98	0.12	(0.042, 0.19)	0.0024
VEGF	0.081	(-0.047, 0.21)	0.22	0.26	(0.051, 0.47)	0.015	0.15	(-0.050, 0.35)	0.14	0.13	(0.038, 0.23)	0.006

Estimates correspond to change in natural log-transformed cytokine concentration (pg/ml) and CRP concentration (mg/l) per 1-SD change in BMI. Analyses were done using linear regression and are adjusted for age and sex. One was added to each cytokine value prior to transformation to account for zero values.

Table S12. Summary of SNPs used as instruments in the analysis of BMI-associated inflammation related variables and disease outcomes.

SNP	Chr	Gene: consequence	F-statistic	F-statistic (n,r2,k)	Variance explained	Effect allele	Other allele	Effect allele frequency	Effect (without BMI)	Std. Error (without BMI)	P-value (without BMI)	Effect (BMI adj.)	Std. Error (BMI adj.)	P-value (BMI adj.)
**CRP**									Effects reported relate to natural log-transformed CRP					
**rs1130864**	1	***CRP: Intron Variant***	1000.86	Not available	Not available	A	G	Not available	0.1392	0.0044	Not available	n/a	n/a	n/a
**rs1205**	1	***CRP: 3 Prime UTR Variant***	1829.05			T	C		-0.1839	0.0043				
**rs3093077**	1	-	659.58			A	C		-0.2183	0.0085				
**HGF**									Effects reported relate to SD units					
rs3748034	4	*HGFAC: Missense Variant*	43.06	43.66	0.0052	T	G	0.1278	0.1529	0.0233	5.21E-11	0.1495	0.0234	1.81E-10
**rs5745687**	7	***HGF: Missense Variant***	55.44	54.79	0.0065	T	C	0.0375	-0.3008	0.0404	9.92E-14	-0.3072	0.0406	2.75E-14
rs16844383	4	*HGFAC: Intron Variant*	42.40	43.46	0.0052	T	C	0.1468	0.1439	0.0221	7.68E-11	0.1417	0.0222	1.98E-10
**MCP-1**									Effects reported relate to SD units					
rs12075	1	*ACKR1: Missense Variant*	201.49	215.79	0.0238	A	G	0.523	0.2186	0.0154	1.36E-45	0.2185	0.0155	1.44E-44
rs138591554	3	*CCR3: Intron Variant*	94.68	112.13	0.0125	A	T	0.935	-0.3211	0.033	1.99E-22	-0.3171	0.0331	7.94E-22
rs2036297	3	*LOC105377067: Non-Coding Transcript Variant*	54.58	58.78	0.0066	A	G	0.384	0.1182	0.016	1.30E-13	0.119	0.016	1.09E-13
rs2228467	3	*ACKR2: Missense Variant*	81.62	88.20	0.0099	T	C	0.9219	-0.262	0.029	1.60E-19	-0.2637	0.0291	9.19E-20
rs7632755	3	-	89.74	96.25	0.0108	A	G	0.0647	0.2984	0.0315	2.79E-21	0.2938	0.0316	1.18E-20
rs2427837	1	*FCER1A: Intron Variant*	42.98	46.54	0.0052	A	G	0.2963	0.1121	0.0171	5.35E-11	0.1106	0.0171	1.13E-10
**TRAIL**									Effects reported relate to SD units					
rs11081739	18	-	47.69	52.49	0.0063	A	G	0.2048	0.1395	0.0202	4.62E-12	0.1411	0.0202	3.34E-12
rs183815186	18	-	33.78	39.09	0.0047	A	T	0.9803	-0.3499	0.0602	6.34E-09	-0.3564	0.0604	3.52E-09
rs193112415	18	*LOC105372049: Intron Variant*	284.41	351.46	0.0410	T	C	0.9809	-1.0456	0.062	1.01E-63	-1.0421	0.0623	2.15E-62
rs57396456	18	*LOC105372047: Intron Variant*	119.51	127.08	0.0152	T	C	0.9755	-0.5641	0.0516	7.71E-28	-0.5626	0.0518	1.25E-27
rs62093514	18	*B4GALT6: Intron Variant*	362.94	442.54	0.0510	T	C	0.0239	1.0459	0.0549	5.80E-81	1.0618	0.0552	6.86E-82
rs62093947	18	*RNF125: Intron Variant*	272.50	326.26	0.0381	T	C	0.9656	-0.7577	0.0459	2.74E-61	-0.7596	0.046	3.31E-61
rs664216	18	*TRAPPC8: Intron Variant*	34.15	35.90	0.0043	A	T	0.8617	-0.135	0.0231	4.79E-09	-0.1385	0.0231	2.02E-09
rs679163	18	-	46.85	48.17	0.0058	C	G	0.6018	0.1102	0.0161	7.99E-12	0.1066	0.0162	4.24E-11
rs74778900	18	-	118.94	130.25	0.0156	T	C	0.0238	0.5791	0.0531	9.90E-28	0.5906	0.0532	2.59E-28

SNP	Chr	Gene: consequence	F-statistic	F-statistic (n,r2,k)	Variance explained	Effect allele	Other allele	Effect allele frequency	Effect (without BMI)	Std. Error (without BMI)	P-value (without BMI)	Effect (BMI adj.)	Std. Error (BMI adj.)	P-value (BMI adj.)
rs77451439	18	-	138.51	142.93	0.0171	A	G	0.9522	-0.4331	0.0368	6.10E-32	-0.4322	0.0369	1.21E-31
**rs79287178**	3	***LINC02068: Intron Variant***	105.01	115.57	0.0139	A	G	0.0389	-0.4304	0.042	1.17E-24	-0.4317	0.0421	9.12E-25
rs9952273	18	-	305.51	356.28	0.0415	T	C	0.0283	0.8687	0.0497	1.62E-68	0.864	0.0499	3.86E-69
**rs17600346**	3	***TNFSF10: Non-Coding Transcript Variant***	98.03	97.17	0.0117	T	C	0.9552	0.3693	0.0373	3.87E-23	0.3745	0.0374	1.20E-23
rs2912332	18	-	68.78	67.92	0.0082	A	G	0.9016	-0.2148	0.0259	1.19E-16	-0.2139	0.026	1.47E-16
rs3859363	18	*DSG1-AS1: Intron Variant / DSG4: Intron Variant*	133.54	136.27	0.0163	T	C	0.9519	-0.4218	0.0365	8.27E-31	-0.4172	0.0367	6.92E-30
rs62095573	18	*RNF138: Intron Variant*	169.21	209.78	0.0249	T	G	0.9431	-0.4813	0.037	1.37E-38	-0.4773	0.0371	9.61E-38
rs6506966	18	*GAREM1: Synonymous Variant*	64.99	71.10	0.0086	A	G	0.0838	0.2362	0.0293	7.97E-16	0.2348	0.0294	1.75E-15
rs75582879	18	*LOC105372047: Intron Variant*	117.38	123.32	0.0148	T	G	0.9754	-0.5547	0.0512	2.42E-27	-0.5531	0.0514	3.96E-27
rs76100852	18	-	138.96	141.95	0.0170	T	C	0.0473	0.4338	0.0368	5.07E-32	0.4336	0.0369	7.72E-32
rs10502579	18	-	305.44	350.68	0.0409	T	C	0.9719	-0.8651	0.0495	1.95E-68	-0.8602	0.0497	6.28E-69
rs12607805	18	*DSG2: Intron Variant*	43.10	50.89	0.0061	A	G	0.8723	-0.1661	0.0253	4.93E-11	-0.174	0.0254	8.20E-12
rs2162379	18	*RNF125: Intron Variant*	33.71	35.42	0.0043	A	G	0.7059	-0.1016	0.0175	6.72E-09	-0.1043	0.0176	2.95E-09
rs138987090	18	-	94.06	124.24	0.0149	A	G	0.9857	-0.7264	0.0749	2.97E-22	-0.7497	0.0752	4.50E-23
**rs3136588**	3	***TNFSF10: Intron Variant***	99.89	99.69	0.0120	T	G	0.0451	-0.3728	0.0373	1.74E-23	-0.3782	0.0374	5.00E-24
**rs3136594**	3	***TNFSF10: Intron Variant***	62.42	62.96	0.0076	A	G	0.2951	-0.1351	0.0171	2.60E-15	-0.1361	0.0171	1.88E-15
rs72918954	18	-	33.84	36.41	0.0044	A	G	0.1008	0.1559	0.0268	6.16E-09	0.159	0.0269	3.95E-09
rs72965213	18	-	37.85	45.69	0.0055	T	C	0.0234	0.3476	0.0565	7.84E-10	0.3636	0.0566	9.37E-11
rs9953120	18	*MEP1B: Intron Variant*	40.51	40.31	0.0049	T	C	0.6095	0.1012	0.0159	1.99E-10	0.099	0.016	5.26E-10
rs112821861	18	-	300.68	346.01	0.0404	T	G	0.9717	-0.8566	0.0494	1.89E-67	-0.8518	0.0496	4.90E-68
rs113370271	18	*TRAPPC8: Intron Variant*	138.97	143.05	0.0171	A	G	0.0474	0.435	0.0369	4.53E-32	0.4347	0.037	7.99E-32
rs117618570	18	*DSG1-AS1: 2KB Upstream Variant*	160.46	184.18	0.0219	T	G	0.9599	-0.5333	0.0421	1.01E-36	-0.5294	0.0423	5.62E-36
rs148508102	18	*DSG1: Intron Variant*	281.00	355.75	0.0414	T	C	0.019	1.0544	0.0629	4.77E-63	1.0498	0.0631	1.63E-61
rs6506939	18	-	32.11	35.70	0.0043	T	C	0.392	0.0952	0.0168	1.48E-08	0.0927	0.0169	4.17E-08

Formula used to calculate F-statistic: $(\text{beta}_{\text{exposure}})^2 / (\text{SE beta}_{\text{exposure}})^2$
Formula used to calculate F-statistic (n,r2,k): $((n - k - 1) / k) \times (r^2 / (1 - r^2))$, where n is sample size, k is number of instruments, and r^2 refers to variance explained by the instrument.
Formula used to calculate variance explained: $(\text{beta}_{\text{exposure}})^2 \times (2 \times \text{frequency} (1 - \text{frequency}))$, where frequency refers to allele frequency.
Cis variants for each inflammation related variable are bolded in the table.

Table S13. Complete list of CRP instruments – see excel file

Table S14. Complete list of HGF instruments – see excel file

Table S15. Complete list of MCP-1 instruments – see excel file

Table S16. Complete list of TRAIL instruments – see excel file

Table S17. Association between rs12075 and circulating MCP-1: cohort-specific results and meta-analysis results with and without FINRISK 2002

	Sample material	SNP	Effect allele	Non-effect allele	Beta	SE	p-value	N	Effect allele frequency	Heterogeneity Chi Squared	Heterogeneity df	Heterogeneity p-value
**Cohort specific**												
YFS	Serum	rs12075	A	G	0.7242	0.031	4.49E-121	2019	0.53	-	-	-
FR97	EDTA plasma	rs12075	A	G	0.0722	0.021	0.00045841	4613	0.51	-	-	-
FR02	Heparin plasma	rs12075	A	G	0.00066	0.034	0.984718	1705	0.54	-	-	-
**Meta-analyses**												
YFS & FR97	-	rs12075	A	G	0.2735	0.017	1.44E-56	6632	0.52	304.596	1	3.28E-68
YFS & FR97 & FR02	-	rs12075	A	G	0.2186	0.015	1.36E-45	8337	0.52	354.926	2	8.49E-78

df, degrees of freedom

Table S18. Results from the two-sample MR analysis between BMI-driven inflammation related variables and disease outcomes

Outcome	Method	N SNP	Beta	SE	p-value	95% CI lower	95% CI upper	OR	OR 95% CI lower	OR 95% CI upper
**CRP**										
Schizophrenia	Inverse variance weighted	3	-0.0941	0.0434	0.0302	-0.1792	-0.0090	0.9102	0.8359	0.9910
Alzheimer's disease	Inverse variance weighted	3	0.0384	0.0651	0.5549	-0.0891	0.1659	1.0392	0.9147	1.1805
Rheumatoid arthritis	Inverse variance weighted	3	-0.0760	0.0576	0.1873	-0.1889	0.0370	0.9268	0.8278	1.0376
Inflammatory bowel disease (immunochip)	Inverse variance weighted	3	-0.0322	0.0521	0.5370	-0.1343	0.0699	0.9683	0.8743	1.0725
Crohn's disease (immunochip)	Inverse variance weighted	3	0.0055	0.0760	0.9419	-0.1434	0.1544	1.0056	0.8664	1.1670
Ulcerative colitis (immunochip)	Inverse variance weighted	3	-0.0688	0.0541	0.2031	-0.1749	0.0372	0.9335	0.8396	1.0379
Inflammatory bowel disease (GWAS)	Inverse variance weighted	3	0.0128	0.0973	0.8954	-0.1778	0.2034	1.0129	0.8371	1.2256
Crohn's disease (GWAS)	Inverse variance weighted	3	0.2472	0.1533	0.1068	-0.0532	0.5477	1.2804	0.9481	1.7292
Ulcerative colitis (GWAS)	Inverse variance weighted	3	-0.0916	0.0877	0.2963	-0.2636	0.0803	0.9124	0.7683	1.0836
Coronary artery disease	Inverse variance weighted	3	0.0905	0.0597	0.1292	-0.0264	0.2074	1.0947	0.9739	1.2305
Coronary artery disease (plus C4D)	Inverse variance weighted	3	-0.0052	0.0373	0.8887	-0.0784	0.0679	0.9948	0.9246	1.0703
Myocardial infarction	Inverse variance weighted	3	0.0602	0.0414	0.1456	-0.0209	0.1413	1.0621	0.9793	1.1518
Type 2 diabetes	Inverse variance weighted	3	0.0522	0.0589	0.3756	-0.0632	0.1675	1.0535	0.9387	1.1824
Ovarian cancer	Inverse variance weighted	3	-0.0791	0.0843	0.3483	-0.2443	0.0862	0.9240	0.7832	1.0900
Breast cancer (overall)	Inverse variance weighted	3	-0.0005	0.0261	0.9862	-0.0516	0.0507	0.9996	0.9497	1.0520
ER+ Breast cancer	Inverse variance weighted	3	0.0070	0.0312	0.8218	-0.0541	0.0682	1.0070	0.9473	1.0705
ER- Breast cancer	Inverse variance weighted	3	0.0437	0.0475	0.3572	-0.0493	0.1368	1.0447	0.9519	1.1466
**HGF**										
Schizophrenia	Inverse variance weighted	2	-0.0988	0.0589	0.0936	-0.2142	0.0167	0.9059	0.8072	1.0168
Alzheimer's disease	Inverse variance weighted	2	0.0007	0.0888	0.9933	-0.1734	0.1748	1.0007	0.8408	1.1910
Rheumatoid arthritis	Inverse variance weighted	2	0.1811	0.0988	0.0669	-0.0126	0.3748	1.1985	0.9875	1.4547
Inflammatory bowel disease (immunochip)	Wald ratio	1	-0.0216	0.0690	0.7543	-0.1568	0.1137	0.9786	0.8548	1.1204
Crohn's disease (immunochip)	Wald ratio	1	0.0672	0.0836	0.4217	-0.0967	0.2311	1.0695	0.9078	1.2599
Ulcerative colitis (immunochip)	Wald ratio	1	-0.1680	0.0861	0.0510	-0.3367	0.0007	0.8454	0.7141	1.0007
Inflammatory bowel disease (GWAS)	Inverse variance weighted	2	0.0160	0.0938	0.8646	-0.1678	0.1998	1.0161	0.8455	1.2212

Outcome	Method	N SNP	Beta	SE	p-value	95% CI lower	95% CI upper	OR	OR 95% CI lower	OR 95% CI upper
Crohn's disease (GWAS)	Inverse variance weighted	2	0.1184	0.1271	0.3518	-0.1308	0.3676	1.1257	0.8774	1.4442
Ulcerative colitis (GWAS)	Inverse variance weighted	2	-0.0893	0.1183	0.4505	-0.3211	0.1426	0.9146	0.7253	1.1533
Coronary artery disease	Inverse variance weighted	2	-0.1351	0.0896	0.1318	-0.3108	0.0406	0.8736	0.7328	1.0414
Coronary artery disease (plus C4D)	Inverse variance weighted	2	0.0353	0.0564	0.5321	-0.0753	0.1459	1.0359	0.9274	1.1570
Myocardial infarction	Inverse variance weighted	2	0.0414	0.0631	0.5114	-0.0822	0.1651	1.0423	0.9211	1.1795
Type 2 diabetes	Inverse variance weighted	2	-0.1600	0.1375	0.2447	-0.4294	0.1095	0.8522	0.6509	1.1158
Ovarian cancer	Inverse variance weighted	2	0.0532	0.0754	0.4805	-0.0946	0.2011	1.0547	0.9097	1.2228
Breast cancer (overall)	Inverse variance weighted	2	-0.0326	0.0400	0.4147	-0.1111	0.0458	0.9679	0.8949	1.0469
ER+ Breast cancer	Inverse variance weighted	2	-0.0229	0.0424	0.5896	-0.1060	0.0603	0.9774	0.8994	1.0621
ER- Breast cancer	Inverse variance weighted	2	-0.0552	0.0814	0.4977	-0.2148	0.1044	0.9463	0.8067	1.1100
**MCP-1**										
Schizophrenia	Inverse variance weighted	4	-0.0663	0.0446	0.1372	-0.1538	0.0211	0.9358	0.8574	1.0214
Alzheimer's disease	Inverse variance weighted	4	-0.0489	0.0585	0.4033	-0.1634	0.0657	0.9523	0.8492	1.0679
Rheumatoid arthritis	Inverse variance weighted	4	-0.0456	0.0497	0.3586	-0.1431	0.0518	0.9554	0.8667	1.0532
Inflammatory bowel disease (immunochip)	Inverse variance weighted	3	-0.1399	0.0468	0.0028	-0.2317	-0.0481	0.8695	0.7932	0.9531
Crohn's disease (immunochip)	Inverse variance weighted	3	-0.0948	0.0564	0.0926	-0.2053	0.0157	0.9096	0.8144	1.0158
Ulcerative colitis (immunochip)	Inverse variance weighted	3	-0.1767	0.0669	0.0083	-0.3078	-0.0455	0.8381	0.7351	0.9555
Inflammatory bowel disease (GWAS)	Inverse variance weighted	4	0.0807	0.0802	0.3146	-0.0766	0.2379	1.0840	0.9263	1.2686
Crohn's disease (GWAS)	Inverse variance weighted	4	0.1848	0.0717	0.0099	0.0444	0.3253	1.2030	1.0454	1.3845
Ulcerative colitis (GWAS)	Inverse variance weighted	4	0.0316	0.1012	0.7545	-0.1667	0.2299	1.0321	0.8465	1.2585
Coronary artery disease	Inverse variance weighted	4	0.0147	0.0530	0.7810	-0.0891	0.1186	1.0148	0.9148	1.1259
Coronary artery disease (plus C4D)	Inverse variance weighted	4	0.0190	0.0287	0.5072	-0.0372	0.0753	1.0192	0.9635	1.0782
Myocardial infarction	Inverse variance weighted	4	0.0135	0.0320	0.6736	-0.0493	0.0763	1.0136	0.9519	1.0793
Type 2 diabetes	Inverse variance weighted	4	0.0094	0.0493	0.8485	-0.0872	0.1060	1.0095	0.9165	1.1118
Ovarian cancer	Inverse variance weighted	4	-0.0535	0.0399	0.1797	-0.1317	0.0247	0.9479	0.8766	1.0250
Breast cancer (overall)	Inverse variance weighted	4	-0.0182	0.0189	0.3349	-0.0553	0.0188	0.9819	0.9462	1.0190
ER+ Breast cancer	Inverse variance weighted	4	-0.0036	0.0224	0.8738	-0.0476	0.0404	0.9964	0.9536	1.0412

Outcome	Method	N SNP	Beta	SE	p-value	95% CI lower	95% CI upper	OR	OR 95% CI lower	OR 95% CI upper
ER- Breast cancer	Inverse variance weighted	4	-0.0486	0.0343	0.1566	-0.1158	0.0186	0.9526	0.8907	1.0188
**TRAIL**										
Schizophrenia	Inverse variance weighted	11	0.0062	0.0184	0.7371	-0.0299	0.0423	1.0062	0.9705	1.0432
Alzheimer's disease	Inverse variance weighted	10	0.0146	0.0365	0.6900	-0.0570	0.0861	1.0147	0.9446	1.0899
Rheumatoid arthritis	Inverse variance weighted	13	-0.0198	0.0274	0.4705	-0.0734	0.0339	0.9804	0.9292	1.0345
Inflammatory bowel disease (immunochip)	Wald ratio	1	0.0286	0.0472	0.5441	-0.0639	0.1211	1.0290	0.9381	1.1287
Crohn's disease (immunochip)	Wald ratio	1	-0.0156	0.0575	0.7855	-0.1283	0.0970	0.9845	0.8796	1.1019
Ulcerative colitis (immunochip)	Wald ratio	1	0.0634	0.0580	0.2742	-0.0503	0.1771	1.0655	0.9510	1.1938
Inflammatory bowel disease (GWAS)	Inverse variance weighted	13	0.0321	0.0331	0.3310	-0.0327	0.0970	1.0327	0.9679	1.1018
Crohn's disease (GWAS)	Inverse variance weighted	13	0.0284	0.0373	0.4453	-0.0446	0.1015	1.0289	0.9564	1.1068
Ulcerative colitis (GWAS)	Inverse variance weighted	13	0.0424	0.0415	0.3066	-0.0389	0.1237	1.0433	0.9619	1.1316
Coronary artery disease	Inverse variance weighted	7	0.0034	0.0319	0.9143	-0.0591	0.0660	1.0034	0.9426	1.0682
Coronary artery disease (plus C4D)	Inverse variance weighted	12	0.0296	0.0144	0.0404	0.0013	0.0578	1.0300	1.0013	1.0595
Myocardial infarction	Inverse variance weighted	12	0.0291	0.0161	0.0702	-0.0024	0.0606	1.0295	0.9976	1.0624
Type 2 diabetes	Inverse variance weighted	9	-0.0162	0.0155	0.2975	-0.0466	0.0142	0.9840	0.9545	1.0143
Ovarian cancer	Inverse variance weighted	13	0.0165	0.0195	0.3979	-0.0217	0.0547	1.0166	0.9785	1.0563
Breast cancer (overall)	Inverse variance weighted	13	-0.0212	0.0113	0.0607	-0.0434	0.0010	0.9790	0.9575	1.0010
ER+ Breast cancer	Inverse variance weighted	13	-0.0176	0.0141	0.2129	-0.0452	0.0101	0.9826	0.9558	1.0101
ER- Breast cancer	Inverse variance weighted	13	-0.0283	0.0180	0.1167	-0.0635	0.0070	0.9721	0.9384	1.0071

SNP, single nucleotide polymorphism; OR odds ratio; CI confidence interval

Table S19. A) Gender specific SNP-inflammatory variable associations in each cohort for SNPs used as instruments for ovarian and breast cancer

SNP	CHR	Effect allele	Other allele	Beta Females	se females	p-value females	Beta males	se males	p-value males	Beta original	se original	p-value original
**YFS**												
**CRP**												
**rs1130864**	**1**	A	G	0.195	0.045	1.53E-05	0.246	0.049	5.85E-07	0.139	0.004	NA
**rs1205**	**1**	T	C	-0.166	0.045	2.41E-04	-0.269	0.049	3.63E-08	-0.184	0.004	NA
**rs3093077**	**1**	C	A	0.250	0.088	4.65E-03	0.203	0.098	3.88E-02	0.218	0.009	NA
**HGF**												
rs3748034	4	T	G	0.121	0.067	6.81E-02	0.085	0.073	2.49E-01	0.153	0.02	5.21E-11
**rs5745687**	**7**	T	C	-0.055	0.109	6.16E-01	-0.462	0.118	9.16E-05	-0.301	0.04	9.92E-14
**MCP-1**												
rs12075	1	A	G	0.706	0.043	2.31E-60	0.747	0.045	6.78E-61	0.219	0.02	1.36E-45
rs138591554	3	T	A	0.296	0.086	5.92E-04	0.153	0.101	1.31E-01	0.321	0.03	1.99E-22
rs2036297	3	G	A	-0.036	0.045	4.15E-01	-0.083	0.048	8.12E-02	-0.118	0.02	1.30E-13
rs2228467	3	C	T	0.204	0.086	1.75E-02	0.317	0.086	2.44E-04	0.262	0.03	1.60E-19
**TRAIL**												
rs11081739	18	G	A	-0.080	0.057	1.56E-01	-0.148	0.062	1.62E-02	-0.140	0.02	4.62E-12
rs138987090	18	G	A	1.104	0.220	5.40E-07	0.512	0.249	3.98E-02	0.726	0.07	2.97E-22
rs183815186	18	T	A	0.660	0.165	6.09E-05	0.352	0.188	6.14E-02	0.350	0.06	6.34E-09
rs193112415	18	C	T	1.094	0.176	4.99E-10	1.095	0.170	1.30E-10	1.046	0.06	1.01E-63
rs57396456	18	C	T	0.312	0.141	2.70E-02	1.047	0.157	2.82E-11	0.564	0.05	7.71E-28
rs62093514	18	T	C	1.012	0.156	8.94E-11	1.251	0.157	1.54E-15	1.046	0.05	5.80E-81
rs62093947	18	C	T	0.596	0.123	1.22E-06	1.046	0.143	2.69E-13	0.758	0.05	2.74E-61
rs6506939	18	C	T	-0.021	0.047	6.50E-01	-0.123	0.053	2.00E-02	-0.095	0.02	1.48E-08
rs679163	18	G	C	-0.082	0.045	6.67E-02	-0.129	0.049	8.03E-03	-0.110	0.02	7.99E-12
rs74778900	18	T	C	0.332	0.121	5.86E-03	1.044	0.160	7.44E-11	0.579	0.05	9.90E-28
rs77451439	18	G	A	0.418	0.107	8.83E-05	0.474	0.111	1.96E-05	0.433	0.04	6.10E-32
**rs79287178**	**3**	A	G	-0.442	0.121	2.76E-04	-0.369	0.118	1.82E-03	-0.430	0.04	1.17E-24
rs9952273	18	T	C	0.601	0.125	1.46E-06	0.855	0.138	5.65E-10	0.869	0.05	1.62E-68
**FINRISK 1997**												

SNP	CHR	Effect allele	Other allele	Beta Females	se females	p-value females	Beta males	se males	p-value males	Beta original	se original	p-value original
**CRP**												
**rs1130864**	**1**	A	G	0.110	0.032	5.06E-04	0.144	0.031	4.14E-06	0.139	0.004	NA
**rs1205**	**1**	T	C	-0.123	0.031	6.83E-05	-0.182	0.031	2.27E-09	-0.184	0.004	NA
**rs3093077**	**1**	C	A	0.211	0.060	3.92E-04	0.245	0.058	2.53E-05	0.218	0.009	NA
**HGF**												
rs3748034	4	T	G	0.115	0.044	8.85E-03	0.186	0.043	1.76E-05	0.153	0.023	5.21E-11
**rs5745687**	**7**	T	C	-0.241	0.078	2.09E-03	-0.321	0.075	1.79E-05	-0.301	0.040	9.92E-14
**MCP-1**												
rs12075	1	A	G	0.067	0.029	2.05E-02	0.082	0.029	4.78E-03	0.219	0.015	1.36E-45
rs138591554	3	T	A	0.360	0.065	2.69E-08	0.328	0.062	1.24E-07	0.321	0.033	1.99E-22
rs2036297	3	G	A	-0.153	0.030	4.20E-07	-0.126	0.030	3.60E-05	-0.118	0.016	1.30E-13
rs2228467	3	C	T	0.296	0.054	4.97E-08	0.330	0.055	2.61E-09	0.262	0.029	1.60E-19
**TRAIL**												
rs11081739	18	G	A	-0.191	0.038	4.68E-07	-0.153	0.039	7.74E-05	-0.140	0.020	4.62E-12
rs138987090	18	G	A	0.838	0.127	4.24E-11	0.687	0.144	1.99E-06	0.726	0.075	2.97E-22
rs183815186	18	T	A	0.301	0.116	9.71E-03	0.322	0.111	3.74E-03	0.350	0.060	6.34E-09
rs193112415	18	C	T	1.140	0.124	4.66E-20	0.962	0.108	6.05E-19	1.046	0.062	1.01E-63
rs57396456	18	C	T	0.546	0.102	8.55E-08	0.509	0.089	1.02E-08	0.564	0.052	7.71E-28
rs62093514	18	T	C	1.042	0.100	1.53E-25	1.019	0.102	1.11E-23	1.046	0.055	5.80E-81
rs62093947	18	C	T	0.840	0.086	1.03E-22	0.913	0.090	4.42E-24	0.758	0.046	2.74E-61
rs6506939	18	C	T	-0.102	0.031	1.20E-03	-0.104	0.032	1.10E-03	-0.095	0.017	1.48E-08
rs679163	18	G	C	-0.104	0.031	8.42E-04	-0.168	0.031	3.75E-08	-0.110	0.016	7.99E-12
rs74778900	18	T	C	0.486	0.101	1.60E-06	0.758	0.108	1.83E-12	0.579	0.053	9.90E-28
rs77451439	18	G	A	0.476	0.070	9.38E-12	0.500	0.069	4.58E-13	0.433	0.037	6.10E-32
**rs79287178**	**3**	A	G	-0.322	0.082	8.44E-05	-0.484	0.075	1.13E-10	-0.430	0.042	1.17E-24
rs9952273	18	T	C	1.220	0.102	6.92E-33	1.059	0.098	2.87E-27	0.869	0.050	1.62E-68
**FINRISK 2002**												
**CRP**												
**rs1130864**	**1**	A	G	0.162	0.050	1.14E-03	0.093	0.049	5.60E-02	0.139	0.004	NA

SNP	CHR	Effect allele	Other allele	Beta Females	se females	p-value females	Beta males	se males	p-value males	Beta original	se original	p-value original
**rs1205**	**1**	T	C	-0.170	0.049	5.49E-04	-0.096	0.051	6.24E-02	-0.184	0.004	NA
**rs3093077**	**1**	C	A	0.268	0.101	7.83E-03	0.095	0.093	3.07E-01	0.218	0.009	NA
**HGF**												
rs3748034	4	T	G	0.139	0.071	5.20E-02	0.227	0.072	1.52E-03	0.153	0.023	5.21E-11
**rs5745687**	**7**	T	C	-0.553	0.136	4.52E-05	-0.340	0.127	7.22E-03	-0.301	0.040	9.92E-14
**MCP-1**												
rs12075	1	A	G	0.020	0.049	6.91E-01	-0.021	0.048	6.63E-01	0.219	0.015	1.36E-45
rs138591554	3	T	A	0.340	0.106	1.33E-03	0.336	0.097	5.31E-04	0.321	0.033	1.99E-22
rs2036297	3	G	A	-0.181	0.049	2.04E-04	-0.055	0.050	2.66E-01	-0.118	0.016	1.30E-13
rs2228467	3	C	T	0.097	0.096	3.15E-01	0.168	0.081	3.74E-02	0.262	0.029	1.60E-19
**TRAIL**												
rs11081739	18	G	A	-0.055	0.065	3.95E-01	-0.107	0.062	8.22E-02	-0.140	0.020	4.62E-12
rs138987090	18	G	A	0.077	0.237	7.44E-01	0.809	0.270	2.77E-03	0.726	0.075	2.97E-22
rs183815186	18	T	A	0.210	0.192	2.73E-01	0.313	0.188	9.55E-02	0.350	0.060	6.34E-09
rs193112415	18	C	T	0.526	0.218	1.56E-02	1.427	0.224	2.08E-10	1.046	0.062	1.01E-63
rs57396456	18	C	T	0.508	0.198	1.02E-02	0.685	0.168	4.50E-05	0.564	0.052	7.71E-28
rs62093514	18	T	C	0.879	0.225	9.33E-05	1.028	0.179	9.10E-09	1.046	0.055	5.80E-81
rs62093947	18	C	T	0.244	0.157	1.20E-01	0.538	0.132	4.34E-05	0.758	0.046	2.74E-61
rs6506939	18	C	T	-0.113	0.052	3.06E-02	-0.057	0.053	2.82E-01	-0.095	0.017	1.48E-08
rs679163	18	G	C	-0.096	0.049	5.01E-02	-0.029	0.051	5.67E-01	-0.110	0.016	7.99E-12
rs74778900	18	T	C	0.321	0.174	6.39E-02	0.594	0.186	1.44E-03	0.579	0.053	9.90E-28
rs77451439	18	G	A	0.213	0.115	6.38E-02	0.321	0.115	5.11E-03	0.433	0.037	6.10E-32
**rs79287178**	**3**	A	G	-0.444	0.141	1.63E-03	-0.585	0.143	4.39E-05	-0.430	0.042	1.17E-24
rs9952273	18	T	C	0.187	0.162	2.46E-01	0.658	0.143	4.33E-06	0.869	0.050	1.62E-68

Beta original, se original and p-value original refer to the original meta-analysed results obtained using complete cohorts. SNPs in bold are cis variants for corresponding inflammatory related variable. CHR, chromosome; SNP, single nucleotide polymorphism; se, standard error

Table S19. B) Meta-analysed gender specific SNP-inflammatory variable associations for SNPs used as instruments for ovarian and breast cancer

SNP	CHR	Effect allele	Other allele	Beta Females	Se females	p-value females	Beta males	Se males	p-value males	Beta combined	se combined	p-value combined	Q	Q p-value	I^2	Gender differentiated p-value	Gender heterog. p-value
**CRP**																	
**rs1130864**	**1**	A	G	0.143	0.023	4.76E-10	0.155	0.023	2.37E-11	0.149	0.016	7.09E-20	7.772	1.69E-01	0.357	7.24E-19	7.16E-01
**rs1205**	**1**	T	C	-0.144	0.023	2.21E-10	-0.184	0.023	1.63E-15	-0.164	0.016	4.99E-24	8.467	1.32E-01	0.409	2.74E-23	2.13E-01
**rs3093077**	**1**	C	A	0.232	0.044	1.73E-07	0.203	0.044	4.26E-06	0.217	0.031	3.78E-12	2.393	7.93E-01	0.000	2.90E-11	6.39E-01
**HGF**																	
rs3748034	4	T	G	0.122	0.033	1.97E-04	0.174	0.033	1.45E-07	0.148	0.023	2.25E-10	3.477	6.27E-01	0.000	9.36E-10	2.58E-01
**rs5745687**	**7**	T	C	-0.246	0.058	2.06E-05	-0.357	0.057	2.79E-10	-0.302	0.040	7.09E-14	11.151	4.85E-02	0.552	2.51E-13	1.68E-01
**MCP-1**																	
rs12075	1	A	G	0.220	0.022	4.04E-24	0.215	0.022	8.97E-23	0.217	0.015	3.18E-45	353.927	0.00E+00	0.986	5.06E-44	8.73E-01
rs138591554	3	T	A	0.337	0.046	4.10E-13	0.293	0.046	2.90E-10	0.315	0.033	9.17E-22	3.257	6.60E-01	0.000	8.29E-21	4.96E-01
rs2036297	3	G	A	-0.130	0.022	5.55E-09	-0.101	0.023	9.13E-06	-0.116	0.016	3.58E-13	8.473	1.32E-01	0.410	2.13E-12	3.69E-01
rs2228467	3	C	T	0.238	0.041	9.66E-09	0.286	0.040	1.34E-12	0.263	0.029	1.07E-19	7.038	2.18E-01	0.290	8.15E-19	4.00E-01
**TRAIL**																	
rs11081739	18	G	A	-0.137	0.028	1.27E-06	-0.142	0.029	9.70E-07	-0.139	0.020	5.67E-12	5.036	4.11E-01	0.007	4.78E-11	9.09E-01
rs138987090	18	G	A	0.757	0.100	3.36E-14	0.672	0.113	3.21E-09	0.720	0.075	7.61E-22	12.125	3.31E-02	0.588	7.41E-21	5.72E-01
rs183815186	18	T	A	0.379	0.085	8.59E-06	0.326	0.085	1.30E-04	0.353	0.060	4.88E-09	4.355	5.00E-01	0.000	3.25E-08	6.58E-01
rs193112415	18	C	T	1.018	0.092	2.05E-28	1.061	0.085	5.00E-36	1.041	0.062	1.09E-62	9.907	7.79E-02	0.495	1.92E-61	7.30E-01
rs57396456	18	C	T	0.472	0.076	6.22E-10	0.647	0.070	3.47E-20	0.567	0.052	6.00E-28	13.654	1.80E-02	0.634	1.83E-27	8.98E-02
rs62093514	18	T	C	1.014	0.079	6.11E-38	1.077	0.077	2.10E-44	1.046	0.055	1.70E-80	2.389	7.93E-01	0.000	3.03E-79	5.69E-01
rs62093947	18	C	T	0.674	0.064	8.07E-26	0.847	0.066	1.21E-37	0.758	0.046	5.63E-61	23.130	3.19E-04	0.784	1.79E-60	6.05E-02
rs6506939	18	C	T	-0.084	0.023	3.19E-04	-0.098	0.024	5.30E-05	-0.091	0.017	6.84E-08	3.432	6.34E-01	0.000	4.28E-07	6.76E-01
rs679163	18	G	C	-0.096	0.023	2.03E-05	-0.131	0.023	1.48E-08	-0.113	0.016	2.43E-12	6.802	2.36E-01	0.265	1.19E-11	2.86E-01
rs74778900	18	T	C	0.406	0.071	1.04E-08	0.799	0.081	3.46E-23	0.577	0.053	2.02E-27	18.419	2.46E-03	0.729	3.05E-29	2.44E-04
rs77451439	18	G	A	0.408	0.052	4.84E-15	0.457	0.052	2.18E-18	0.432	0.037	9.68E-32	6.107	2.96E-01	0.181	1.06E-30	5.05E-01
**rs79287178**	**3**	A	G	-0.375	0.061	8.76E-10	-0.473	0.058	3.50E-16	-0.427	0.042	3.80E-24	3.725	5.90E-01	0.000	2.32E-23	2.45E-01
rs9952273	18	T	C	0.820	0.071	7.60E-31	0.912	0.070	4.99E-39	0.867	0.050	6.01E-68	40.141	1.40E-07	0.875	7.66E-67	3.59E-01

Meta-analysis results of gender-specific SNP-inflammatory variable associations across the three cohorts obtained using GWAMA software (https://genomics.ut.ee/en/tools/gwama) with –sex option enabled. Gender differentiated p-value refers to combined p-value of females and males assuming different effect sizes between genders and gender heterogeneity p-value to heterogeneity between genders. SNPs in bold are cis variants for corresponding inflammatory related variable. CHR, chromosome; SNP, single nucleotide polymorphism; se, standard error; Q, Cochran's heterogeneity statistic; I^2, Higgins' heterogeneity index

References

1 Raitakari OT, Juonala M, Ronnemaa T, Keltikangas-Jarvinen L, Rasanen L, Pietikainen M *et al.* Cohort Profile: The Cardiovascular Risk in Young Finns Study. *Int J Epidemiol* 2008; **37**: 1220–1226.

2 Ahola-Olli A V., Würtz P, Havulinna AS, Aalto K, Pitkänen N, Lehtimäki T *et al.* Genome-wide Association Study Identifies 27 Loci Influencing Concentrations of Circulating Cytokines and Growth Factors. *Am J Hum Genet* 2017; **100**: 40–50.

3 Santalahti K, Maksimow M, Airola A, Pahikkala T, Hutri-Kähönen N, Jalkanen S *et al.* Circulating cytokines predict the development of insulin resistance in a prospective Finnish population cohort. *J Clin Endocrinol Metab* 2016; **101**: 3361–3369.

4 Santalahti K, Havulinna A, Maksimow M, Zeller T, Blankenberg S, Vehtari A *et al.* Plasma levels of hepatocyte growth factor and placental growth factor predict mortality in a general population: a prospective cohort study. *J Intern Med* 2017; **282**: 340–352.

5 Hemani G, Zheng J, Wade KH, Laurin C, Elsworth B, Burgess S *et al.* MR-Base: a platform for systematic causal inference across the phenome using billions of genetic associations. 2017. doi:https://doi.org/10.1101/078972.

6 Locke AE, Kahali B, Berndt SI, Justice AE, Pers TH, Day FR *et al.* Genetic studies of body mass index yield new insights for obesity biology. *Nature* 2015; **518**: 197–206.

7 Ligthart S, Vaez A, Võsa U, Stathopoulou MG, de Vries PS, Prins BP *et al.* Genome Analyses of >200,000 Individuals Identify 58 Loci for Chronic Inflammation and Highlight Pathways that Link Inflammation and Complex Disorders. *Am J Hum Genet* 2018; **103**: 691–706.

8 Burgess S, Butterworth A, Thompson SG. Mendelian Randomization Analysis With Multiple Genetic Variants Using Summarized Data. *Genet Epidemiol* 2013; **37**: 658–665.

9 Bowden J, Davey Smith G, Burgess S. Mendelian randomization with invalid instruments: effect estimation and bias detection through Egger regression. *Int J Epidemiol* 2015; **44**: 512–525.

10 Brion MJA, Shakhbazov K, Visscher PM. Calculating statistical power in Mendelian randomization studies. *Int J Epidemiol* 2013; **42**: 1497–1501.

11 Burgess S. Sample size and power calculations in Mendelian randomization with a single instrumental variable and a binary outcome. *Int J Epidemiol* 2014; **43**: 922–929.

12 Nikpay M, Goel A, Won HH, Hall LM, Willenborg C, Kanoni S *et al.* A comprehensive 1,000 Genomes-based genome-wide association meta-analysis of coronary artery disease. *Nat Genet* 2015; **47**: 1121–1130.

13 Schunkert H, Konig IR, Kathiresan S, Reilly MP, Assimes TL, Holm H *et al.* Large-scale association analysis identifies 13 new susceptibility loci for coronary artery disease. *Nat Genet* 2011; **43**: 333–338.

14 Mahajan A, Go MJ, Zhang W, Below JE, Gaulton KJ, Ferreira T *et al.* Genome-wide trans-ancestry meta-analysis provides insight into the genetic architecture of type 2 diabetes susceptibility. *Nat Genet* 2014; **46**: 234–244.

15 Liu JZ, van Sommeren S, Huang H, Ng SC, Alberts R, Takahashi A *et al.* Association analyses

identify 38 susceptibility loci for inflammatory bowel disease and highlight shared genetic risk across populations. *Nat Genet* 2015; **47**: 979–986.

16 Okada Y, Wu D, Trynka G, Raj T, Terao C, Ikari K *et al.* Genetics of rheumatoid arthritis contributes to biology and drug discovery. *Nature* 2014; **506**: 376–381.

17 Lambert JC, Ibrahim-Verbaas CA, Harold D, Naj AC, Sims R, Bellenguez C *et al.* Meta-analysis of 74,046 individuals identifies 11 new susceptibility loci for Alzheimer's disease. *Nat Genet* 2013; **45**: 1452–1458.

18 Schizophrenia Working Group of the Psychiatric Genomics C, Consortium SWG of the PG. Biological insights from 108 schizophrenia-associated genetic loci. *Nature* 2014; **511**: 421–427.

19 Michailidou K, Lindström S, Dennis J, Beesley J, Hui S, Kar S *et al.* Association analysis identifies 65 new breast cancer risk loci. *Nature* 2017; **551**: 92–94.

20 Phelan CM, Kuchenbaecker KB, Tyrer JP, Kar SP, Lawrenson K, Winham SJ *et al.* Identification of 12 new susceptibility loci for different histotypes of epithelial ovarian cancer. *Nat Genet* 2017; **49**: 680–691.
